# In host evolution of *Exophiala dermatitidis* in cystic fibrosis lung micro-environment

**DOI:** 10.1093/g3journal/jkad126

**Published:** 2023-06-09

**Authors:** Tania Kurbessoian, Daniel Murante, Alex Crocker, Deborah A Hogan, Jason E Stajich

**Affiliations:** Department of Microbiology and Plant Pathology and Institute of Integrative Genome Biology, University of California Riverside, Riverside, CA 92521, USA; Department of Microbiology and Immunology, Geisel School of Medicine, Dartmouth College, Hanover, NH 03755, USA; Department of Microbiology and Immunology, Geisel School of Medicine, Dartmouth College, Hanover, NH 03755, USA; Department of Microbiology and Immunology, Geisel School of Medicine, Dartmouth College, Hanover, NH 03755, USA; Department of Microbiology and Plant Pathology and Institute of Integrative Genome Biology, University of California Riverside, Riverside, CA 92521, USA

**Keywords:** *Exophiala dermatitidis*, black yeast, cystic fibrosis, population genomics

## Abstract

Individuals with cystic fibrosis (CF) are susceptible to chronic lung infections that lead to inflammation and irreversible lung damage. While most respiratory infections that occur in CF are caused by bacteria, some are dominated by fungi such as the slow-growing black yeast *Exophiala dermatitidis*. Here, we analyze isolates of *E. dermatitidis* cultured from two samples, collected from a single subject 2 years apart. One isolate genome was sequenced using long-read Nanopore technology as an in-population reference to use in comparative single nucleotide polymorphism and insertion–deletion variant analyses of 23 isolates. We then used population genomics and phylo-genomics to compare the isolates to each other as well as the reference genome strain *E. dermatitidis* NIH/UT8656. Within the CF lung population, three *E. dermatitidis* clades were detected, each with varying mutation rates. Overall, the isolates were highly similar suggesting that they were recently diverged. All isolates were MAT 1-1, which was consistent with their high relatedness and the absence of evidence for mating or recombination between isolates. Phylogenetic analysis grouped sets of isolates into clades that contained isolates from both early and late time points indicating there are multiple persistent lineages. Functional assessment of variants unique to each clade identified alleles in genes that encode transporters, cytochrome P450 oxidoreductases, iron acquisition, and DNA repair processes. Consistent with the genomic heterogeneity, isolates showed some stable phenotype heterogeneity in melanin production, subtle differences in antifungal minimum inhibitory concentrations, and growth on different substrates. The persistent population heterogeneity identified in lung-derived isolates is an important factor to consider in the study of chronic fungal infections, and the analysis of changes in fungal pathogens over time may provide important insights into the physiology of black yeasts and other slow-growing fungi in vivo.

## Introduction

Cystic fibrosis (CF) is an autosomal recessive disorder caused by mutations in the cystic fibrosis transmembrane regulator (CFTR) gene that impairs the balance of salts and water across epithelia. In the lungs, these ion transport defects cause viscous mucus, which contributes to respiratory infections that cause most of the morbidity and mortality in CF populations (Riordan *et al.* 1989; Davis 2006; Ferec and Cutting 2012). Microbial colonization of mucosal plugs results in recurring infection and inflammation that cause irreversible lung damage and declining lung function (Turcios 2020). Bacteria, particularly *Staphylococcus aureus*, *Pseudomonas aeruginosa*, *Burkholderia cepacia*, and *Stenotrophomonas maltophilia*, are pathogens that frequently dominate CF respiratory infections (Burns *et al.* 1998; Mariani-Kurkdjian and Bingen 2003; Horré *et al.* 2004; Steinkamp *et al.* 2005; Tunney *et al.* 2008; Pihet *et al.* 2009; Zhao *et al.* 2012) and are sometimes isolated with various species of fungi. Clinically significant fungi in CF lung infections include *Exophiala dermatitidis*, *Scedosporium apiospermum*, *Aspergillus fumigatus*, *Candida albicans*, and *Clavispora (Candida) lusitaniae* (Kusenbach *et al.* 1992; Cimon *et al.* 2000; Defontaine *et al.* 2002; Horré *et al.* 2004, 2009; Parize *et al.* 2014; Chen *et al.* 2018; Demers *et al.* 2018; Jong *et al.* 2020). The consequences of fungal infection on CF outcomes is not well understood but is influenced by the genotypes of both the host and microbes (Burns *et al.* 1998; Nagano *et al.* 2007; Pihet *et al.* 2009; Packeu *et al.* 2012).

*Exophiala dermatitidis*, which has been previously named *Hormiscium dermatitidis* and *Wangiella dermatitidis*, is taxonomically classified in Phylum Ascomycota, Order Chaetothyriales, Family Herpotrichiellaceae. To date, about 40 species in the *Exophiala* genus have been identified, 17 of which are known to cause disease in mammals. Among these, *E. dermatitidis* is the most clinically prevalent with reported mortality rates of 25–80% in systemic and invasive cases, even though fatal systemic cases are relatively rare (Kirchhoff *et al.* 2019). Clinical presentations of this fungus include phaeohyphomycosis, keratitis, chromoblastomycosis, and even several neural diseases and meningitis (Matos *et al.* 2002; Revankar *et al.* 2002; Uijthof *et al.* 2009; Revankar and Sutton 2010; Seyedmousavi *et al.* 2014; Song *et al.* 2017; Kirchhoff *et al.* 2019; Lavrin *et al.* 2020). The first isolate of *E. dermatitidis* from a CF patient was obtained in 1990 by culturing from sputum (Haase *et al.* 1990; Kusenbach *et al.* 1992). Multiple documented cases of *E. dermatitidis* in CF patients have followed (Rath *et al.* 1997; Diemert *et al.* 2001; Horré *et al.* 2004; Griffard *et al.* 2010; Packeu *et al.* 2012), some of which have developed into late-stage mycosis (Sudfeld *et al.* 2010; Kondori *et al.* 2011; Song *et al.* 2017; Grenouillet *et al.* 2018), with some infections benefiting from treatment (Packeu *et al.* 2012; Mukai *et al.* 2014) while some disease can lead to death (Klasinc *et al.* 2019).

*Exophiala* species are morphologically grouped in the so-called “black yeasts”, which are not a taxonomic group but are classified by three defining features. Black yeasts produce melanin through 1–8 dihydroxynapthalene (DHN) biosynthesis pathway, exhibit morphological plasticity or meristematic growth (yeast cells, hyphae, or even pseudohyphae), and have membrane-associated carotenoids and intracellular mycosporine-like amino acids (Hoog *et al.* 2000; Nosanchuk and Casadevall 2003, 2006; Saunte *et al.* 2012; Smith and Casadevall 2019). All of these properties of melanin likely contribute to the extreme resistance to environmental stresses including desiccation, UV or solar exposure, and ionizing radiation. These resistance traits may also contribute to fungal success in growth in mammalian hosts and their ability to cause disease in susceptible hosts. To better study the molecular biology and genetics of this species, the *E. dermatitidis* strain NIH/UT8656 (ATCC 34100, CBS 525.76) genome was sequenced and assembled into 11 scaffolds presumed to correspond to chromosomes (Robertson *et al.* 2012; Chen *et al.* 2014; Schultzhaus *et al.* 2020; Malo *et al.* 2021). This genomic resource has enabled evolutionary and comparative genomics studies of the species, and identification of genes that may underlie its resilience (Robertson *et al.* 2012; Schultzhaus *et al.* 2020; Malo *et al.* 2021) and success in human host colonization (Kondori *et al.* 2011; Kirchhoff *et al.* 2019).

Reservoirs of *E. dermatitidis* have long been associated with hot and humid tropical origins (Sudhadham *et al.* 2008). *Exophiala dermatitidis* are isolated from many man-made substrates such as humidifiers (Nishimura and Miyaji 1982), saunas (Matos *et al.* 2002), and dishwashers (Zupančič *et al.* 2016; Babič *et al.* 2018). Babič *et al.* (2018) concluded that *Exophiala* tends to be found in locations with oligotrophic conditions or where rubber seals and humidity act as an enrichment or trapping mechanism, which supports *Exophiala* persistence. *Exophiala dermatitidis* and related species are also found in broad environmental niches including wasp nests (Conti-Díaz *et al.* 1977), healthy bats (Reiss and Mok 1979), lesions of toads (Frank and Roester 1970), and rotten wood (Dixon *et al.* 1980), which suggests human patients could acquire infections from environmental exposure (Sudhadham *et al.* 2011).

In light of several studies that chronic CF-related fungal infections diversify over time (Kim *et al.* 2015; Demers *et al.* 2018; Jones *et al.* 2021; Ross *et al.* 2021), here we report both phenotypic and genomic diversity among *E. dermatitidis* isolates from a single individual. Population genomic analyses identified multiple lineages that persisted over 2 years. This dataset allows us to test the hypothesis that, as seen in previous studies, the CF lung environment supports stably diverged populations of a clonally derived yeast.

## Methods

### Sputum-derived isolate cultures

Frozen sputum was obtained from a specimen bank in which samples were obtained in accordance with protocols approved by the Dartmouth-Hitchcock Institutional Review Board. Aliquots of sputum were plated onto Sabouraud dextrose agar medium as described previously (Grahl *et al.* 2018). Individual isolates were obtained and banked; isolate identifiers are listed in Supplementary Table 1. Isolates were stored as frozen stocks with 25% glycerol and maintained at −80˚C and subcultured on solid YPD. Strains were grown in liquid YPD overnights for 18 h prior to use in experiments.

### DNA extraction and sequencing

*Exophiala* isolates were grown in yeast peptone dextrose media (YPD) for ∼24 h in 5-ml roller-drum cultures at 37 °C. Cells were spun down for 5 min at 5,000 RCF and washed thrice with deionized water. Genomic DNA was extracted from cell pellets using the MasterPure yeast DNA purification kit (Epicentre). Melanin was removed from genomic DNA using the *OneStep* PCR Inhibitor Removal kit (Zymo Research). Genomic DNA was measured by Nanodrop and diluted to ∼20 ng/µl. DNA extractions were sent to Novogene, (Novogene Corporation Inc., Sacramento, California) for 2 × 150 bp sequencing on an Illumina NovoSeq 6000. DNA from isolate DCF04 was extracted and sequenced on Oxford Nanopore (ONT) platform with library preparation and sequencing following the manufacturer's directions (Oxford Nanopore, Oxford United Kingdom). Flow cells with R9.1.4 chemistry on a MinION followed base-calling with Guppy (*v. 3.4.4 + a296cb*) run with High Accuracy (HAC) model (Wick *et al.* 2019).

### Genome assembly and annotation

Genome assemblies were constructed for the 23 *E. dermatitidis* isolates from Illumina sequencing. One isolate, Ex4, was also re-sequenced using ONT technology. All genomes were de novo assembled with AAFTF pipeline (*v.0.2.3*) (Palmer and Stajich 2022), which performs read QC and filtering with BBTools BBDuk (*v.38.86)* (Bushnell 2014) followed by SPAdes (*v.3.15.2*) (Bankevich *et al.* 2012) assembly using default parameters, followed by screening to remove short contigs <200 bp and contamination using NCBI's VecScreen. The BUSCO ascomycota_odb9 database (Manni *et al.* 2021) was used to determine how complete the assembly was for all 23 isolates of *E. dermatitidis.* A hybrid assembly of isolate DCF04/Ex4 was generated using MaSuRCA (*v.3.3.4*) (Zimin *et al.* 2013) as the assembler using both Nanopore and Illumina sequencing reads. General default parameters were used except: CA_PARAMETERS = cgwErrorRate = 0.15, NUM_THREADS = 16, and JF_SIZE = 200,000,000. The updated genome was then scaffolded to strain NIH/UT8656 accession GCF_000230625 using Ragtag (*v.1.0.2*) (Alonge *et al.* 2019), which uses minimap2 (*v. 2.17-r941)* (Li 2018) to further link scaffolds based on shared co-linearity of these isolates’ genomes. Assembly summary statistics were calculated with the “assess” tool in AAFTF and genome completeness by BUSCO (*v5.4.4*) (Manni *et al.* 2021) with the eurotiomycetes_odb10 database of 290 marker genes.

We predicted genes in this near-complete genome assembly with Funannotate (*v1.8.1*) (Palmer and Stajich 2020). A masked genome was created by first generating a library of sequence repeats with the RepeatModeler pipeline (Smit and Hubley 2008). These species-specific predicted repeats were combined with fungal repeats in the RepBase (Bao *et al.* 2015) to identify and mask repetitive regions in the genome assembly with RepeatMasker (*v.4-1-1*) (Smit 2004). To predict genes, ab initio gene predictors SNAP (*v.2013_11_29*) (Korf 2004) and AUGUSTUS (*v.3.3.3*) (Stanke *et al.* 2006) were trained using the Funannotate “train” command based on the full-length transcripts constructed by Genome-Guided run of Trinity (*v.2.11.0*) (Grabherr *et al.* 2011) using RNA-Seq from published *E. dermatitis* Sequence Read Archive accession SRR12590377 under BioProject PRJNA660011. The assembled transcripts were aligned with PASA (*v.2.4.1*) (Haas *et al.* 2008) to produce full-length spliced alignments and predicted open reading frames for training the ab initio predictors and as informant data for gene predictions. Additional gene models were predicted by GeneMark.HMM-ES (*v.4.62_lic*) (Brůna *et al.* 2020), and GlimmerHMM (*v.3.0.4*) (Majoros *et al.* 2004) utilize a self-training procedure to optimize ab initio predictions. Additional exon evidence to provide hints to gene predictors was generated by DIAMOND BLASTX alignment of SwissprotDB proteins and polished by Exonerate (*v.2.4.0*) (Slater and Birney 2005). Finally, EvidenceModeler (*v.1.1.1*) (Haas *et al.* 2008) generated consensus gene models in Funannotate were constructed using default evidence weights. Nonprotein-coding tRNA genes were predicted by tRNAscan-SE (*v.2.0.9*) (Lowe and Chan 2016). Ribosomal rRNA genes were predicted using RepeatMasker (*v.4-1-1*) (Smit 2004).

The annotated genome was processed with antiSMASH (*v.5.1.1*) (Blin *et al.* 2021) to predict secondary metabolite biosynthesis gene clusters. These annotations were also incorporated into the functional annotation by Funannotate. Putative protein functions were assigned to genes based on sequence similarity to InterProScan5 (*v.5.51-85.0*) (Jones *et al.* 2014), Pfam (*v.35.0*) (Finn *et al.* 2014), Eggnog (*v.2.1.6-d35afda*) (Huerta-Cepas *et al.* 2019), dbCAN2 (*v.9.0*) (Zhang *et al.* 2018) and MEROPS (*v.12.0*) (Rawlings *et al.* 2018) databases relying on NCBI BLAST (*v.2.9.0+)* (Sofi *et al.* 2022), and HMMer (*v.3.3.2*) (Potter *et al.* 2018). Gene Ontology terms were assigned to protein products based on the inferred homology based on these sequence similarity analyses.

Copy number variation (CNV) was examined by plotting 10-kbp window-based read coverage of the short-read alignments of each isolate. The depth of coverage was calculated using Mosdepth (Pedersen and Quinlan 2018), and visualized with R using the ggplot2 package (Wickham 2016).

The mating-type (MAT) locus analysis followed previous work done by Metin *et al.* (2019) was identified through searching for HMG-box homologs of the MAT genes HMG-box HMPREF1120_08862 (MAT1-2) and HMPREF1120_05727 (APN2), in this study's 23 *E dermatitidis* isolate genomes with cblaster (Gilchrist *et al.* 2021). A homothallic black yeast, *Capronia coronata* CBS 617.96 (AMWN00000000.1) (Teixeira *et al.* 2017), which has both MAT genes, was also incorporated into the analyses and visualization. The identified homologous regions were examined for their conserved synteny of the MAT locus using clinker (Gilchrist and Chooi 2021) and a custom Biopython script (Cock *et al.* 2009; Kurbessoian 2022) to extract the annotated region of the genome that contained the locus.

Identification of telomeric repeat sequences was performed using FindTelomeres.py script (Hiltunen *et al.* 2021). Briefly, this searches for chromosomal assembly with a regular expression pattern for telomeric sequences at the 5′ and 3′ end of each scaffold.

### Identification of sequence variation

Sequence variation among isolates was assessed using the best practices of the Genome Analysis ToolKit GATK (*v. 4.0.4.0*) (McKenna *et al.* 2010; Franke and Crowgey 2020) to identify SNPs and insertion/deletions (INDELs). Illumina paired-end reads were aligned to the isolate DCF04 genome with BWA (*v.0.7.17*) (Li and Durbin 2010) and processed with Samtools (*v.1.8*) (Li *et al.* 2009) and Picard Toolkit (Institute; Toolkit) AddOrReplaceReadGroups and MarkDuplicates (*v.2.18.3*) tools. The alignments were further improved by realigning reads near inferred INDELs using GATK tools RealignerTargetCreator, IndelRealigner, and PrintReads. Genotypes were inferred with the GATK Haplotype and GenotypeGVCF methods to produce a single VCF file of the identified variants. Low-quality SNPs were further filtered using GATK VariantFiltration, and finally SelectVariants was used with the parameters: mapping quality (score <40), quality by depth (<2 reads), Strand Odds Ratio (>4.0), Fisher Strand Bias (>200), and Read Position Rank Sum Test (< −20) to retain only high-quality polymorphisms. Finally, an additional stringent series of three filtering steps implemented in bcftools (*v. 1.12*) (Li *et al.* 2019) was used on the VCF file to remove calls that were below the 1,000 quality score threshold, where any individual isolate had a “no call”, and where the standard deviation in read depth was above or below a standard deviation value of 1 for an individual SNP. SnpEff (*v.4.3r*) (Cingolani *et al.* 2012) was used to score the impact of the identified variants using the Funannotate annotated DCF04 genome GFF3 file.

Variant calling was performed on two sets of individuals, one limited to the 23 CF patient population isolates with reads aligned to the DCF04 isolate and one using the *E. dermatitidis* strain NIH/UT8656 reference genome. Pairwise isolate comparisons of SNP and INDEL were counted to generate isolate correlation heatmaps for both variant types using a UPGMA clustering. A custom script make_diagonal.sh uses plink (*v.2.00a3LM_AVX2*) (Chang *et al.* 2015) to count all pairwise differences between individuals in the VCF files stratified by SNPs or INDELs. A custom Perl script transformed pairwise counts into a matrix of isolated differences observed for both SNP and INDEL variants. Counts were summarized as heatmaps with an R script. All scripts developed for this manuscript are available at the Github (Kurbessoian 2022) project linked in this paper.

A calculation of the population mutation rate was performed on each isolate based on the number of SNPs shared among a pair of isolates. The formula to calculate the mutation rate per year for each isolate is as follows: (Pairwise SNP Count)/(Adjusted Genome Length)/Pair/Year. The time between isolated collections was 22 months. The value used for the “Adjusted Genome Length” was collected from genome assembly statistics of DCF04 (*v.0.2.3*) (Palmer and Stajich 2022). A one-way ANOVA was run on the grouped calculated mutation rates for each isolate to determine significance.

A comparison of the DNA similarity of DCF04 and NIH/UT8656 genomes was visualized with a dot-plot constructed with D-GENIES (Cabanettes and Klopp 2018). The tool was run using minimap2 and default parameters on the tool's website (https://dgenies.toulouse.inra.fr).

Ortholog and paralog assignment of genes between the annotated DCF04 and NIH/UT8656 genomes was calculated with Orthofinder (Emms and Kelly 2019). First, genes with alternatively spliced isoforms were reduced to the single longest isoform. The pairwise similarity of proteins was computed by DIAMOND with ultra-sensitive parameters (Buchfink *et al.* 2015). Assessment of shared prediction protein-coding genes was extracted from the Orthology tables generated by Orthofinder and a custom Python script. Singleton proteins generated from Orthofinder results were assessed from both DCF04 and NIH/UT8656 using the proteins as a query and e-value set to 1e-5 with TBLASTN from the NCBI-BLAST toolkit and miniprot (Li 2023).

### Phylogenetic relationships of the isolates

SNPs from polymorphic sites were extracted from the VCF files as multi-fasta files using BCFTools (Li *et al.* 2019) and a custom script make_strain_tree.sh. A maximum-likelihood phylogenetic tree was constructed from the multi-fasta file using IQTree (*v. 2.0.4*) (Minh *et al.* 2020) and the model parameters to accurately model the ascertainment bias of a SNP-only dataset [-m GTR + ASC]. The nucleotide substation model GTR + F + ASC selected based on Bayesian information criteria runs within IQTree. Statistical support for the tree nodes was evaluated from 1,000 bootstrap replicates using UFBoot ultra-fast bootstrapping approximation (Hoang *et al.* 2018). The tree was visualized using iTOL (Letunic and Bork 2016).

### Minimum inhibitory concentration assays and spot titers

*Exophiala* isolates were struck from −80˚C glycerol stocks onto yeast extract peptone dextrose (YPD) plates (2% glucose, 2% yeast extract, 1% peptone) and allowed to grow for 48 h at 37 °C. Overnight cultures were started from YPD patches inoculated into 5 ml of liquid YPD and grown for ∼24 h in a roller drum at 30 °C. For minimum inhibitory concentration (MIC) assays, cultures were spun down for 5 min at 5,000 RCF and washed thrice in deionized water. Cells were counted on a hemocytometer and added to a final concentration of 1 × 10^3^ cells per well in a 96-well flat-bottom dish, then grown at 37˚C for 72 h before determining the final MIC through visual inspection for lack of growth. For spot titers on rich and minimal media with and without the supplementation of 2% casamino acids, cells were CFU equilibrated to 1 × 10^6^ CFU/ml, and serial dilutions (1:10) were spotted in 5-µl volumes. Plates were incubated for 48 h at 37˚C prior to imaging.

### Plate-based melanization assessment

*Exophiala* isolates were initially cultured as described above onto YPD plates and arranged in order of isolate number. Plates were incubated at 37˚C for 5 days, to permit for sufficient growth and melanin production. Top-down images of each plate were captured using a Canon EOS Rebel T6i with an 18- to 15-mm lens.

### Microscopy

*Exophiala* isolates were initially cultured onto solid media and liquid overnights as described above. From confluent overnights, isolates were inoculated at 1,000 CFU/ml into RPMI-1640 in glass-bottom 24-well culture dishes and incubated at 37˚C in 5% CO for 18 h. Cultures were then imaged at 63X magnification, DIC with a Zeiss Axiovert 200 M inverted microscope.

## Results

**Fig. 1.**
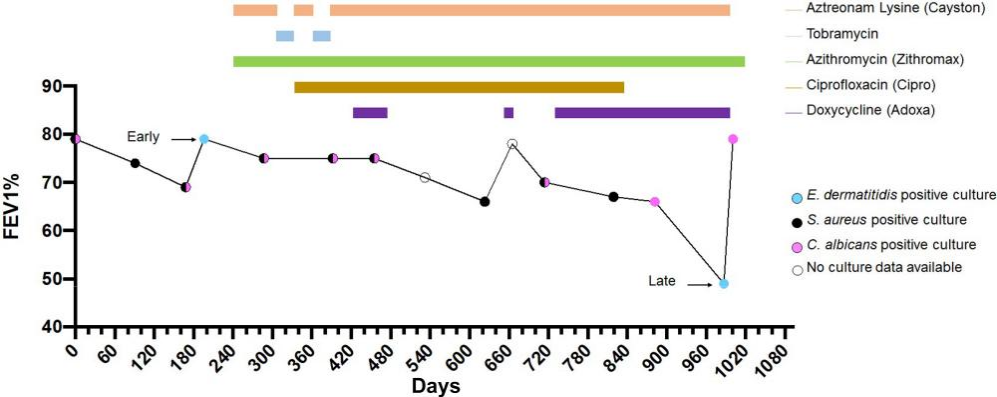
FEV1 pulmonary function data, antibiotic use, and sputum collection time points. FEV1 (forced expiratory volume in one second), an indication of pulmonary function, was measured over 3 years. Sputum samples were acquired at each indicated time point and assessed for the presence of pathogens other than the mixed bacteria that compose normal upper respiratory flora. Horizontal bars indicate the duration of treatment for each listed antibiotic given during the study period.

Molecular and culture-based analysis of a series of sputum samples identified an individual with CF with a chronic lung infection caused by *E. dermatitidis* (Grahl *et al.* 2018)*. Exophiala dermatitidis* isolates were recovered from banked sputum samples, collected 2 years apart. *Staphylococcus aureus* and *Candida albicans* were also identified in clinical cultures from the patient in the intervening years between the two time points (Fig. 1). The subject's antimicrobial use history included Aztreonam, Azithromycin, Tobramycin, Ciprofloxacin, and Doxycycline, and the patient's lung function, measured by percent predicted forced expiratory volume (%FEV1), ranged between 80% and 49% during this time period. While *E. dermatitidis* was not detected in the first clinical microbiological analysis, perhaps due to its extremely slow growth out of clinical samples (Grahl *et al.* 2018) or suppression by bacteria, it was detected in the second clinical analysis. Twenty-three isolates (11 from the early time point and 12 from the late time point) were selected for further population genomic study.

### Sequencing and assembly of *E. dermatitidis* isolates

To gain information on the genetic variation and potential population structure for the recovered *E. dermatitidis* isolates, we sequenced and assembled the genomes of the 23 isolates (Supplementary Table 2). The depth of coverage ranged from 11 to 47× coverage across all 23 Illumina sequenced samples. Benchmarking Universal Single-Copy Orthologs (BUSCO) was used to test genome assembly completeness; within our dataset, it ranged 99–99.3% complete, 98.3–99.3% single copies present, 0–1% for duplicates, and 0–0.3% missing BUSCOs. Contig counts spanned 44–1,422 and the average genome assembly size is 26.7 Mbp, N50 of 1,078,277, and L50 of 9.21. Repeat content for all 23 strains ranged from 3.8% to 4.5%, while strain NIH/UT8656 was 3.5%, considerably lower than the CF lung isolates.

To further examine the fine-scale variation within the CF isolates, we sought to generate a within-population high-quality reference genome. DNA sequence reads for isolate DCF04 were generated using ONT sequenced with an average genome depth of coverage of 11×. A hybrid genome assembly was constructed from the combined ONT and Illumina reads (Supplementary Table 2) to produce a 26.6-Mbp assembly. Only 3.19% of the genome was identified as repetitive elements. The candidate telomeric repeat units “TTTAGGG/CCCTAA” were identified as arrays of repeats at both ends of five of the scaffolds and as repeats on only one end of the remaining three scaffolds. (Supplementary Table 3). A total of 9,599 genes were predicted comprising 64 tRNA genes and 9,535 protein-coding genes, of which 426 have at least two alternative isoforms predicted based on RNA-seq evidence. A search for secondary metabolite gene clusters with antiSMASH identified 15 secondary metabolite clusters, of which four are classified as terpenes, seven as NRPS containing, two T1PKS, 1 T3PKS, and one betalactone cluster. RepeatMasker identified rDNA copy numbers vary from 18 to 22 found in 23 *E. dermatitidis* CF isolates and 22 copies in strain NIH/UT86556.

### Comparing assembly quality and gene content of DCF04 to NIH/UT8656

We sought to examine how similar the assembled genome content and gene content is between two fungal isolates cultured from different environments and sequenced with different technologies. The isolate DCF04 was isolated from the lung sputum of a CF patient and sequenced with Illumina and ONT technology and the NIH/UT8656 isolate, likely cultured from a skin infection, and the genome was sequenced with Sanger sequencing technology (BioProject: PRJNA225511, Assembly: GCF_000230625.1). To assess the differences between the two genome assemblies, we compiled summary statistics (Supplementary Table 2) and interrogated the predicted gene content of both genomes. The DCF04 assembly had 165 contigs linked into 44 scaffolds, while NIH/UT8656 comprised 238 contigs linked into 11 scaffolds. The DCF04 scaffolds were derived by a comparative assembly against the NIH/UT8656 assembly to achieve the best assembly after first testing for any evidence of rearrangements. The total length of the genome assemblies is nearly the same where DCF04 is 26.6 Mb and NIH/UT8656 is 26.4 Mb. The summary statistics for scaffold L50 and N50 are also nearly identical for DCF04 at 4 and 3.7 Mb and NIH/UT8656 were 4 and 3.6 Mb, respectively. The BUSCO completeness statistics search with ascomycota_odb10 (1,706 models) identified DCF04 as 99.6% complete and NIH/UT8656 as 99.4% complete. A dot-plot comparing the two genome assemblies revealed minimal rearrangements or discontinuity suggesting high similarity of the two isolated genomes (Supplementary Figure 1).

The gene content was further compared using OrthoFinder (Emms and Kelly 2015; Emms and Kelly 2019) (Supplementary Table 4), which identified 8,256 orthologous groups comprising 17,640 orthologous protein-coding genes between DCF04 and NIH/UT8656. A total of 1,180 singleton genes were detected through OrthoFinder and can be found in the Othrogroups_UnassigedGenes.tsv file. Seven hundred and five singleton genes were identified in the DCF04 strain and 475 in NIH/UT8656 strain. In addition, some gene families were multicopy, but lineage-specific to a strain as found in the Orthologs.tsv file where only a single strain was found. Ten families were multicopy in DCF04 and 5 in NIH/UT8656.

To test the hypothesis that genes classified as lineage-specific were truly unique to a strain or a product of missing annotation in the other isolate, we performed translated searches of the predicted proteins with TBLASTN and miniprot against each of the isolate genome assemblies.

This search found that 683/705 (96.9%) of the putative DCF04 lineage-specific loci had identifiable similarities in the NIH/UT8656 strain genome assembly so that only 22 genes are truly DCF04 lineage-specific genes (Supplementary Table 4). The reciprocal analysis found that 468/475 (98.5%) of the putative NIH/UT8656 lineage-specific loci had identifiable similarities in the CF isolate genomes. Only seven genes were found to be NIH/UT8656 lineage-specific genes and were missing in all 23 isolates of *E. dermatitidis* from this study. Overall, we find the majority of the gene content differences reflect variation in predictions produced by gene annotation pipelines consistent with observations in other systems (Weisman *et al.* 2022).

### All Ex CF isolates are MAT1-1 mating type

**Fig. 2.**
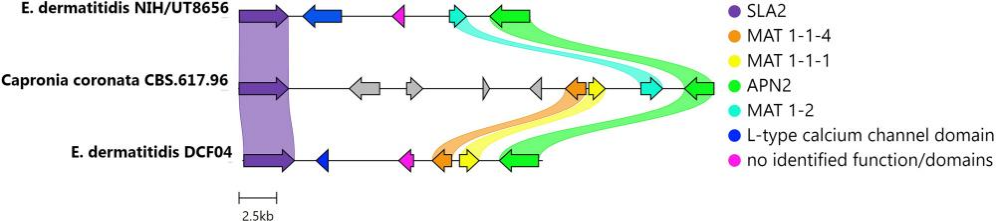
Mating-type determination of four clinical isolates of *E. dermatitidis.* Gene content, order and orientation of the MAT locus from *E. dermatitidis* DCF04 and NIH/UT8656 strains, and the Chaetothyriales black yeast *Capronia coronata* CBS 617.98. The locus is flanked by two genes SLA2 and APN2. The MAT 1-1 genes, MAT 1-1-4, and MAT 1-1-1 are observed in DCF04 isolate, while the reference strain NIH/UT8656 has a MAT 1-2 gene. In addition, two genes are predicted in the interval between SLA2 and the MAT genes. One is an L-type calcium channel domain, which is only found in *E. dermatitidis*, and a gene with no identified function or domains, which is syntentically adjacent to the MAT genes in all isolates and among all species.

To examine and genotype the mating-type locus in the isolates, we searched for homologs of the ascomycete MAT genes with the tool cblaster and visualized their shared microsynteny with clinker. The genomes of all the CF isolates including DCF04/Ex4 *E. dermatitidis* isolate encoded a MAT1-1 locus (Fig. 2, Supplementary Figure 2, and Supplementary Table 5). Consistent with previous MAT loci described in *E. dermatitidis* (Metin *et al.* 2019), DCF04 had both MAT1-1-4 and MAT1-1-1 genes (Fig. 2). The NIH/UT8656 encodes a MAT1-2 mating type in contrast to the uniform MAT1-1 type found in all CF clinical isolates.

Examination of genomes of additional *E. dermatitidis* isolates and *Capronia* species identified homologs of *SLA2*, a SRC-like adapter protein, and *APN2*, apurinic-apyrimidinic endonuclease 2, flanking the MAT loci in all isolates. Within the MAT locus, a predicted gene with no known function was found between SLA2 and MAT1-1-4, a common feature present across all the *E. dermatitidis* isolates. The outgroup *Capronia coronata* CBS617.96 is homothallic and its genome contains both MAT1-1 and MAT1-2 genes (Teixeira *et al.* 2017).

### Chromosome copy number variation across *E. dermatitidis* isolates

**Fig. 3.**
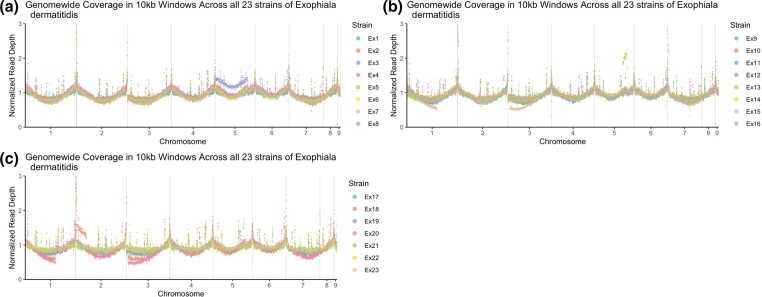
Genome sequencing depth coverage visualization to test for copy number variation across isolates. Visualization of depth of coverage was generated by plotting a normalized read depth across chromosomes for isolates a) Ex 1-8, b) Ex9-16, and c) Ex17-23.

To test for variation in partial or complete chromosome copy numbers, we calculated sequence read coverage depth from each *E. dermatitidis* isolate in 10-kb sliding windows along the nine reference genome chromosomes (Fig. 3). Visual scanning of the plots identified an anomaly of at least 1.5× higher coverage on chromosome 5 in Ex3 (Fig. 3a) encompassing about five genes or 11,600 bp (hypothetical protein—HRR75_002843, hypothetical protein—HRR75_002844, hypothetical protein—HRR75_002845, hypothetical protein—HRR75_002846, and hypothetical protein—HRR75_002847). A similar but much smaller region (2,600 bp) of chromosome 5 appears to have 2–2.25 × coverage and may be duplicated in Ex13. Additional partial 1.25–1.5 × coverage on the left arm of chromosome 2 (about 1,750 bp) in isolate Ex18 is also observed.

Some chromosome regions showed reduced coverage in the analysis. The plots of isolates Ex15, Ex18, and Ex20 indicate a normalized read depth of 0.5 × on chromosomes 1 and 3 (Fig. 3b and c), a possible sign of a segmental chromosomal aneuploidy event. Although these isolates are haploid, this reduced coverage could indicate a possible chromosome fusion of chromosomes 1 and 3. One interesting feature of these plots is the continuity in coverage patterns on chromosomes 1 and 3, which might indicate they are in fact part of the same in these isolates. Haploid organisms, like *E. dermatitidis*, could benefit from genomic plasticity through expansion or contraction to enable adaptation in a new environment (Selmecki *et al.* 2010; Legrand *et al.* 2019). Noting Ex3's potential partial aneuploidy on chromosome 5, an isolate from one of the early time points, it may be that this genome copy may contribute to an adaptive mechanism to support colonization and persistence in the human host.

**Fig. 4.**
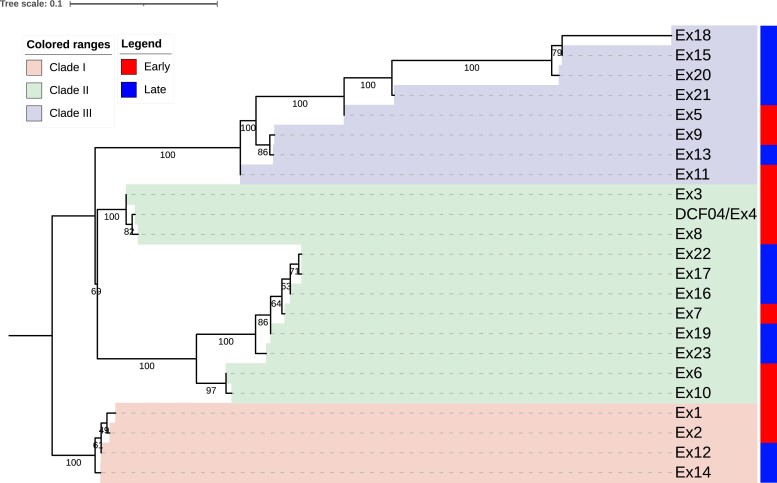
Phylogenetic tree of 23 isolates. A maximum-likelihood phylogenetic tree constructed from the single nucleotide variants by IQTREE2 identified from the isolate resequencing data. Tree is rooted with the Clade I branch based on additional analyses that included NIH/UT8656 as an outgroup. Isolates are labeled as one of three clades based on the phylogenetic relationships and the isolation time point is indicated with a red (early) or blue (late) colored box. Strains numbered from 1-11 are early collected isolates, while strains numbered from 12-23 are late isolates. Values in tree branches indicate bootstrap values calculated through iqtree2.

In addition, the genes encoded in the variable copy number region in isolate Ex3 include multiple rDNA genes. Fungi generally have larger numbers of ribosomal genes ranging from 14 to 1,442 copies (Lofgren *et al.* 2019), while these *E. dermatitidis* isolates have 22–23 rDNA copies and Ex3 has 3 complete rDNA copies in chromosome 5. Other isolates sharing potential aneuploidies (Ex15, Ex18, and Ex20) are from the late time point and also have the highest mutation rate among this population as observed from the phylogenetic tree and mutation rate calculation (Figs. 4 and 6).

**Fig. 5.**
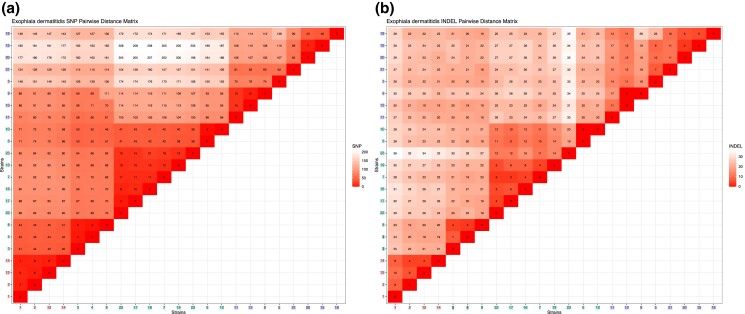
Matrix of *E. dermatitidis* CF isolates SNP and INDEL pairwise dissimilarities. The number of SNPs (a) and (b) INDELs that differs among pairs of isolates. The more similar isolate pairs have lower numbers and the number of variants increases indicating more dissimilar isolates. Clade I and Clade II isolates generally differ by very few SNP and INDELs (noting a few exceptions) consistent with their inferred near phylogenetic relationships. Within Clade III isolate pairs differ by more SNPs, which may indicate a higher mutation rate within these isolates.

**Fig. 6.**
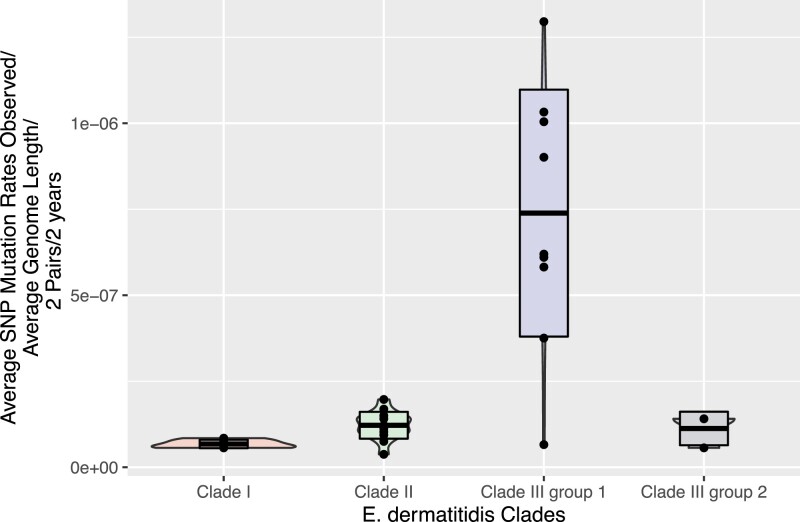
Pairwise mutation rates observed among clades. Pairwise mutation rates were calculated for all isolates in a clade and within the two subgroups of Clade III. The boxplots show median (center line in boxes) and standard deviation (box top and bottom) mutation rate for all isolate pairs in a group or clade.

### SNP genotyping and SNP-based phylogenetic analysis of 23 *E. dermatitidis* isolates

Using the DCF04/Ex4 isolate as a reference for variant identification, we identified 441 SNP variants among the 22 isolates. A phylogenetic tree was generated using these filtered SNPs, rooted with the group containing Ex1, Ex2, Ex12, and Ex14 (Fig. 4). In an additional analysis using the out-population strain NIH/UT8656 as the genome reference, we found 10,344 SNPs, with most of these being fixed differences between the outgroup and the CF isolates. The phylogenetic tree for this dataset was also generated to resolve the relative differences and root the population relationships with the NIH/UT8656 outgroup (Supplementary Figure 3).

Three clades (Clade I, Clade II, and Clade III) were identified based on the trees. Clade I is composed of two early collected (Ex1 and Ex2) and two late collected isolates (Ex12 and Ex14) of *E. dermatitidis*. Clade II is composed of three subgroups, one of which contains only early isolates and a second group with both early isolates and late isolates. A third group in Clade II contains two early collected isolates Ex6 and Ex10. Clade III has two main groups composed of both early and late isolates. The subgroup of Clade III had a long branch length suggesting more divergence. Interestingly, the CNV plot (Fig. 3) showed these three isolates, Ex15, Ex20, and Ex18 contained similar CNV differences in chromosomes 1 and 3 when compared to the other isolates.

### Nonsynonymous and synonymous SNP and INDEL pairwise differences

A dissimilarity matrix was constructed comparing the overlaps of SNPs and INDELs collated for all pairs of isolates. As expected, isolates that are closest to each other in the SNP-based phylogenetic tree had fewer differences in their SNP composition (Fig. 5a). Our analysis further confirmed consistency in mutation patterns where the number of SNP differences correlated with the number of INDELs detected in pairwise analyses (Fig. 5b). Within Clade I and Clade II, isolates had few overall variants, indicating the evolutionary distance between them was small. The total variant counts within Clade III were much higher indicating a higher divergence within this group of isolates as compared to other groups. Grouping the matrix using results from Fig. 4 provided a way to visualize incongruence, especially amongst SNPs that distinguish Ex7/Ex19 (169), and INDELs between Ex5/Ex17 (6).

### Nonsynonymous SNP differences seen between clades

Examination of predicted variant impacts and genes produced by snpEff combined with functional gene annotation provides an overview of the types of changes (Supplementary Table 6). Global comparisons between early collected Ex1–Ex11 isolates and the second time point isolates Ex12–Ex23 documented 441 variants, with a breakdown of 379 nonsynonymous to 62 synonymous mutations. Breaking down these variant numbers for all 23 isolates when compared to the reference DCF04 include 227 intergenic mutations (single point nucleotide changes), 21 frameshift mutations (affecting read count for resulting genes), 10 disruptive in-frame insertion/deletions (leading to disrupted proteins), 104 missense mutations (producing alternative amino acids), and 4 variants that fall within splice regions.

Variants generated through a gain or loss of a stop codon (nonsense mutations) were also considered in this analysis. Nine stop codon gained variants were determined, where six were described with annotated hypothetical genes, and the remaining three had descriptive annotated functions including, XPT1 xanthine phosphoribosyltransferase 1 (HRR76_002280), SIP3 SNF1-interacting protein (HRR76_004004), and ARP8 actin-like protein (HRR76_006707). The stop codon found in the XPT1 and SIP3 variants was only found in species *E. dermatitidis* Ex5, while ARP8 was found in isolates Ex16, Ex17, Ex19, Ex22, Ex23, and Ex7, which are a small branch of Clade II species further indicating these isolates may be all descendants of Ex7. One “loss of a stop codon” was determined in our variant analysis and was annotated as a hypothetical protein. Only about 10 variants resulted in a codon gained or lost in our 23 isolates of *E. dermatitidis*.

Further stratifying the genetic differences among the early and late isolates provided a more in-depth understanding of variants; ∼95 nonsynonymous to 20 synonymous early mutations were detected, while among the later isolates, there were 97 nonsynonymous and 13 synonymous segregating mutations shared among isolates as well as singleton mutations. We found that the lung environment did not generate a uniform general shift or a common variant found in all later isolates among a particular shared gene. Instead, we observed variants unequally distributed among the Clades with isolates Ex15, Ex18, Ex20, and Ex21 (Clade III) containing 58 variants. Examining the variants found only in early isolates, we counted 60 intergenic mutations, 8 frameshift mutations, 1 disruptive in-frame insertion/deletion, 21 missense mutations, and 4 variants that fall within splice regions. Among late isolates, we found 55 intergenic mutations, 5 frameshift mutations, 5 disruptive in-frame insertions/deletions, and 28 missense mutations as compared to DCF04 (an early isolate). We were expecting an overall larger number of all types of mutations would be seen in the late collected isolates, but that was not the case other than the missense mutation numbers. Otherwise, there seemed to be more variety of variants seen among the early collected isolates.

### Analysis of SNP differences between clades

A single nonsynonymous mutation was detected in a gene orthologous to *Saccharomyces cerevisiae MRS4* (HMPREF1120_06597), a mitochondrial iron transporter. One allele of the *MRS4* allele encoded a protein that was identical in sequence to the allele in the reference strain NIH/UT8656, present in the isolates in Clade I (Ex1, Ex2, Ex12, and Ex14), and a subclade of clade II (Ex3, Ex4/DCF04, and Ex8). The remainder of the isolates had a second allele with a nonsynonymous mutation in the 40th amino acid position, converting a glutamic acid residue to glycine. The functional consequences and differences of these Mrs4 alleles are described elsewhere (Murante *et al.* 2023).

### Testing for enrichment of evolutionary patterns within clades

A pairwise comparison of the synonymous and nonsynonymous SNP differences was performed on 23 pairs of isolates identified as early and late members of the same lineage. A comprehensive list of gene differences among all pairwise comparisons is in Supplementary Table 7 and includes hypothetical proteins with no identified function. This analysis tested for differences between early and late isolates found in the same clade to focus on changes that may have occurred within the host.

To better understand the pattern of variation that arose through the time points, we focused on within-clades comparison so that each early isolate within a clade was compared to the later isolate(s). Clade I is composed of four isolates: two early (Ex1 and Ex2) and two isolates from the late time point (Ex12 and Ex14). These isolates show very little genetic differentiation (about 6–9 variant SNPs and 6–10 variant INDELs). Two genes with mutations are of note, a MADS-box transcription factor (HMPREF1120_06786) and a G4 quadruplex nucleic acid binding protein (HMPREF1120_02174). The MADS-box transcription factor is part of the MADS-box proteins with a highly conserved 56 amino acid DNA-binding domain, some containing a weakly conserved K-box domain that is involved in the dimerization of transcription factors (Shore and Sharrocks 1995). In fungi, isolates with knocked-out MADS-box genes have reduced virulence (Damveld *et al.* 2005; Qu *et al.* 2014; Xiong *et al.* 2016). Mutations in *E. dermatitidis* MADS-box regions may increase their pervasiveness and tolerance of the lung environment. Previous studies have found G4 quadruplex nucleic acid binding proteins in synthetic biology applications. The helical complex is formed through guanine-rich nucleic acid sequences and is found at the telomeric regions of chromosomes. These variants are an interesting observation and warrant extra analysis in the future.

Clade II contains 11 isolates, six from the early time point and five from the later time point, which fall into three different subgroups. Ex3, Ex4/DCF04, and Ex8 fall into one group, Ex22, Ex17, Ex16, Ex7, Ex19, and Ex23 fall into the second group, and Ex6 and Ex10 form a third group. Group One and Group Three both contain only early isolates, while the second group contains the majority of the later isolates. Pairwise comparisons of the Clade II isolates contrasted the early Ex7 with each of the four late isolates Ex16, Ex17, Ex19, and Ex23. There was more variation among these isolates (about 11–169 variant SNPs and 3–16 variant INDELs) than observed in Clade I isolates. Isolate Ex23 also appeared to have substantially more differences from all others, indicating it may be more distantly related or where its corresponding ancestral isolate was not sampled. A variant of note, NAD-dependent histone deacetylase SIR2, is involved with chromosomal remodeling specifically with phenotype transcription modification (Freire-Benéitez *et al.* 2016).

Clade III, which contains eight isolates, is divided into two groups: one composed of Ex18, Ex15, Ex20, Ex21, and the other group of Ex9, Ex13, and Ex11. Five are later isolated while the other three are from the early isolations. Comparison of Clade III group 1 member Ex5 (early) to Ex15, 18, 20, and 21 identified the highest number of variants as compared to the other clades (62–138 variant SNPs and 7–10 variant INDELs). Clade III group 2 members Ex9 (early), Ex11 (early), and Ex13 had a similar number of variants as Clades I and II (6–15 variant SNPs and 0–17 variant INDELs). Four genes within the second group (Ex9, Ex13, and Ex11) were found to have distinct mutational differences when comparing Ex13 (a late isolate) to the early isolates Ex9 and Ex11. These four genes are the Ap-3 complex subunit delta (HMPREF1120_00143), DNA repair protein RAD50 (HMPREF1120_05599), a regulator of nonsense transcripts 1-like protein (HMPREF1120_06837), which is similar to helicase-RNA complex, and a gene with no identified function (HMPREF1120_02556). The second group had much more significance than the first group, consisting of three isolates (Ex18, Ex15, and Ex20) with a higher mutation rate than *E. dermatitidis* in Clade III. There are about 13 instances of hypothetical proteins, while the other 12 instances are predicted genes. Only six of the 12 are genes of note: an MFS transporter DHA2 family methylenomycin A resistance protein (HMPREF1120_00012), sulfite reductase (ferredoxin) (HMPREF1120_00943), glycerol ethanol-ferric requiring protein (HMPREF1120_01007), polyketide synthase (HMPREF1120_03173), MFS transporter SP family solute carrier family 2 (HMPREF1120_06771), and a DNA repair protein RAD50 (HMPREF1120_05599). Research on polyketide synthases in micro-colonial fungi and *E. dermatitidis* has been found to impact phenotypes and adjusts the melanin synthesis pathway, resistance or susceptibility to antifungals, and extreme environment adaptability (Paolo *et al.* 2006).

Interestingly, the group 2 (Clade III) isolates Ex9, Ex11, and Ex13 had more variants in iron-binding (HMPREF1120_06751) and siderophore-related (HMPREF1120_07838) genes than group 1. We took a candidate gene approach and tested if the siderophore NPRS SidC (HMPREF1120_07636) had accumulated any specific mutations in these lineages, but we did not identify any nonsynonymous variants in this gene across the CF lung isolates. Though, the presence of SNP variants in iron-binding and siderophore transporters indicates there may be selective pressure for *E. dermatitidis* to obtain iron in the CF lung environment, in which iron is strictly regulated. Further analysis of these variants will point to supporting phenotypic differences allowing for effective colonization of CF lung environments.

### Mutation rate calculation to test for different rates of evolution between clades

We calculated a mutation rate for each of the three clades by taking the mean and standard deviation of all the pairwise comparisons with a clade and visualized the results using a violin plot with all datapoints visible (Fig. 6). The mean Clade I mutation rate based on the four pairwise calculations for all combinations of early and late isolates was 6.76E-08. The average mutation rate for the five comparisons of early to late isolates in Clade II was 1.22E-07. The average mutation rate for the six comparisons of early to late isolates in Clade III (Group 1) values was averaged and determined to be 7.39E-07, while the second group values were averaged and determined to be 1.13E-07. The Clade III group 1 isolates appear to be a faster-evolving group and may have acquired variants allowing improved adaptation to the lung environment. One-way ANOVA test revealed a statistically significant difference between all Clades (*F*-value = 7.0711, *P*-value = 0.00104), and a Tukey post hoc test across the clades indicated Clade III (Group 1) has a significantly different mutation rate. Calculations underlying the mutation rate values are detailed in Supplementary Table 8.

### Heterogeneous phenotypes observed in the *E. dermatitidis* isolates

**Fig. 7.**
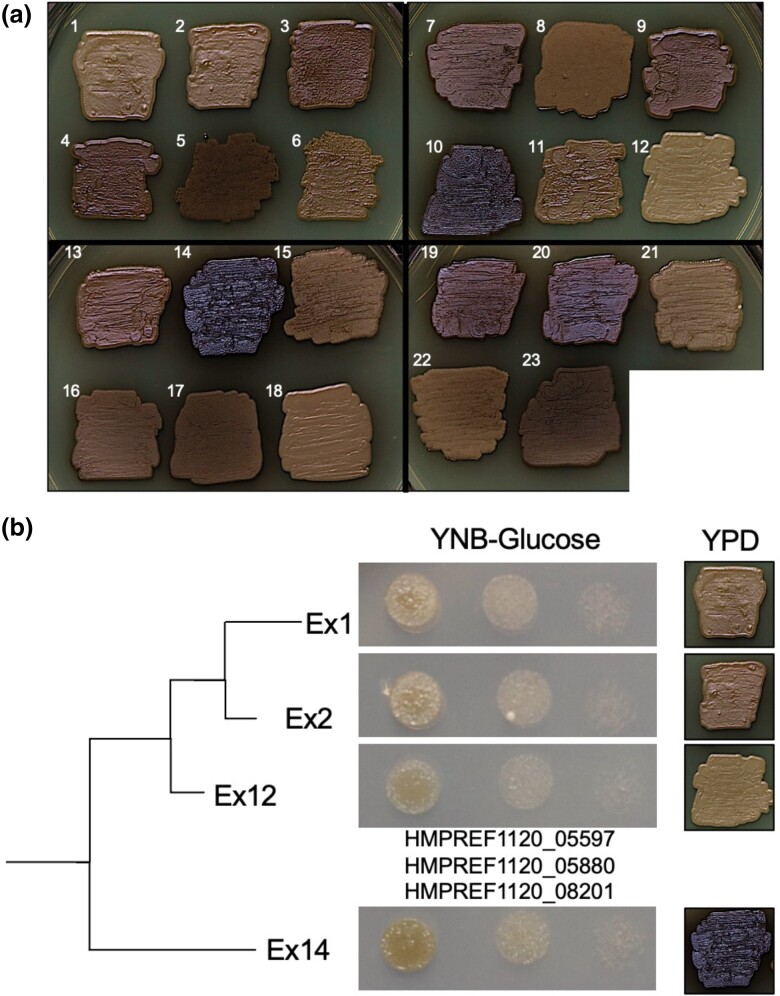
Melanin production phenotypes in the *E. dermatitidis* CF isolates. a) Melanization differences between *E. dermatitidis* CF isolates grown for 120 h on YPD at 37˚C. b) Melanization differences between closely isolates in Clade I were observed, but only a small number of SNPs differentiated low melanin producers (Ex1, Ex2, and Ex12) from a high melanin producer (Ex14). The SNPs that distinguish Ex14 from the three low production isolates are in the upstream intergenic regions of three genes: a monoamine oxidase HRR76_005273; a putative biphenyl-2,3-dioxygenase, HRR76_005538, and a gene of no known function, HRR6_007729. The three loci identifiers correspond to HMPREF1120_05597, HMPREF1120_05880, and HMPREF1120_08201 respectively.

The 23 *E. dermatitidis* isolates were heterogeneous for traits in several ways including melanization, antifungal sensitivity, and auxotrophy. We noted differences in melanin pigmentation across isolates (Fig. 7a) that did not correlate with either time point or phylogenetic clade. Within Clade 1, Ex1, Ex2, and Ex12 had light melanization, but isolate Ex14 was one of the strongest melanin producers (Fig. 7b). In a search for genetic determinants responsible for this phenotypic range, we identified three candidate intergenic mutations, which differentiated Ex14 from the other members of the clade.

**Fig. 8.**
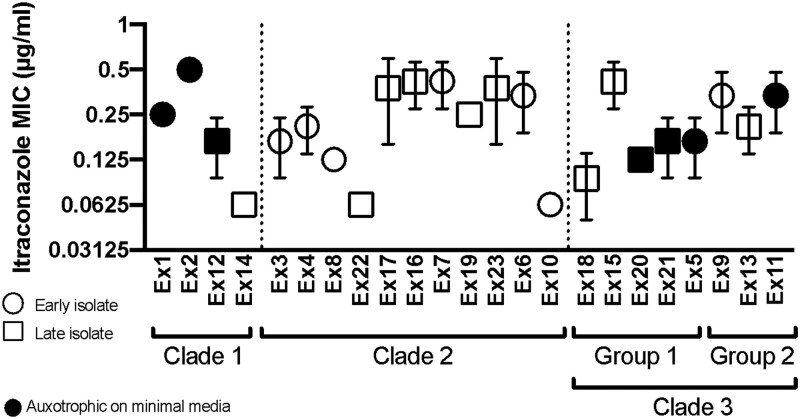
Itraconazole sensitivity testing of 23 CF isolates. Average minimum inhibitory concentration (MIC) of CF isolates grown with Itraconazole. Standard deviation of three biological replicates is indicated by error bars. The auxotrophies of some isolates, indicated by filled symbols, was identified by growth on minimal medium (YNB) without casamino acids. Symbol shape indicates whether isolates were recovered from the early or late sputum samples.

**Fig. 9.**
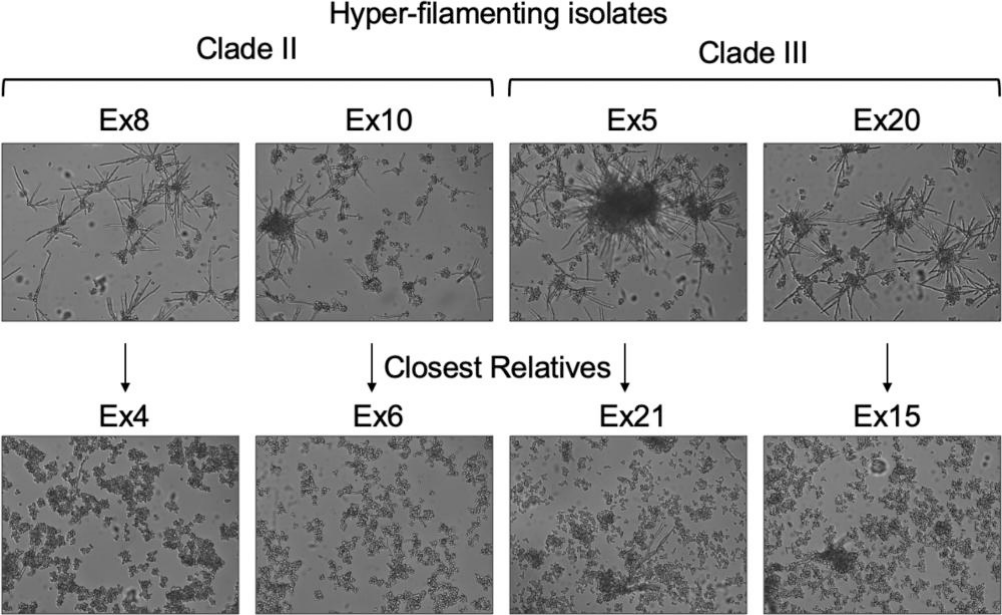
Cell morphology by microscopy. A hyper-filamentous cell morphology was found among members of two of the clades (top row). For each of these isolates, closely related isolates from the same clade grew in the typical yeast-like cellular morphology (second row). All isolates were grown in RPMI-1640 medium for 18 h at 37˚C in a 3-ml culture 6-well dish format. Culture aliquots were imaged with a Zeiss Axiovert 200M inverted microscope.

The 23 isolates also varied in sensitivity to itraconazole, a recommended treatment for *E. dermatitidis* infections (Fothergill *et al.* 2009; Mukai *et al.* 2014), over an ∼10-fold range (MIC from 0.0625 to 0.5 µg/ml) (Fig. 8). Heterogeneity in amino acid auxotrophy, scored as no growth on a minimal medium that was rescued by supplementation of amino acids (Fig. 8), was also observed. Lastly, there were stable differences in filamentation across isolates in two of the three clades; closely related isolates did not have similar morphologies (Fig. 9).

## Discussion

### Fungus-dominant population

While opportunistic fungal pathogens are often culturable from the sputum of patients with CF, they do not commonly present as the dominant microbe or as a risk for infection. In this paper, we have presented a clinical case where a population of the black yeast *E. dermatitidis* was the predominant microbe concomitant with a lung exacerbation event. Other reported clinical cases have shown that *E. dermatitidis* has an underappreciated role as a CF pathogen (Haase *et al.* 1990; Kusenbach *et al.* 1992; Kondori *et al.* 2014). Previous sputum isolates revealed the presence of these uncommon fungi 2 years prior, indicating that it persisted in low concentrations before having the opportunity to dominate the lung microbiome. Because *E. dermatitidis* is relatively slow-growing (Rath *et al.* 1997; Sudhadham *et al.* 2011; Malo *et al.* 2021), it may be easily missed during routine clinical microbiological identification. To better understand the CF disease and the fungal infections associated with a long-term disease such as this one, it is important to consider testing for slow-growing microorganisms such as black yeasts.

### Phenotypic heterogeneity, melanin production, amino acid metabolism, filamentation, and drug resistance

Developing methods for evaluating population structure and heterogeneity in infecting microbes will aid future studies. Initial observations of these isolates indicated a striking amount of heterogeneity in melanin production, which propelled an investigation into the genotypic diversity of the population. Melanin production in fungi is associated with increased survival in stress conditions, virulence, dissemination, and host immune response (Eisenman and Casadevall 2012). *Exophiala dermatitidis* produce DHN-melanin from 1,8 dihydroxynaphthalene polymerization, the more common and better-characterized pathway in fungi, compared to L-DOPA, produced DOPA-melanin, or pyomelanin produced from the degradation of tyrosine (Jia *et al.* 2021). DHN synthesis initiates from endogenous acetyl CoA or melanyl CoA, which is converted to 1,3,6,8 tetrahydroxynaphthalene by polyketide synthase, and converted to DHN-melanin through a series of reductions and polymerizations. While we observed stable differences in melanin production in closely related isolates with few mutations differentiating them, future experiments are required to determine whether these SNPs play a role in melanin synthesis, cell wall localization, or other melanin-associated biological processes. While melanin, which comprises part of the cell wall in black yeasts, can influence antifungal resistance, there were no obvious differences in sensitivity to Itraconazole and Voriconazole that correlated with melanin production (Fig. 8). Other characteristics, such as growth rate or auxotrophy also showed no correlation with levels of melanization.

The discovery of amino acid auxotrophs in the population was consistent with reports of auxotrophs among bacterial CF isolates and likely reflects the presence of amino acids in the lumen of the CF lung (Barth and Pitt 1995; Thomas *et al.* 2000). Auxotrophy was not monophyletic, and neither was growth as filaments in RPMI with 5% CO2. The filamentation phenotype in phylogenetically diverse isolates may indicate a nonmutation-based difference between isolates such as “switching” or phase variation, epigenetic regulation, or strong repeated selection for the phenotype.

### Siderophore gene mutations and iron acquisition

Successful microbial invasions require iron, a critical growth-limiting factor, which must be sequestered from the surrounding environment through the release of iron-chelating proteins called siderophores (Neilands 1995; Mossialos and Amoutzias 2009). For example, *P. aeruginosa* produces the fluorescent siderophores, pyoverdine, and pyochelin, which are used to sequester iron from the lung environment and as cofactors for respiratory proteins needed for surface motility and biofilm maturation (Haase *et al.* 1990; Banin *et al.* 2005; Matilla *et al.* 2007). Another example, the fungus *Aspergillus fumigatus* produces two hydroxamate-type siderophores: triacetylfusarinine (SidD) and ferricrocin (SidC), while also producing fusarinine and hydroxyferricrocin type siderophores (Schrettl *et al.* 2007; Haas 2014). A study by Zajc *et al.* (2019) indicated 10 predicted siderophores in four *E. dermatitidis* genomes, two of which include triacetylfusarinine and ferricrocin. Ferricrocin is an internal siderophore used to store iron and is essential for sexual development and contributes to oxidative stress resistance (Schrettl *et al.* 2007; Tyrrell and Callaghan 2016). Triacetylfusarinine is used to facilitate hyphal growth under iron-depleted conditions (Schrettl *et al.* 2007; Tyrrell and Callaghan 2016).

The *E. dermatitis* locus (HMPREF1120_07636) encodes the nonribosomal peptide synthase SidC necessary for siderophore synthesis (Zajc *et al.* 2019; Malo *et al.* 2021). Polymicrobial infections persist in the CF lung environment through the production and scavenging of extracellular siderophores, which aid microbes in competition for resources (Tyrrell and Callaghan 2016; Sass *et al.* 2019; Yan *et al.* 2022). Microbes can use, obtain, and sequester iron in siderophores from hemoglobin found in red blood cells and lactoferrin contained in mucosal secretions. The competition and use of iron is an important dynamic in polymicrobial infections including fungal-bacteria competition within plant and animal hosts, and may sometimes assist in promoting the growth of their host (Crowley *et al.* 1991; Johnson 2008; Aznar *et al.* 2014; Kim 2018; Mochochoko *et al.* 2021; Pohl Carolina and Noverr Mairi 2022). As these isolates were collected from sputum plates, it could be certain isolates have co-localized within different lobes of the lung and could have nutritional or other ecological partitioning that could be driving these clades to diverge and remain diverged. Our results suggest that there may have been a diversification event that occurred early perhaps during the initial colonization. The clades then stabilized once co-localizing into their respective niches, or there may have been a second “inoculation” event from a common, stably heterogeneous environmental source at some point in the 2-year timeline (Warren *et al.* 2011).

### Short- and long-read sequencing, in-population vs outside reference

While short-read sequencing provided us with the basic and high-quality read depth for each CF isolate, using the ONT long-read technology sequencing to improve short-read assembly provided us with a well-assembled genome. This genome was then used as an in-population reference allowing for a better recovery of variants due to the read mapping to a closer isolate. When using our in-population reference, compared to the NIH/UT8656 strain (Robertson *et al.* 2012; Chen *et al.* 2014; Schultzhaus *et al.* 2020; Malo *et al.* 2021), our quantification of population-specific number of variants is higher (due to higher sensitivity) while still maintaining relevance in our study system.

### Temporal resolution of variation accumulation in a fungal CF lung population

Contrasting mutations accumulating in isolates collected from two time points allowed comparison in mutation rate differences among three different genetic lineages of *E. dermatitidis* and evaluated if any changes could suggest adaptations that enabled lineages to survive the lung environment. Average mutation rates within a Clade tended to be similar indicating common diversification events. Statistical comparisons of mutation rates between Clades indicate significance seen between Clade I and Clade III Group 1, Clade II and Clade III Group 1, and Clade III Group 1 and Group 2. The main difference here is the greatly increased diversification in Clade III group 1, which is shown with the phylogenetic tree in Fig. 4, chromosomal aneuploidies seen in Fig. 3, the very high SNP and INDEL counts seen in Fig. 5, the increased mutation rates seen in Fig. 6, and hyper-filamentous phenotypes see in Fig. 8. We propose the mutations found in *RAD50* may have contributed to the increased diversification seen in this group and future studies will test this hypothesis (Goldman *et al.* 2002; Krogh and Symington 2004).

These clades persisted over a 2-year span. Are there physical separations preventing these sub-populations to mix? When observing the functional assessment of variants unique to each clade, we identified alleles in genes that encode for transporters, cytochrome P450 oxidoreductases, iron acquisition, and DNA repair processes. The iron acquisition can be a possible virulence method *E. dermatitidis* could be used to persist in the lung environment as seen in *Aspergillus fumigatus* and *P. aeruginosa* (Neilands 1995; Matilla *et al.* 2007; Schrettl *et al.* 2007). Mutations in transporters could be an evolutionary move to adapt to antibiotics or antifungal treatments. The finding of a nonsynonymous mutation, *MRS3/4* a mitochondrial iron transporter, may also help point to the pathogenicity and virulence (Murante *et al.* 2023). Though, the presence of certain SNP variants in iron-binding and siderophore transporters suggests that there could be other means by which *E. dermatitidis* is obtaining the iron molecules from its environment. Further analysis of these variants will point to supporting phenotypic differences allowing for effective colonization of CF lung environments. It is clear that this mutation is not found in the rapidly diverging Clade III and only in the Clade I cluster with very few variants. The isolates we have sequenced only contain one mating type, and no mating was detected as all isolates were haploid, thus indicating the low likelihood that even given the opportunity by proximity, they are unable to mate.

## Conclusions

FAs it has become increasingly clear, collecting a single isolate and using it as a metric to assess an environment or moment in time is likely to misrepresent the population; a population-level approach will likely provide more insight into microbes present in infections (Demers *et al.* 2018). Population-level studies greatly benefit from having a closely related reference strain for the purposes of assessing variants. Our results indicate the CF lung environment supports stably diverged populations of clonally derived yeasts.

## Supplementary Material

jkad126_Supplementary_Data

## Data Availability

Sequence data generated for isolates with Illumina and Oxford Nanopore technology are deposited in NCBI Sequence Read Archive linked under BioProject PRJNA628510. The assembled genomes of each CF isolate (Ex1-23) are available under accessions listed in Supplementary Table 2. The assembled and annotated genome of the in-population reference isolate (DCF04) is available at accession JAJGCF000000000. All analysis pipelines, custom scripts used for data analysis, and raw variant data in the variant call format are available in the Github repository https://github.com/tania-k/CF_Exophiala_dermatitidis and archived in Zenodo (Kurbessoian 2022). Supplemental material available at G3 online.
