# Supplementary material for: In host evolution of Exophiala dermatitidis in cystic fibrosis lung micro-environment
Source: G3 (Bethesda). 2023 Jun 9;13(8):jkad126. doi: 10.1093/g3journal/jkad126 (PMC10484061; doi:10.1093/g3journal/jkad126)
Supplement: jkad126_Supplementary_Data [file jkad126_supplementary_data.zip › Supplemental_Material_Legends_G3-2023-404223.docx]

**Supplemental Figure 1. Dot plot displaying similarity and collinearity of *E. dermatitidis* DCF04 and NIH/UT8656 genomes.** Genome scale dot plot representation of genome similarity between genomes of *E. dermatitidis* DCF04 and NIH/UT8656 was created with D-GENIES web application. High similarity and lack of structural changes or rearrangements is apparent by the complete collinearity of the two genomes represented by the straight diagonal line.

**Supplemental Figure 2. Mating-type determination of 23 clinical isolates of *E. dermatitidis.*** Gene content, order and orientation of the MAT locus from 23 CF isolated *E. dermatitidis*, NIH/UT8656 strain and the Chaetothyriales black yeast *Capronia coronata* CBS 617.98. The locus is flanked by two genes SLA2 (purple) and APN2 (green). The MAT 1-1 genes, MAT 1-1-4 (orange) and MAT 1-1-1 (yellow) are observed in all 23 CF strains while the reference strain NIH/UT8656 has a MAT 1-2 (teal) gene. In addition, two genes are predicted in the interval between SLA2 and the MAT genes. One is a L-type calcium channel domain (blue), which is only found in the *E. dermatitidis* isolates, and a gene with no identified function or domains (pink) which is syntentically adjacent to the MAT genes in only some isolates.

**Supplemental Figure 3. Phylogenetic tree of 24 isolates.** A Maximum-Likelihood phylogenetic tree constructed from the Single Nucleotide Variants by IQTREE2 identified from the isolate resequencing data. Tree is rooted with NIH/UT8656 as an outgroup. Isolates are labeled as one of three clades based on the phylogenetic relationships and the isolation time point is indicated with a red (early) or blue (late) colored box. Values in tree branches indicate bootstrap values calculated through iqtree2.

**Supplemental Table 1. Collection and MIC values for CF patient derived *E. dermatitidis* isolates.** Information on the 23 CF isolates cultured from one patient sputum across three years. The table summarizes phylogenetic clade designation, itraconazole MIC, date of collection, and classification as an Early or Late.

**Supplemental Table 2. Genome assembly statistics for 23 CF isolates**. Assembly statistics for scaffolded assemblies of isolates and NIH/UT8656 previously published genome. Table indicates assembly summary statistics of the assembly using Illumina sequencing of all 23 CF isolates. Summary statistics of scaffold lengths are presented with genome completeness data calculated with BUSCO using the ascomycota_odb9 database.

**Supplemental Table 3. Telomere Recovery for DCF04.** Table showing telomere recovery for DCF04 *E. dermatitidis*. The candidate telomeric repeat units “TTTAGGG/CCCTAA” were identified as arrays of repeats at both ends of five of the scaffolds, and as repeats on only one end of the remaining 3 scaffolds.

**Supplemental Table 4.** **OrthoFinder summary comparing DCF04 and NIH/UT8656.** Comparison of the shared and unique orthogroups found in the annotated proteomes of *E. dermatitidis* strains NIH/UT8656 and DCF04. A majority of orthogroups (8,256; 99%) had members from both strains, of these 15 were single-copy orthogroups containing a single protein-coding gene from each strain. There were 10 orthogroups unique to DCF04 encompassing 34 protein-coding genes and 5 orthogroups unique to NIH/UT8656 made up of 24 protein-coding genes. DCF04 contained 705 unassigned genes, while NIH/UT8656 had 475.

**Supplemental Table 5. Mating-type determination locus name descriptions of *E. dermatitidis*.** This table describes all the proteins depicted presented in **Figure 2** of the gene content, order and orientation of MAT locus from *E. dermatitidis* isolates in this study, strain NIH/UT8656 along with the Chaetothyriales black yeast *Capronia coronata* CBS 617.98.

**Supplemental Table 6. Functional impact of identified variants.** Filtered human readable snpEff tabular results for all CF 23 *E. dermatitidis*. Annotations have been added to the final list to better help assess the function of each protein while also observing variants detected from GATK.

**Supplemental Table 7. All functional SNP and INDEL results for early and late pairs.** Results indicated in this table include all hypothetical or undescribed results. Gene locus names listed include *Exophiala dermatitidis* UT/8656 Protein ID numbers to better facilitate identification and cross-reference.

**Supplemental Table 8. All 23 mutation rates were calculated.** Mutation rates for each 23 CF *E. dermatitidis* isolates calculated using formula described in methods. One-way ANOVA was run to detect significance (p-value= 0.00104, F-value = 7.0711) along with Tukey multiple comparisons of means indicating Clade III had the highest significance among the six pairwise comparisons.
