# Supplementary material for: In host evolution of Exophiala dermatitidis in cystic fibrosis lung micro-environment
Source: G3 (Bethesda). 2023 Jun 9;13(8):jkad126. doi: 10.1093/g3journal/jkad126 (PMC10484061; doi:10.1093/g3journal/jkad126)
Supplement: jkad126_Supplementary_Data [file jkad126_supplementary_data.zip › Supplemental_Table_1_G3-2023-404223.docx]

**Supplemental Table 1. Collection and MIC values for CF patient derived *E. dermatitidis* isolates**

| Strain ID | Clade Node | MIC ng/ml | Year Isolated | Early or Late |
| --- | --- | --- | --- | --- |
| Ex2 | I | 500 | 2014 | Early |
| Ex1 | I | 250 | 2014 | Early |
| Ex12 | I | 250 | 2016 | Late |
| Ex14 | I | 62.5 | 2016 | Late |
| Ex6 | II | 250 | 2014 | Early |
| Ex10 | II | 62.5 | 2014 | Early |
| Ex22 | II | 62.5 | 2016 | Late |
| Ex17 | II | 250 | 2016 | Late |
| Ex16 | II | 500 | 2016 | Late |
| Ex7 | II | 500 | 2014 | Early |
| Ex19 | II | 250 | 2016 | Late |
| Ex23 | II | 250 | 2016 | Late |
| Ex3 | II | 125 | 2014 | Early |
| Ex8 | II | 125 | 2014 | Early |
| Ex4/DCF04 | II | 250 | 2014 | Early |
| Ex15 | III | 250 | 2016 | Late |
| Ex18 | III | 62.5 | 2016 | Late |
| Ex20 | III | 125 | 2016 | Late |
| Ex21 | III | 125 | 2016 | Late |
| Ex5 | III | 250 | 2014 | Early |
| Ex9 | III | 500 | 2014 | Early |
| Ex13 | III | 250 | 2016 | Late |
| Ex11 | III | 250 | 2014 | Early |
