# Supplementary material for: In host evolution of Exophiala dermatitidis in cystic fibrosis lung micro-environment
Source: G3 (Bethesda). 2023 Jun 9;13(8):jkad126. doi: 10.1093/g3journal/jkad126 (PMC10484061; doi:10.1093/g3journal/jkad126)
Supplement: jkad126_Supplementary_Data [file jkad126_supplementary_data.zip › Supplemental_Table_4_G3-2023-404223.docx]

**Supplemental Table 4.** **OrthoFinder summary comparing DCF04 and NIH/UT8656.**

| **OrthoFinder Results** | ***E. dermatitidis* DCF04** | ***E. dermatitidis* NIH/UT8656** |
| --- | --- | --- |
| Number of genes | 9535 | 9285 |
| Number of genes in orthogroups | 8830 | 8810 |
| Number of unassigned genes (species-specific) | 705 | 475 |
| Number of genes in species-specific orthogroups | 34 | 24 |
| Total number of genes specific to each species | 739 | 499 |

| **Summary Statistics** |  |
| --- | --- |
| Number of genes | 18,820 |
| Number of genes in orthogroups | 17,640 |
| Number of unassigned genes | 1,180 |
| Number of orthogroups | 8,271 |
| Number of orthogroups with both taxa present | 8,256 |
| Number of single-copy orthogroups | 7,818 |

**Lineage-specific paralogs**

| **Orthogroup** | **DCF04** | **NIH/UT8656** | **Total** | **Function described by InterProScan** | **Function described by UniProt** |
| --- | --- | --- | --- | --- | --- |
| OG0000035 | 0 | 8 | 8 | [IPR034001](https://www.ebi.ac.uk/interpro/entry/InterPro/IPR034001/),[IPR013525](https://www.ebi.ac.uk/interpro/entry/InterPro/IPR013525/),[PF01061](https://www.ebi.ac.uk/interpro/entry/pfam/PF01061/),[IPR010929](https://www.ebi.ac.uk/interpro/entry/InterPro/IPR010929/),[PF06422](https://www.ebi.ac.uk/interpro/entry/pfam/PF06422/)[,IPR043926](https://www.ebi.ac.uk/interpro/entry/InterPro/IPR043926/),[PF19055](https://www.ebi.ac.uk/interpro/entry/pfam/PF19055/),[IPR003593](https://www.ebi.ac.uk/interpro/entry/InterPro/IPR003593/)[,IPR003439](https://www.ebi.ac.uk/interpro/entry/InterPro/IPR003439/),[PF00005](https://www.ebi.ac.uk/interpro/entry/pfam/PF00005/)[,IPR029481](https://www.ebi.ac.uk/interpro/entry/InterPro/IPR029481/),[PF14510](https://www.ebi.ac.uk/interpro/entry/pfam/PF14510/),[IPR034003](https://www.ebi.ac.uk/interpro/entry/InterPro/IPR034003/) | [ABC multidrug transporter (H6BMG9_EXODN) - HMPREF1120_00270](https://www.uniprot.org/uniprotkb/H6BMG9/entry) |
| OG0000038 | 0 | 7 | 7 | [IPR004841](https://www.ebi.ac.uk/interpro/entry/InterPro/IPR004841/), [PF00324](https://www.ebi.ac.uk/interpro/entry/pfam/PF00324/), [IPR004840](https://www.ebi.ac.uk/interpro/entry/InterPro/IPR004840/), [PS00218](https://www.ebi.ac.uk/interpro/entry/prosite/PS00218/) | [AAT family amino acid transporter (H6BKY0_EXODN) - HMPREF1120_00008](https://www.uniprot.org/uniprotkb/H6BKY0/entry) |
| OG0000108 | 0 | 5 | 5 | [IPR045079](https://www.ebi.ac.uk/interpro//entry/interpro/IPR045079), [IPR045079](https://www.ebi.ac.uk/interpro/entry/InterPro/IPR045079/), [IPR003692](https://www.ebi.ac.uk/interpro/entry/InterPro/IPR003692/), [IPR002821](https://www.ebi.ac.uk/interpro/entry/InterPro/IPR002821/), [IPR008040](https://www.ebi.ac.uk/interpro/entry/InterPro/IPR008040/), [PF19278](https://www.ebi.ac.uk/interpro/entry/pfam/PF19278/) | [5-oxoprolinase (ATP-hydrolysing) (H6BU32_EXODN) -HMPREF1120_03739](https://www.uniprot.org/uniprotkb/H6BU32/entry) |
| OG0008269 | 0 | 2 | 2 | [IPR004217](https://www.ebi.ac.uk/interpro/entry/InterPro/IPR004217/), [IPR035427](https://www.ebi.ac.uk/interpro/entry/InterPro/IPR035427/), | [Mitochondrial import inner membrane translocase subunit (H6C246_EXODN) - HMPREF1120_06682](https://www.uniprot.org/uniprotkb/H6C246/entry) |
| OG0008270 | 0 | 2 | 2 | none detected | [DUF300-domain-containing protein (H6C2Q5_EXODN) - HMPREF1120_05993](https://www.uniprot.org/uniprotkb/H6C2Q5/entry) |
|  |  |  |  |  |  |
| OG0000020 | 8 | 0 | 8 | [IPR043926](https://www.ebi.ac.uk/interpro/entry/InterPro/IPR043926/), [PF19055](https://www.ebi.ac.uk/interpro/entry/pfam/PF19055/), [IPR003439](https://www.ebi.ac.uk/interpro/entry/InterPro/IPR003439/), [PF00005](https://www.ebi.ac.uk/interpro/entry/pfam/PF00005/), [IPR034001](https://www.ebi.ac.uk/interpro/entry/InterPro/IPR034001/), [IPR034003](https://www.ebi.ac.uk/interpro/entry/InterPro/IPR034003/), [IPR003593](https://www.ebi.ac.uk/interpro/entry/InterPro/IPR003593/), [IPR010929](https://www.ebi.ac.uk/interpro/entry/InterPro/IPR010929/), [PF06422](https://www.ebi.ac.uk/interpro/entry/pfam/PF06422/), [IPR013525](https://www.ebi.ac.uk/interpro/entry/InterPro/IPR013525/), [PF01061](https://www.ebi.ac.uk/interpro/entry/pfam/PF01061/), [IPR027417](https://www.ebi.ac.uk/interpro/entry/InterPro/IPR027417/), [IPR017871](https://www.ebi.ac.uk/interpro/entry/InterPro/IPR017871/) | [ABC multidrug transporter (H6BUG2_EXODN) - HMPREF1120_03829](https://www.uniprot.org/uniprotkb/H6BUG2/entry) |
| OG0000043 | 6 | 0 | 6 | [IPR004841](https://www.ebi.ac.uk/interpro/entry/InterPro/IPR004841/), [PF00324](https://www.ebi.ac.uk/interpro/entry/pfam/PF00324/) | [AAT family amino acid transporter (H6BS85_EXODN) - HMPREF1120_02315](https://www.uniprot.org/uniprotkb/H6BS85/entry) |
| OG0000101 | 5 | 0 | 5 | [IPR045079](https://www.ebi.ac.uk/interpro/entry/InterPro/IPR045079/), [IPR003692](https://www.ebi.ac.uk/interpro/entry/InterPro/IPR003692/), [PF02538](https://www.ebi.ac.uk/interpro/entry/pfam/PF02538/), [IPR002821](https://www.ebi.ac.uk/interpro/entry/InterPro/IPR002821/), [PF01968](https://www.ebi.ac.uk/interpro/entry/pfam/PF01968/), [IPR008040](https://www.ebi.ac.uk/interpro/entry/InterPro/IPR008040/), [PF05378](https://www.ebi.ac.uk/interpro/entry/pfam/PF05378/), [PF19278](https://www.ebi.ac.uk/interpro/entry/pfam/PF19278/) | [5-oxoprolinase (ATP-hydrolysing) (H6BU32_EXODN) - HMPREF1120_03739](https://www.uniprot.org/uniprotkb/H6BU32/entry) |
| OG0000376 | 8 | 0 | 3 | none detected | [Uncharacterized protein (W9WQ17_9EURO) - A1O5_09736](https://www.uniprot.org/uniprotkb/W9WQ17/entry) |
| OG0001396 | 6 | 0 | 2 | none detected | [Uncharacterized protein (W9WQZ1_9EURO) - A1O5_09737](https://www.uniprot.org/uniprotkb/W9WQZ1/entry) |
| OG0002424 | 5 | 0 | 2 | [IPR008775](https://www.ebi.ac.uk/interpro/entry/InterPro/IPR008775/), [PF05721](https://www.ebi.ac.uk/interpro/entry/pfam/PF05721/) | [Phytanoyl-CoA dioxygenase (W2S4I4_9EURO) - HMPREF1541_02111](https://www.uniprot.org/uniprotkb/W2S4I4/entry) |
| OG0003707 | 3 | 0 | 2 | [IPR018357](https://www.ebi.ac.uk/interpro/entry/InterPro/IPR018357/) | [Uncharacterized protein (A0A834JIH9_VESGE) - HZH68_013233](https://www.uniprot.org/uniprotkb/A0A834JIH9/entry) |
| OG0004734 | 2 | 0 | 2 | [IPR002156](https://www.ebi.ac.uk/interpro/entry/InterPro/IPR002156/), [PF00075](https://www.ebi.ac.uk/interpro/entry/pfam/PF00075/), [IPR011320](https://www.ebi.ac.uk/interpro/entry/InterPro/IPR011320/), [PF01693](https://www.ebi.ac.uk/interpro/entry/pfam/PF01693/), [IPR009027](https://www.ebi.ac.uk/interpro/entry/InterPro/IPR009027/), [IPR036397](https://www.ebi.ac.uk/interpro/entry/InterPro/IPR036397/), [IPR037056](https://www.ebi.ac.uk/interpro/entry/InterPro/IPR037056/), [IPR012337](https://www.ebi.ac.uk/interpro/entry/InterPro/IPR012337/) | [Ribonuclease HI (W9XHR7_9EURO) - A1O3_08353](https://www.uniprot.org/uniprotkb/W9XHR7/entry) |
| OG0004821 | 2 | 0 | 2 | [IPR002641](https://www.ebi.ac.uk/interpro/entry/InterPro/IPR002641/), [PF01734](https://www.ebi.ac.uk/interpro/entry/pfam/PF01734/), [IPR016035](https://www.ebi.ac.uk/interpro/entry/InterPro/IPR016035/) | [PNPLA domain-containing protein (H6C3A7_EXODN) - HMPREF1120_06140](https://www.uniprot.org/uniprotkb/H6C3A7/entry) |
| OG0006001 | 2 | 0 | 2 | none detected | [Uncharacterized protein (A0A0D2DN12_9EURO) - Z517_08901](https://www.uniprot.org/uniprotkb/A0A0D2DN12/entry) |

**Lineage-specific unassigned paralogs**

Full list can be found in Zenodo as OrthoFinder/Orthogroups_UnassignedGenes.tsv

doi: [10.5281/zenodo.7106110](https://doi.org/10.5281/zenodo.7106110)

**OrthoFinder lineage-specific singleton genes but found in respective genomes, indicating incomplete annotation or mis-assembly.**

| **Orthogroup** | **DCF04/*E.dermatitidis* strains** | **NIH/UT8656** | **Function described by InterProScan** | **Function described by UniProt** |
| --- | --- | --- | --- | --- |
| OG0008348 | 0 *E. dermatitidis* strains | 1 | None detected | [uncharacterized protein (A0A8H8U6S1_9HELO)](https://www.uniprot.org/uniprotkb/A0A8H8U6S1/entry) |
| OG0008357 | 0 *E. dermatitidis* strains | 1 | None detected | [Putative type II secretion system protein F (A0A5C6CDG5_9BACT) - gspF_2](https://www.uniprot.org/uniprotkb/A0A5C6CDG5/entry) |
| OG0008368 | 0 *E. dermatitidis* strains | 1 | None detected | [uncharacterized protein (W9YKF8_9EURO)](https://www.uniprot.org/uniprotkb/W9YKF8/entry) |
| OG0008377 | 0 *E. dermatitidis* strains | 1 | None detected | [3'-5' exonuclease domain-containing protein (A0A1B8A849_FUSPO)](https://www.uniprot.org/uniprotkb/A0A1B8A849/entry) |
| OG0008396 | 0 *E. dermatitidis* strains | 1 | None detected | [uncharacterized protein (W9WQY5_9EURO)](https://www.uniprot.org/uniprotkb/W9WQY5/entry) |
| OG0008499 | 0 *E. dermatitidis* strains | 1 | None detected | [protein TsetseEP-like (A0A1S3WWH5_ERIEU) - LOC103128294](https://www.uniprot.org/uniprotkb/A0A1S3WWH5/entry) |
| OG0008501 | 0 *E. dermatitidis* strains | 1 | None detected | [Fibronectin type-III domain-containing protein (A0A7M3RKJ1_SPIER)](https://www.uniprot.org/uniprotkb/A0A7M3RKJ1/entry) |
| OG0008507 | 0 *E. dermatitidis* strains | 1 | None detected | [Cell wall-associated NlpC family hydrolase (A0A4V3CYZ5_LABRH)](https://www.uniprot.org/uniprotkb/A0A4V3CYZ5/entry) |
| OG0008558 | 0 *E. dermatitidis* strains | 1 | [PF07732](http://www.ebi.ac.uk/interpro/entry/pfam/PF07732/taxonomy/uniprot/), [PF07731](https://www.ebi.ac.uk/interpro/entry/pfam/PF07731/) | [Plastocyanin-like domain-containing protein (A0A0D2EW42_9EURO)](https://www.uniprot.org/uniprotkb/A0A0D2EW42/entry) |
| OG0008660 | 0 *E. dermatitidis* strains | 1 | None detected | [uncharacterized protein (A0A3M7NCM5_9EURO)](https://www.uniprot.org/uniprotkb/A0A3M7NCM5/entry) |
| OG0008767 | 0 *E. dermatitidis* strains | 1 | [PF14342](https://www.ebi.ac.uk/interpro/entry/pfam/PF00234/) | [DUF4396 domain-containing protein (A0A0D2D007_9EURO)](https://www.uniprot.org/uniprotkb/A0A0D2D007/entry) |
| OG0008772 | 0 *E. dermatitidis* strains | 1 | None detected | [Alpha box domain-containing protein (W9YJ08_9EURO)](https://www.uniprot.org/uniprotkb/W9YJ08/entry) |
| OG0008807 | 0 *E. dermatitidis* strains | 1 | None detected | [IGS10 protein (A0A7K9P8N5_9CORV) - Igsf10_1](https://www.uniprot.org/uniprotkb/A0A7K9P8N5/entry) |
| OG0008810 | 0 *E. dermatitidis* strains | 1 | None detected | [uncharacterized protein (U7PN57_SPOS1)](https://www.uniprot.org/uniprotkb/U7PN57/entry) |
| OG0008837 | 0 *E. dermatitidis* strains | 1 | None detected | [uncharacterized protein (A0A0D2G190_9EURO)](https://www.uniprot.org/uniprotkb/A0A0D2G190/entry) |
| OG0008848 | 0 *E. dermatitidis* strains | 1 | [PF13577](https://www.ebi.ac.uk/interpro/entry/pfam/PF13577/) | [SnoaL-like domain-containing protein (W2S2Y8_9EURO)](https://www.uniprot.org/uniprotkb/W2S2Y8/entry) |
| OG0008857 | 0 *E. dermatitidis* strains | 1 | None detected | [uncharacterized protein (A0A291LXG5_9RHOB)](https://www.uniprot.org/uniprotkb/A0A291LXG5/entry) |
| OG0008864 | 0 *E. dermatitidis* strains | 1 | None detected | [uncharacterized protein (W9WGP0_9EURO)](https://www.uniprot.org/uniprotkb/W9WGP0/entry) |
| OG0008865 | 0 *E. dermatitidis* strains | 1 | None detected | [HTH CENPB-type domain-containing protein (W9XY18_9EURO)](https://www.uniprot.org/uniprotkb/W9XY18/entry) |
| OG0008940 | 0 *E. dermatitidis* strains | 1 | None detected | [uncharacterized protein (A0A072PK57_9EURO)](https://www.uniprot.org/uniprotkb/A0A072PK57/entry) |
| OG0008952 | 0 *E. dermatitidis* strains | 1 | None detected | [uncharacterized protein (W9X9G6_9EURO)](https://www.uniprot.org/uniprotkb/W9X9G6/entry) |
| OG0008966 | 0 *E. dermatitidis* strains | 1 | None detected | [Copper transfer protein (A0A072P398_9EURO)](https://www.uniprot.org/uniprotkb/A0A072P398/entry) |
|  |  |  |  |  |
| OG0008977 | 1 DCF04 *E. dermatitidis* | 0 | [IPR001138](https://www.ebi.ac.uk/interpro/entry/InterPro/IPR001138/), [PF00172](https://www.ebi.ac.uk/interpro/entry/pfam/PF00172/), [IPR036864](https://www.ebi.ac.uk/interpro/entry/InterPro/IPR036864/) | [Zn(2)-C6 fungal-type domain-containing protein (H6BKX6_EXODN)](https://www.uniprot.org/uniprotkb/H6BKX6/entry) |
| OG0008978 | 1 DCF04 *E. dermatitidis* | 0 | [PF13095](https://www.ebi.ac.uk/interpro/set/pfam/cl0016/), [IPR025213](https://www.uniprot.org/uniprotkb/Q9US26/entry) | [uncharacterized protein (H6BPV3_EXODN)](https://www.uniprot.org/uniprotkb/H6BPV3/entry) |
| OG0009073 | 1 DCF04 *E. dermatitidis* | 0 | None detected | [uncharacterized protein (H6BPV3_EXODN)](https://www.uniprot.org/uniprotkb/H6BPV3/entry) |
| OG0009116 | 1 DCF04 *E. dermatitidis* | 0 | None detected | [uncharacterized protein (H6BWH9_EXODN)](https://www.uniprot.org/uniprotkb/H6BWH9/entry) |
| OG0009184 | 1 DCF04 *E. dermatitidis* | 0 | None detected | [uncharacterized protein (H6C0U0_EXODN)](https://www.uniprot.org/uniprotkb/H6C0U0/entry) |
| OG0009321 | 1 DCF04 *E. dermatitidis* | 0 | None detected | [uncharacterized protein (H6C7K6_EXODN)](https://www.uniprot.org/uniprotkb/H6C7K6/entry) |
| OG0009322 | 1 DCF04 *E. dermatitidis* | 0 | None detected | [uncharacterized protein (H6C7K7_EXODN)](https://www.uniprot.org/uniprotkb/H6C7K7/entry) |
