# Supplementary material for: In host evolution of Exophiala dermatitidis in cystic fibrosis lung micro-environment
Source: G3 (Bethesda). 2023 Jun 9;13(8):jkad126. doi: 10.1093/g3journal/jkad126 (PMC10484061; doi:10.1093/g3journal/jkad126)
Supplement: jkad126_Supplementary_Data [file jkad126_supplementary_data.zip › Supplemental_Table_7_G3-2023-404223.docx]

**Supplemental Table 7. All Functional SNP and INDEL results for Early and Late Pairs.**

What is the reference.

| *Pairwise Difference (Early vs Late)* | *Function https://fungidb.org/gene/HMPREF1120* |  |
| --- | --- | --- |
| *Ex1 & Ex12*  *15* | *hypothetical protein* [*HMPREF1120_00373*](https://fungidb.org/fungidb/app/record/gene/HMPREF1120_00373) *[Exophiala dermatitidis NIH/UT8656]*  *hypothetical protein* [*HMPREF1120_01565*](https://fungidb.org/fungidb/app/record/gene/HMPREF1120_01565) *[Exophiala dermatitidis NIH/UT8656]*  *MADS-box transcription factor* [*HMPREF1120_04857*](https://fungidb.org/fungidb/app/record/gene/HMPREF1120_04857) *[Exophiala dermatitidis NIH/UT8656]*  *G4 quadruplex nucleic acid binding protein [Exophiala dermatitidis NIH/UT8656]*  *hypothetical protein* [*HMPREF1120_02735*](https://fungidb.org/fungidb/app/record/gene/HMPREF1120_02735) *[Exophiala dermatitidis NIH/UT8656]*  *mitogen-activated protein kinase kinase kinase* [*HMPREF1120_04310*](https://fungidb.org/fungidb/app/record/gene/HMPREF1120_04310) *[Exophiala dermatitidis NIH/UT8656]*  *hypothetical protein* [*HMPREF1120_04204*](https://fungidb.org/fungidb/app/record/gene/HMPREF1120_04204) *[Exophiala dermatitidis NIH/UT8656]*  *hypothetical protein* [*HMPREF1120_04822*](https://fungidb.org/fungidb/app/record/gene/HMPREF1120_04822) *[Exophiala dermatitidis NIH/UT8656]*  *hypothetical protein* [*HMPREF1120_05199*](https://fungidb.org/fungidb/app/record/gene/HMPREF1120_05199) *[Exophiala dermatitidis NIH/UT8656]*  *hypothetical protein* [*HMPREF1120_05928*](https://fungidb.org/fungidb/app/record/gene/HMPREF1120_05928) *[Exophiala dermatitidis NIH/UT8656]*  *hypothetical protein* [*HMPREF1120_06924*](https://fungidb.org/fungidb/app/record/gene/HMPREF1120_06924) *[Exophiala dermatitidis NIH/UT8656]*  *AFG2 - ATPase of the CDC48/PAS1/SEC18 (AAA) family [Exophiala dermatitidis NIH/UT8656]*  *ALG2_2 - Alpha-1,3/1,6-mannosyltransferase [Exophiala dermatitidis NIH/UT8656]*  *DNA polymerase alpha subunit A* [*HMPREF1120_07994*](https://fungidb.org/fungidb/app/record/gene/HMPREF1120_07994) *[Exophiala dermatitidis NIH/UT8656]*  *GYP1 - Cis-golgi GTPase-activating protein [Exophiala dermatitidis NIH/UT8656]* |  |
| *Ex1 & Ex14*  *14* | *MADS-box transcription factor* [*HMPREF1120_04857*](https://fungidb.org/fungidb/app/record/gene/HMPREF1120_04857) *[Exophiala dermatitidis NIH/UT8656]*  *G4 quadruplex nucleic acid binding protein [Exophiala dermatitidis NIH/UT8656]*  *hypothetical protein* [*HMPREF1120_02735*](https://fungidb.org/fungidb/app/record/gene/HMPREF1120_02735) *[Exophiala dermatitidis NIH/UT8656]*  *hypothetical protein* [*HMPREF1120_04822*](https://fungidb.org/fungidb/app/record/gene/HMPREF1120_04822) *[Exophiala dermatitidis NIH/UT8656]*  *hypothetical protein* [*HMPREF1120_05199*](https://fungidb.org/fungidb/app/record/gene/HMPREF1120_05199) *[Exophiala dermatitidis NIH/UT8656]*  *monoamine oxidase* [*HMPREF1120_05597*](https://fungidb.org/fungidb/app/record/gene/HMPREF1120_05597) *[Exophiala dermatitidis NIH/UT8656]*  *biphenyl-2,3-diol 1,2-dioxygenase, variant* [*HMPREF1120_05880*](https://fungidb.org/fungidb/app/record/gene/HMPREF1120_05880) *[Exophiala dermatitidis NIH/UT8656]*  *hypothetical protein* [*HMPREF1120_05928*](https://fungidb.org/fungidb/app/record/gene/HMPREF1120_05928) *[Exophiala dermatitidis NIH/UT8656]*  *hypothetical protein* [*HMPREF1120_06924*](https://fungidb.org/fungidb/app/record/gene/HMPREF1120_06924) *[Exophiala dermatitidis NIH/UT8656]*  *AFG2 - ATPase of the CDC48/PAS1/SEC18 (AAA) family [Exophiala dermatitidis NIH/UT8656]*  *ALG2_2 - Alpha-1,3/1,6-mannosyltransferase [Exophiala dermatitidis NIH/UT8656]*  *DNA polymerase alpha subunit A* [*HMPREF1120_07994*](https://fungidb.org/fungidb/app/record/gene/HMPREF1120_07994) *[Exophiala dermatitidis NIH/UT8656]*  *hypothetical protein* [*HMPREF1120_08201*](https://fungidb.org/fungidb/app/record/gene/HMPREF1120_08201) *[Exophiala dermatitidis NIH/UT8656]*  *GYP1 - Cis-golgi GTPase-activating protein [Exophiala dermatitidis NIH/UT8656]* |  |
| *Ex2 & Ex12*  *12* | *hypothetical protein* [*HMPREF1120_00373*](https://fungidb.org/fungidb/app/record/gene/HMPREF1120_00373) *[Exophiala dermatitidis NIH/UT8656]*  *hypothetical protein* [*HMPREF1120_01565*](https://fungidb.org/fungidb/app/record/gene/HMPREF1120_01565) *[Exophiala dermatitidis NIH/UT8656]*  *MADS-box transcription factor* [*HMPREF1120_04857*](https://fungidb.org/fungidb/app/record/gene/HMPREF1120_04857) *[Exophiala dermatitidis NIH/UT8656]*  *G4 quadruplex nucleic acid binding protein [Exophiala dermatitidis NIH/UT8656]*  *mitogen-activated protein kinase kinase kinase* [*HMPREF1120_04310*](https://fungidb.org/fungidb/app/record/gene/HMPREF1120_04310) *[Exophiala dermatitidis NIH/UT8656]*  *MFS transporter, SP family, sugar:H+ symporter* [*HMPREF1120_06771*](https://fungidb.org/fungidb/app/record/gene/HMPREF1120_06771) *[Exophiala dermatitidis NIH/UT8656]*  *hypothetical protein* [*HMPREF1120_04204*](https://fungidb.org/fungidb/app/record/gene/HMPREF1120_04204) *[Exophiala dermatitidis NIH/UT8656]*  *hypothetical protein* [*HMPREF1120_04822*](https://fungidb.org/fungidb/app/record/gene/HMPREF1120_04822) *[Exophiala dermatitidis NIH/UT8656]*  *hypothetical protein* [*HMPREF1120_05199*](https://fungidb.org/fungidb/app/record/gene/HMPREF1120_05199) *[Exophiala dermatitidis NIH/UT8656]*  *BUD4 - Anillin-like protein involved in bud-site selection [Exophiala dermatitidis NIH/UT8656]*  *hypothetical protein* [*HMPREF1120_06469*](https://fungidb.org/fungidb/app/record/gene/HMPREF1120_06469) *[Exophiala dermatitidis NIH/UT8656]*  *hypothetical protein* [*HMPREF1120_08370*](https://fungidb.org/fungidb/app/record/gene/HMPREF1120_08370) *[Exophiala dermatitidis NIH/UT8656]* |  |
| *Ex2 & Ex14*  *9* | *MADS-box transcription factor* [*HMPREF1120_04857*](https://fungidb.org/fungidb/app/record/gene/HMPREF1120_04857) *[Exophiala dermatitidis NIH/UT8656]*  *G4 quadruplex nucleic acid binding protein[Exophiala dermatitidis NIH/UT8656]*  *MFS transporter, SP family, sugar:H+ symporter* [*HMPREF1120_06771*](https://fungidb.org/fungidb/app/record/gene/HMPREF1120_06771) *[Exophiala dermatitidis NIH/UT8656]*  *hypothetical protein* [*HMPREF1120_04822*](https://fungidb.org/fungidb/app/record/gene/HMPREF1120_04822) *[Exophiala dermatitidis NIH/UT8656]*  *monoamine oxidase [*[*HMPREF1120_05597*](https://fungidb.org/fungidb/app/record/gene/HMPREF1120_05597) *[Exophiala dermatitidis NIH/UT8656]*  *biphenyl-2,3-diol 1,2-dioxygenase, variant* [*HMPREF1120_05880*](https://fungidb.org/fungidb/app/record/gene/HMPREF1120_05880) *[Exophiala dermatitidis NIH/UT8656]*  *BUD4 - Anillin-like protein involved in bud-site selection [Exophiala dermatitidis NIH/UT8656]*  *hypothetical protein* [*HMPREF1120_08201*](https://fungidb.org/fungidb/app/record/gene/HMPREF1120_08201) *[Exophiala dermatitidis NIH/UT8656]*  *hypothetical protein* [*HMPREF1120_08370*](https://fungidb.org/fungidb/app/record/gene/HMPREF1120_08370) *[Exophiala dermatitidis NIH/UT8656]* |  |
| *Ex7 & Ex16*  *14* | *hypothetical protein* [*HMPREF1120_00271*](https://fungidb.org/fungidb/app/record/gene/HMPREF1120_00271) *[Exophiala dermatitidis NIH/UT8656]*  *hypothetical protein* [*HMPREF1120_00303*](https://fungidb.org/fungidb/app/record/gene/HMPREF1120_00303) *[Exophiala dermatitidis NIH/UT8656]*  *hypothetical protein* [*HMPREF1120_01139*](https://fungidb.org/fungidb/app/record/gene/HMPREF1120_01139) *[Exophiala dermatitidis NIH/UT8656]*  *hypothetical protein* [*HMPREF1120_02190*](https://fungidb.org/fungidb/app/record/gene/HMPREF1120_02190) *[Exophiala dermatitidis NIH/UT8656]*  *ADA HAT complex component 1* [*HMPREF1120_03635*](https://fungidb.org/fungidb/app/record/gene/HMPREF1120_03635) *[Exophiala dermatitidis NIH/UT8656]*  *MFS transporter, SP family, sugar:H+ symporter* [*HMPREF1120_06771*](https://fungidb.org/fungidb/app/record/gene/HMPREF1120_06771)  *[Exophiala dermatitidis NIH/UT8656]*  *NAD-dependent histone deacetylase SIR2* [*HMPREF1120_07852*](https://fungidb.org/fungidb/app/record/gene/HMPREF1120_07852)*[Exophiala dermatitidis NIH/UT8656]*  *cytochrome P450, family 7, subfamily B (oxysterol 7-alpha-hydroxylase)* [*HMPREF1120_04581*](https://fungidb.org/fungidb/app/record/gene/HMPREF1120_04581) *[Exophiala dermatitidis NIH/UT8656]*  *hypothetical protein* [*HMPREF1120_04596*](https://fungidb.org/fungidb/app/record/gene/HMPREF1120_04596) *[Exophiala dermatitidis NIH/UT8656]*  *PAN3 - PABP1-Dependent Poly A-Specific Ribonuclease Subunit* [*HMPREF1120_05279*](https://fungidb.org/fungidb/app/record/gene/HMPREF1120_05279) *[Exophiala dermatitidis NIH/UT8656]*  *hypothetical protein* [*HMPREF1120_07418*](https://fungidb.org/fungidb/app/record/gene/HMPREF1120_07418) *[Exophiala dermatitidis NIH/UT8656]*  *queuine tRNA-ribosyltransferase* [*HMPREF1120_07977*](https://fungidb.org/fungidb/app/record/gene/HMPREF1120_07977)*[Exophiala dermatitidis NIH/UT8656]*  *hypothetical protein* [*HMPREF1120_08115*](https://fungidb.org/fungidb/app/record/gene/HMPREF1120_08115) *[Exophiala dermatitidis NIH/UT8656]*  *HemK protein* [*HMPREF1120_08430*](https://fungidb.org/fungidb/app/record/gene/HMPREF1120_08430)*[Exophiala dermatitidis NIH/UT8656]* |  |
| *Ex7 & Ex17*  *7* | *hypothetical protein* [*HMPREF1120_00271*](https://fungidb.org/fungidb/app/record/gene/HMPREF1120_00271) *[Exophiala dermatitidis NIH/UT8656]*  *hypothetical protein* [*HMPREF1120_01760*](https://fungidb.org/fungidb/app/record/gene/HMPREF1120_01760) *[Exophiala dermatitidis NIH/UT8656]*  *Strongly-conserved Zn-finger binding protein (TFIIIA) [Exophiala dermatitidis NIH/UT8656]*  *hypothetical protein* [*HMPREF1120_02190*](https://fungidb.org/fungidb/app/record/gene/HMPREF1120_02190) *[Exophiala dermatitidis NIH/UT8656]*  *ADA HAT complex component 1* [*HMPREF1120_03635*](https://fungidb.org/fungidb/app/record/gene/HMPREF1120_03635) *[Exophiala dermatitidis NIH/UT8656]*  *PAN3 - PABP1-Dependent Poly A-Specific Ribonuclease Subunit* [*HMPREF1120_05279*](https://fungidb.org/fungidb/app/record/gene/HMPREF1120_05279) *[Exophiala dermatitidis NIH/UT8656]*  *hypothetical protein* [*HMPREF1120_06447*](https://fungidb.org/fungidb/app/record/gene/HMPREF1120_06447) *[Exophiala dermatitidis NIH/UT8656]* |  |
| *Ex7 & Ex19*  *14* | *hypothetical protein* [*HMPREF1120_00271*](https://fungidb.org/fungidb/app/record/gene/HMPREF1120_00271) *[Exophiala dermatitidis NIH/UT8656]*  *hypothetical protein* [*HMPREF1120_01139*](https://fungidb.org/fungidb/app/record/gene/HMPREF1120_01139) *[Exophiala dermatitidis NIH/UT8656]*  *alpha-1,2-mannosyltransferase* [*HMPREF1120_07833*](https://fungidb.org/fungidb/app/record/gene/HMPREF1120_07833)*[Exophiala dermatitidis NIH/UT8656]*  *ribosome biosynthesis protein rrb1[Exophiala dermatitidis NIH/UT8656]*  *hypothetical protein* [*HMPREF1120_05142*](https://fungidb.org/fungidb/app/record/gene/HMPREF1120_05142) *[Exophiala dermatitidis NIH/UT8656]*  *ADA HAT complex component 1* [*HMPREF1120_03635*](https://fungidb.org/fungidb/app/record/gene/HMPREF1120_03635) *[Exophiala dermatitidis NIH/UT8656]*  *MFS transporter, SP family, sugar:H+ symporter* [*HMPREF1120_06771*](https://fungidb.org/fungidb/app/record/gene/HMPREF1120_06771)  *[Exophiala dermatitidis NIH/UT8656]*  *hypothetical protein* [*HMPREF1120_04204*](https://fungidb.org/fungidb/app/record/gene/HMPREF1120_04204) *[Exophiala dermatitidis NIH/UT8656]*  *DUS3 - Dihydrouridine synthase* [*HMPREF1120_04489*](https://fungidb.org/fungidb/app/record/gene/HMPREF1120_04489) *[Exophiala dermatitidis NIH/UT8656]*  *hypothetical protein* [*HMPREF1120_04676*](https://fungidb.org/fungidb/app/record/gene/HMPREF1120_04676) *[Exophiala dermatitidis NIH/UT8656]*  *PAN3 - PABP1-Dependent Poly A-Specific Ribonuclease Subunit* [*HMPREF1120_05279*](https://fungidb.org/fungidb/app/record/gene/HMPREF1120_05279) *[Exophiala dermatitidis NIH/UT8656]*  *monoamine oxidase* [*HMPREF1120_05597*](https://fungidb.org/fungidb/app/record/gene/HMPREF1120_05597) *[Exophiala dermatitidis NIH/UT8656]*  *hypothetical protein* [*HMPREF1120_06612*](https://fungidb.org/fungidb/app/record/gene/HMPREF1120_06612) *[Exophiala dermatitidis NIH/UT8656]*  *hypothetical protein* [*HMPREF1120_08252*](https://fungidb.org/fungidb/app/record/gene/HMPREF1120_08252) *[Exophiala dermatitidis NIH/UT8656]* |  |
| *Ex7 & Ex22*  *6* | *hypothetical protein* [*HMPREF1120_00271*](https://fungidb.org/fungidb/app/record/gene/HMPREF1120_00271) *[Exophiala dermatitidis NIH/UT8656]*  *hypothetical protein* [*HMPREF1120_01139*](https://fungidb.org/fungidb/app/record/gene/HMPREF1120_01139) *[Exophiala dermatitidis NIH/UT8656]*  *hypothetical protein* [*HMPREF1120_02190*](https://fungidb.org/fungidb/app/record/gene/HMPREF1120_02190) *[Exophiala dermatitidis NIH/UT8656]*  *ADA HAT complex component 1* [*HMPREF1120_03635*](https://fungidb.org/fungidb/app/record/gene/HMPREF1120_03635) *[Exophiala dermatitidis NIH/UT8656]*  *Ring finger and CHY zinc finger domain-containing protein 1* [*HMPREF1120_04104*](https://fungidb.org/fungidb/app/record/gene/HMPREF1120_04104) *[Exophiala dermatitidis NIH/UT8656]*  *PAN3 - PABP1-Dependent Poly A-Specific Ribonuclease Subunit* [*HMPREF1120_05279*](https://fungidb.org/fungidb/app/record/gene/HMPREF1120_05279) *[Exophiala dermatitidis NIH/UT8656]* |  |
| *Ex7 & Ex23*  *22* | *hypothetical protein* [*HMPREF1120_00215*](https://fungidb.org/fungidb/app/record/gene/HMPREF1120_00215) *[Exophiala dermatitidis NIH/UT8656]*  *hypothetical protein* [*HMPREF1120_00271*](https://fungidb.org/fungidb/app/record/gene/HMPREF1120_00271) *[Exophiala dermatitidis NIH/UT8656]*  *hypothetical protein* [*HMPREF1120_01244*](https://fungidb.org/fungidb/app/record/gene/HMPREF1120_01244) *[Exophiala dermatitidis NIH/UT8656]*  *hypothetical protein* [*HMPREF1120_01346*](https://fungidb.org/fungidb/app/record/gene/HMPREF1120_01346) *[Exophiala dermatitidis NIH/UT8656]*  *MFS transporter, DHA1 family, multidrug resistance protein* [*HMPREF1120_09017*](https://fungidb.org/fungidb/app/record/gene/HMPREF1120_09017) *[Exophiala dermatitidis NIH/UT8656]*  *transcriptional regulatory protein GAL4* [*HMPREF1120_08700*](https://fungidb.org/fungidb/app/record/gene/HMPREF1120_08700) *[Exophiala dermatitidis NIH/UT8656]*  *G4 quadruplex nucleic acid binding protein [Exophiala dermatitidis NIH/UT8656]*  *ribosome biosynthesis protein rrb1[Exophiala dermatitidis NIH/UT8656]*  *hypothetical protein* [*HMPREF1120_02823*](https://fungidb.org/fungidb/app/record/gene/HMPREF1120_02823) *[Exophiala dermatitidis NIH/UT8656]*  *hypothetical protein* [*HMPREF1120_05142*](https://fungidb.org/fungidb/app/record/gene/HMPREF1120_05142) *[Exophiala dermatitidis NIH/UT8656]*  *2,3-bisphosphoglycerate-dependent phosphoglycerate mutase* [*HMPREF1120_02960*](https://fungidb.org/fungidb/app/record/gene/HMPREF1120_02960) *[Exophiala dermatitidis NIH/UT8656]*  *phosphatidylinositol-bisphosphatase* [*HMPREF1120_04965*](https://fungidb.org/fungidb/app/record/gene/HMPREF1120_04965) *[Exophiala dermatitidis NIH/UT8656]*  *ADA HAT complex component 1* [*HMPREF1120_03635*](https://fungidb.org/fungidb/app/record/gene/HMPREF1120_03635) *[Exophiala dermatitidis NIH/UT8656]*  *hypothetical protein* [*HMPREF1120_04204*](https://fungidb.org/fungidb/app/record/gene/HMPREF1120_04204) *[Exophiala dermatitidis NIH/UT8656]*  *hypothetical protein* [*HMPREF1120_04403*](https://fungidb.org/fungidb/app/record/gene/HMPREF1120_04403) *[Exophiala dermatitidis NIH/UT8656]*  *PAN3 - PABP1-Dependent Poly A-Specific Ribonuclease Subunit* [*HMPREF1120_05279*](https://fungidb.org/fungidb/app/record/gene/HMPREF1120_05279) *[Exophiala dermatitidis NIH/UT8656]*  *nucleolin* [*HMPREF1120_07826*](https://fungidb.org/fungidb/app/record/gene/HMPREF1120_07826) *[Exophiala dermatitidis NIH/UT8656]*  *hypothetical protein* [*HMPREF1120_05877*](https://fungidb.org/fungidb/app/record/gene/HMPREF1120_05877) *[Exophiala dermatitidis NIH/UT8656]*  *hypothetical protein* [*HMPREF1120_05976*](https://fungidb.org/fungidb/app/record/gene/HMPREF1120_05976) *[Exophiala dermatitidis NIH/UT8656]*  *inositol oxygenase* [*HMPREF1120_07129*](https://fungidb.org/fungidb/app/record/gene/HMPREF1120_07129)*[Exophiala dermatitidis NIH/UT8656]*  *hypothetical protein* [*HMPREF1120_09068*](https://fungidb.org/fungidb/app/record/gene/HMPREF1120_09068) *[Exophiala dermatitidis NIH/UT8656]*  *hypothetical protein* [*HMPREF1120_09075*](https://fungidb.org/fungidb/app/record/gene/HMPREF1120_09075) *[Exophiala dermatitidis NIH/UT8656]* |  |
| *Ex5 & Ex15*  *120* | *DNA repair protein RAD50* [*HMPREF1120_04505*](https://fungidb.org/fungidb/app/record/gene/HMPREF1120_04505)*[Exophiala dermatitidis NIH/UT8656]*  *hypothetical protein* [*HMPREF1120_00457*](https://fungidb.org/fungidb/app/record/gene/HMPREF1120_00457) *[Exophiala dermatitidis NIH/UT8656]*  *hypothetical protein* [*HMPREF1120_00646*](https://fungidb.org/fungidb/app/record/gene/HMPREF1120_00646) *[Exophiala dermatitidis NIH/UT8656]*  *eukaryotic translation initiation factor 2 subunit gamma* [*HMPREF1120_00677*](https://fungidb.org/fungidb/app/record/gene/HMPREF1120_00677)  *[Exophiala dermatitidis NIH/UT8656]*  *cytochrome P450 oxidoreductase* [*HMPREF1120_01361*](https://fungidb.org/fungidb/app/record/gene/HMPREF1120_01361) *[Exophiala dermatitidis NIH/UT8656]*  *hypothetical protein* [*HMPREF1120_00830*](https://fungidb.org/fungidb/app/record/gene/HMPREF1120_00830) *[Exophiala dermatitidis NIH/UT8656]*  *hypothetical protein* [*HMPREF1120_01089*](https://fungidb.org/fungidb/app/record/gene/HMPREF1120_01089) *[Exophiala dermatitidis NIH/UT8656]*  *hypothetical protein* [*HMPREF1120_01151*](https://fungidb.org/fungidb/app/record/gene/HMPREF1120_01151) *[Exophiala dermatitidis NIH/UT8656]*  *sulfite oxidase* [*HMPREF1120_05227*](https://fungidb.org/fungidb/app/record/gene/HMPREF1120_05227) *[Exophiala dermatitidis NIH/UT8656]*  *prolyl-tRNA synthetase* [*HMPREF1120_01354*](https://fungidb.org/fungidb/app/record/gene/HMPREF1120_01354) *[Exophiala dermatitidis NIH/UT8656]*  *hypothetical protein* [*HMPREF1120_01471*](https://fungidb.org/fungidb/app/record/gene/HMPREF1120_01471) *[Exophiala dermatitidis NIH/UT8656]*  *serine/threonine kinase 16* [*HMPREF1120_06920*](https://fungidb.org/fungidb/app/record/gene/HMPREF1120_06920) *[Exophiala dermatitidis NIH/UT8656]*  *hypothetical protein* [*HMPREF1120_02055*](https://fungidb.org/fungidb/app/record/gene/HMPREF1120_02055) *[Exophiala dermatitidis NIH/UT8656]*  *hypothetical protein* [*HMPREF1120_02083*](https://fungidb.org/fungidb/app/record/gene/HMPREF1120_02083) *[Exophiala dermatitidis NIH/UT8656]*  *G4 quadruplex nucleic acid binding protein [Exophiala dermatitidis NIH/UT8656]*  *hypothetical protein* [*HMPREF1120_02190*](https://fungidb.org/fungidb/app/record/gene/HMPREF1120_02190) *[Exophiala dermatitidis NIH/UT8656]*  *glutathione S-transferase* [*HMPREF1120_08143*](https://fungidb.org/fungidb/app/record/gene/HMPREF1120_08143) *[Exophiala dermatitidis NIH/UT8656]*  *nicotinate-nucleotide diphosphorylase (carboxylating)* [*HMPREF1120_02317*](https://fungidb.org/fungidb/app/record/gene/HMPREF1120_02317)  *[Exophiala dermatitidis NIH/UT8656]*  *phosphodiesterase/alkaline phosphatase D* [*HMPREF1120_02364*](https://fungidb.org/fungidb/app/record/gene/HMPREF1120_02364) *[Exophiala dermatitidis NIH/UT8656]*  *Xanthine phosphoribosyltransferase 1* [*HMPREF1120_06110*](https://fungidb.org/fungidb/app/record/gene/HMPREF1120_06110) *[Exophiala dermatitidis NIH/UT8656]*  *rRNA (cytosine-C5-)-methyltransferase nop2* [*HMPREF1120_02613*](https://fungidb.org/fungidb/app/record/gene/HMPREF1120_02613) *[Exophiala dermatitidis NIH/UT8656]*  *transformation/transcription domain-associated protein* [*HMPREF1120_02639*](https://fungidb.org/fungidb/app/record/gene/HMPREF1120_02639)  *[Exophiala dermatitidis NIH/UT8656]*  *Serine/threonine-protein phosphatase 2A 56 kDa regulatory subunit delta isoform* [*HMPREF1120_01344*](https://fungidb.org/fungidb/app/record/gene/HMPREF1120_01344) *[Exophiala dermatitidis NIH/UT8656]*  *hypothetical protein* [*HMPREF1120_02700*](https://fungidb.org/fungidb/app/record/gene/HMPREF1120_02700) *[Exophiala dermatitidis NIH/UT8656]*  *hypothetical protein* [*HMPREF1120_02708*](https://fungidb.org/fungidb/app/record/gene/HMPREF1120_02708) *[Exophiala dermatitidis NIH/UT8656]*  *hypothetical protein* [*HMPREF1120_02758*](https://fungidb.org/fungidb/app/record/gene/HMPREF1120_02758) *[Exophiala dermatitidis NIH/UT8656]*  *hypothetical protein* [*HMPREF1120_02784*](https://fungidb.org/fungidb/app/record/gene/HMPREF1120_02784) *[Exophiala dermatitidis NIH/UT8656]*  *MOT1 - TATA-binding protein-associated factor* [*HMPREF1120_06808*](https://fungidb.org/fungidb/app/record/gene/HMPREF1120_06808)  *[Exophiala dermatitidis NIH/UT8656]*  *ING3 - Inhibitor of growth protein 3* [*HMPREF1120_05286*](https://fungidb.org/fungidb/app/record/gene/HMPREF1120_05286) *[Exophiala dermatitidis NIH/UT8656]*  *hypothetical protein* [*HMPREF1120_02908*](https://fungidb.org/fungidb/app/record/gene/HMPREF1120_02908) *[Exophiala dermatitidis NIH/UT8656]*  *PDE2 - High-affinity cyclic AMP phosphodiesterase [Exophiala dermatitidis NIH/UT8656]*  *hypothetical protein* [*HMPREF1120_03003*](https://fungidb.org/fungidb/app/record/gene/HMPREF1120_03003) *[Exophiala dermatitidis NIH/UT8656]*  *hypothetical protein* [*HMPREF1120_03007*](https://fungidb.org/fungidb/app/record/gene/HMPREF1120_03007) *[Exophiala dermatitidis NIH/UT8656]*  *VE1 - Verticillium wilt disease resistance protein [Exophiala dermatitidis NIH/UT8656]*  *hypothetical protein* [*HMPREF1120_03359*](https://fungidb.org/fungidb/app/record/gene/HMPREF1120_03359) *[Exophiala dermatitidis NIH/UT8656]*  *hypothetical protein* [*HMPREF1120_03367*](https://fungidb.org/fungidb/app/record/gene/HMPREF1120_03367) *[Exophiala dermatitidis NIH/UT8656]*  *hypothetical protein* [*HMPREF1120_03513*](https://fungidb.org/fungidb/app/record/gene/HMPREF1120_03513) *[Exophiala dermatitidis NIH/UT8656]*  *gibberellin 2-oxidase* [*HMPREF1120_09208*](https://fungidb.org/fungidb/app/record/gene/HMPREF1120_09208)*[Exophiala dermatitidis NIH/UT8656]*  *hypothetical protein* [*HMPREF1120_03910*](https://fungidb.org/fungidb/app/record/gene/HMPREF1120_03910) *[Exophiala dermatitidis NIH/UT8656]*  *hypothetical protein* [*HMPREF1120_03911*](https://fungidb.org/fungidb/app/record/gene/HMPREF1120_03911) *[Exophiala dermatitidis NIH/UT8656]*  *hypothetical protein* [*HMPREF1120_03928*](https://fungidb.org/fungidb/app/record/gene/HMPREF1120_03928) *[Exophiala dermatitidis NIH/UT8656]*  *amidohydrolase* [*HMPREF1120_03964*](https://fungidb.org/fungidb/app/record/gene/HMPREF1120_03964)*[Exophiala dermatitidis NIH/UT8656]*  *hypothetical protein* [*HMPREF1120_04101*](https://fungidb.org/fungidb/app/record/gene/HMPREF1120_04101) *[Exophiala dermatitidis NIH/UT8656]*  *MFS transporter, SP family, sugar:H+ symporter* [*HMPREF1120_06771*](https://fungidb.org/fungidb/app/record/gene/HMPREF1120_06771)  *[Exophiala dermatitidis NIH/UT8656]*  *COP9 signalosome complex subunit 2* [*HMPREF1120_04182*](https://fungidb.org/fungidb/app/record/gene/HMPREF1120_04182) *[Exophiala dermatitidis NIH/UT8656]*  *hypothetical protein* [*HMPREF1120_04204*](https://fungidb.org/fungidb/app/record/gene/HMPREF1120_04204) *[Exophiala dermatitidis NIH/UT8656]*  *SIP3 - Putative sterol transfer protein* [*HMPREF1120_04207*](https://fungidb.org/fungidb/app/record/gene/HMPREF1120_04207) *[Exophiala dermatitidis NIH/UT8656]*  *hypothetical protein* [*HMPREF1120_04314*](https://fungidb.org/fungidb/app/record/gene/HMPREF1120_04314) *[Exophiala dermatitidis NIH/UT8656]*  *hypothetical protein* [*HMPREF1120_04349*](https://fungidb.org/fungidb/app/record/gene/HMPREF1120_04349) *[Exophiala dermatitidis NIH/UT8656]*  *hypothetical protein* [*HMPREF1120_04459*](https://fungidb.org/fungidb/app/record/gene/HMPREF1120_04459) *[Exophiala dermatitidis NIH/UT8656]*  *tyrosinase* [*HMPREF1120_04514*](https://fungidb.org/fungidb/app/record/gene/HMPREF1120_04514) *[Exophiala dermatitidis NIH/UT8656]*  *hypothetical protein* [*HMPREF1120_04584*](https://fungidb.org/fungidb/app/record/gene/HMPREF1120_04584) *[Exophiala dermatitidis NIH/UT8656]*  *ankyrin* [*HMPREF1120_08463*](https://fungidb.org/fungidb/app/record/gene/HMPREF1120_08463)*[Exophiala dermatitidis NIH/UT8656]*  *hypothetical protein* [*HMPREF1120_04645*](https://fungidb.org/fungidb/app/record/gene/HMPREF1120_04645) *[Exophiala dermatitidis NIH/UT8656]*  *adenosinetriphosphatase* [*HMPREF1120_09246*](https://fungidb.org/fungidb/app/record/gene/HMPREF1120_09246) *[Exophiala dermatitidis NIH/UT8656]*  *hypothetical protein* [*HMPREF1120_04673*](https://fungidb.org/fungidb/app/record/gene/HMPREF1120_04673) *[Exophiala dermatitidis NIH/UT8656]*  *alkanesulfonate monooxygenase* [*HMPREF1120_08264*](https://fungidb.org/fungidb/app/record/gene/HMPREF1120_08264)*[Exophiala dermatitidis NIH/UT8656]*  *hypothetical protein* [*HMPREF1120_04695*](https://fungidb.org/fungidb/app/record/gene/HMPREF1120_04695) *[Exophiala dermatitidis NIH/UT8656]*  *hypothetical protein* [*HMPREF1120_04835*](https://fungidb.org/fungidb/app/record/gene/HMPREF1120_04835) *[Exophiala dermatitidis NIH/UT8656]*  *hypothetical protein* [*HMPREF1120_04991*](https://fungidb.org/fungidb/app/record/gene/HMPREF1120_04991) *[Exophiala dermatitidis NIH/UT8656]*  *4-coumarate-CoA ligase* [*HMPREF1120_09210*](https://fungidb.org/fungidb/app/record/gene/HMPREF1120_09210)*[Exophiala dermatitidis NIH/UT8656]*  *hypothetical protein* [*HMPREF1120_05151*](https://fungidb.org/fungidb/app/record/gene/HMPREF1120_05151) *[Exophiala dermatitidis NIH/UT8656]*  *hypothetical protein* [*HMPREF1120_05400*](https://fungidb.org/fungidb/app/record/gene/HMPREF1120_05400) *[Exophiala dermatitidis NIH/UT8656]*  *dihydrodipicolinate synthetase* [*HMPREF1120_09161*](https://fungidb.org/fungidb/app/record/gene/HMPREF1120_09161)*[Exophiala dermatitidis NIH/UT8656]*  *hypothetical protein* [*HMPREF1120_05622*](https://fungidb.org/fungidb/app/record/gene/HMPREF1120_05622) *[Exophiala dermatitidis NIH/UT8656]*  *hypothetical protein* [*HMPREF1120_05658*](https://fungidb.org/fungidb/app/record/gene/HMPREF1120_05658) *[Exophiala dermatitidis NIH/UT8656]*  *PSY2 - Platinum sensitivity protein [Exophiala dermatitidis NIH/UT8656]*  *hypothetical protein* [*HMPREF1120_05728*](https://fungidb.org/fungidb/app/record/gene/HMPREF1120_05728) *[Exophiala dermatitidis NIH/UT8656]*  *SPT20 - Transcription factor spt20* [*HMPREF1120_05758*](https://fungidb.org/fungidb/app/record/gene/HMPREF1120_05758)*[Exophiala dermatitidis NIH/UT8656]*  *hypothetical protein* [*HMPREF1120_05809*](https://fungidb.org/fungidb/app/record/gene/HMPREF1120_05809) *[Exophiala dermatitidis NIH/UT8656]*  *ETF1 - elongation factor 2* [*HMPREF1120_05986*](https://fungidb.org/fungidb/app/record/gene/HMPREF1120_05986)*[Exophiala dermatitidis NIH/UT8656]*  *hypothetical protein* [*HMPREF1120_06093*](https://fungidb.org/fungidb/app/record/gene/HMPREF1120_06093) *[Exophiala dermatitidis NIH/UT8656]*  *RGT1 - Glucose-responsive transcription factor 1 [Exophiala dermatitidis NIH/UT8656]*  *hypothetical protein* [*HMPREF1120_06177*](https://fungidb.org/fungidb/app/record/gene/HMPREF1120_06177) *[Exophiala dermatitidis NIH/UT8656]*  *hypothetical protein* [*HMPREF1120_06361*](https://fungidb.org/fungidb/app/record/gene/HMPREF1120_06361) *[Exophiala dermatitidis NIH/UT8656]*  *37S ribosomal protein, mitochondrial [Exophiala dermatitidis NIH/UT8656]*  *hypothetical protein* [*HMPREF1120_06500*](https://fungidb.org/fungidb/app/record/gene/HMPREF1120_06500) *[Exophiala dermatitidis NIH/UT8656]*  *hypothetical protein* [*HMPREF1120_06642*](https://fungidb.org/fungidb/app/record/gene/HMPREF1120_06642) *[Exophiala dermatitidis NIH/UT8656]*  *hypothetical protein* [*HMPREF1120_06878*](https://fungidb.org/fungidb/app/record/gene/HMPREF1120_06878) *[Exophiala dermatitidis NIH/UT8656]*  *hypothetical protein* [*HMPREF1120_06971*](https://fungidb.org/fungidb/app/record/gene/HMPREF1120_06971) *[Exophiala dermatitidis NIH/UT8656]*  *transcription initiation factor TFIID subunit D2* [*MPREF1120_06984*](https://fungidb.org/fungidb/app/record/gene/HMPREF1120_06984)*[Exophiala dermatitidis NIH/UT8656]*  *L-galactose dehydrogenase* [*HMPREF1120_07000*](https://fungidb.org/fungidb/app/record/gene/HMPREF1120_07000)*[Exophiala dermatitidis NIH/UT8656]*  *hypothetical protein* [*HMPREF1120_07085*](https://fungidb.org/fungidb/app/record/gene/HMPREF1120_07085) *[Exophiala dermatitidis NIH/UT8656]*  *3' exoribonuclease* [*HMPREF1120_08304*](https://fungidb.org/fungidb/app/record/gene/HMPREF1120_08304)*[Exophiala dermatitidis NIH/UT8656]*  *hypothetical protein* [*HMPREF1120_07255*](https://fungidb.org/fungidb/app/record/gene/HMPREF1120_07255) *[Exophiala dermatitidis NIH/UT8656]*  *hypothetical protein* [*HMPREF1120_07291*](https://fungidb.org/fungidb/app/record/gene/HMPREF1120_07291) *[Exophiala dermatitidis NIH/UT8656]*  *hypothetical protein* [*HMPREF1120_07306*](https://fungidb.org/fungidb/app/record/gene/HMPREF1120_07306) *[Exophiala dermatitidis NIH/UT8656]*  *hypothetical protein* [*HMPREF1120_07433*](https://fungidb.org/fungidb/app/record/gene/HMPREF1120_07433) *[Exophiala dermatitidis NIH/UT8656]*  *RAS2 - Ras GTPase* [*HMPREF1120_01421*](https://fungidb.org/fungidb/app/record/gene/HMPREF1120_01421)*[Exophiala dermatitidis NIH/UT8656]*  *hypothetical protein* [*HMPREF1120_07616*](https://fungidb.org/fungidb/app/record/gene/HMPREF1120_07616) *[Exophiala dermatitidis NIH/UT8656]*  *D-3-phosphoglycerate dehydrogenase* [*HMPREF1120_06805*](https://fungidb.org/fungidb/app/record/gene/HMPREF1120_06805)*[Exophiala dermatitidis NIH/UT8656]*  *ankyrin* [*HMPREF1120_08463*](https://fungidb.org/fungidb/app/record/gene/HMPREF1120_08463)*[Exophiala dermatitidis NIH/UT8656]*  *nuclear transcription factor Y, alpha* [*HMPREF1120_07714*](https://fungidb.org/fungidb/app/record/gene/HMPREF1120_07714)*[Exophiala dermatitidis NIH/UT8656]*  *CHS3 - chitin synthase class 3* [*HMPREF1120_08776*](https://fungidb.org/fungidb/app/record/gene/HMPREF1120_08776) *[Exophiala dermatitidis NIH/UT8656]*  *salicylate hydroxylase* [*HMPREF1120_03459*](https://fungidb.org/fungidb/app/record/gene/HMPREF1120_03459) *[Exophiala dermatitidis NIH/UT8656]*  *hypothetical protein* [*HMPREF1120_06589*](https://fungidb.org/fungidb/app/record/gene/HMPREF1120_06589) *[Exophiala dermatitidis NIH/UT8656]*  *hypothetical protein* [*HMPREF1120_06584*](https://fungidb.org/fungidb/app/record/gene/HMPREF1120_06584) *[Exophiala dermatitidis NIH/UT8656]*  *queuine tRNA-ribosyltransferase* [*HMPREF1120_07977*](https://fungidb.org/fungidb/app/record/gene/HMPREF1120_07977)*[Exophiala dermatitidis NIH/UT8656]*  *hypothetical protein* [*HMPREF1120_07985*](https://fungidb.org/fungidb/app/record/gene/HMPREF1120_07985) *[Exophiala dermatitidis NIH/UT8656]*  *thiamin biosynthesis protein* [*HMPREF1120_07987*](https://fungidb.org/fungidb/app/record/gene/HMPREF1120_07987)*[Exophiala dermatitidis NIH/UT8656]*  *ABD1 - mRNA cap guanine-N7 methyltransferase* [*HMPREF1120_06541*](https://fungidb.org/fungidb/app/record/gene/HMPREF1120_06541)*[Exophiala dermatitidis NIH/UT8656]*  *PAN2 - poly(A) specific ribonuclease [Exophiala dermatitidis NIH/UT8656]*  *DOA4 - ubiquitin specific protease* [*HMPREF1120_06573*](https://fungidb.org/fungidb/app/record/gene/HMPREF1120_06573)*[Exophiala dermatitidis NIH/UT8656]*  *amidase* [*HMPREF1120_09153*](https://fungidb.org/fungidb/app/record/gene/HMPREF1120_09153)*[Exophiala dermatitidis NIH/UT8656]*  *MEF2 - Ribosome-releasing factor 2, mitochondrial[Exophiala dermatitidis NIH/UT8656]*  *RGA2 - Rho-type gtpase-activating protein[Exophiala dermatitidis NIH/UT8656]*  *hypothetical protein* [*HMPREF1120_08236*](https://fungidb.org/fungidb/app/record/gene/HMPREF1120_08236) *[Exophiala dermatitidis NIH/UT8656]*  *cytochrome P450 oxidoreductase* [*HMPREF1120_01361*](https://fungidb.org/fungidb/app/record/gene/HMPREF1120_01361) *[Exophiala dermatitidis NIH/UT8656]*  *hypothetical protein* [*HMPREF1120_08425*](https://fungidb.org/fungidb/app/record/gene/HMPREF1120_08425) *[Exophiala dermatitidis NIH/UT8656]*  *hydrolase* [*HMPREF1120_08460*](https://fungidb.org/fungidb/app/record/gene/HMPREF1120_08460)*[Exophiala dermatitidis NIH/UT8656]*  *hypothetical protein* [*HMPREF1120_08629*](https://fungidb.org/fungidb/app/record/gene/HMPREF1120_08629) *[Exophiala dermatitidis NIH/UT8656]*  *pyruvate carboxylase* [*HMPREF1120_09185*](https://fungidb.org/fungidb/app/record/gene/HMPREF1120_09185) *[Exophiala dermatitidis NIH/UT8656]*  *hypothetical protein* [*HMPREF1120_08890*](https://fungidb.org/fungidb/app/record/gene/HMPREF1120_08890) *[Exophiala dermatitidis NIH/UT8656]*  *hypothetical protein* [*HMPREF1120_09031*](https://fungidb.org/fungidb/app/record/gene/HMPREF1120_09031) *[Exophiala dermatitidis NIH/UT8656]*  *hypothetical protein* [*HMPREF1120_09084*](https://fungidb.org/fungidb/app/record/gene/HMPREF1120_09084) *[Exophiala dermatitidis NIH/UT8656]*  *chitin synthase* [*HMPREF1120_08777*](https://fungidb.org/fungidb/app/record/gene/HMPREF1120_08777) *[Exophiala dermatitidis NIH/UT8656]*  *hypothetical protein* [*HMPREF1120_09220*](https://fungidb.org/fungidb/app/record/gene/HMPREF1120_09220) *[Exophiala dermatitidis NIH/UT8656]*  *ribonuclease H2 subunit A* [*HMPREF1120_09244*](https://fungidb.org/fungidb/app/record/gene/HMPREF1120_09244) *[Exophiala dermatitidis NIH/UT8656]* |  |
| *Ex5 & Ex18*  *148* | *MFS transporter, DHA2 family, methylenomycin A resistance protein* [*HMPREF1120_00012*](https://fungidb.org/fungidb/app/record/gene/HMPREF1120_00012) *[Exophiala dermatitidis NIH/UT8656]*  *DNA repair protein RAD50* [*HMPREF1120_04505*](https://fungidb.org/fungidb/app/record/gene/HMPREF1120_04505) *[Exophiala dermatitidis NIH/UT8656]*  *hypothetical protein* [*HMPREF1120_00457*](https://fungidb.org/fungidb/app/record/gene/HMPREF1120_00457) *[Exophiala dermatitidis NIH/UT8656]*  *inositol polyphosphate 5-phosphatase* [*HMPREF1120_01286*](https://fungidb.org/fungidb/app/record/gene/HMPREF1120_01286)*[Exophiala dermatitidis NIH/UT8656]*  *hypothetical protein* [*HMPREF1120_00487*](https://fungidb.org/fungidb/app/record/gene/HMPREF1120_00487) *[Exophiala dermatitidis NIH/UT8656]*  *hypothetical protein* [*HMPREF1120_00646*](https://fungidb.org/fungidb/app/record/gene/HMPREF1120_00646) *[Exophiala dermatitidis NIH/UT8656]*  *DEAD/DEAH box RNA helicase* [*HMPREF1120_00651*](https://fungidb.org/fungidb/app/record/gene/HMPREF1120_00651)*[Exophiala dermatitidis NIH/UT8656]*  *eukaryotic translation initiation factor 2 subunit gamma* [*HMPREF1120_00677*](https://fungidb.org/fungidb/app/record/gene/HMPREF1120_00677)  *[Exophiala dermatitidis NIH/UT8656]*  *cytochrome P450 oxidoreductase* [*HMPREF1120_01361*](https://fungidb.org/fungidb/app/record/gene/HMPREF1120_01361) *[Exophiala dermatitidis NIH/UT8656]*  *sulfite reductase (ferredoxin)* [*HMPREF1120_00943*](https://fungidb.org/fungidb/app/record/gene/HMPREF1120_00943)*[Exophiala dermatitidis NIH/UT8656]*  *glycerol ethanol, ferric requiring protein [Exophiala dermatitidis NIH/UT8656]*  *hypothetical protein* [*HMPREF1120_01089*](https://fungidb.org/fungidb/app/record/gene/HMPREF1120_01089) *[Exophiala dermatitidis NIH/UT8656]*  *hypothetical protein* [*HMPREF1120_01151*](https://fungidb.org/fungidb/app/record/gene/HMPREF1120_01151) *[Exophiala dermatitidis NIH/UT8656]*  *sulfite oxidase* [*HMPREF1120_05227*](https://fungidb.org/fungidb/app/record/gene/HMPREF1120_05227) *[Exophiala dermatitidis NIH/UT8656]*  *prolyl-tRNA synthetase* [*HMPREF1120_01354*](https://fungidb.org/fungidb/app/record/gene/HMPREF1120_01354) *[Exophiala dermatitidis NIH/UT8656]*  *cytochrome P450 oxidoreductase* [*HMPREF1120_01361*](https://fungidb.org/fungidb/app/record/gene/HMPREF1120_01361) *[Exophiala dermatitidis NIH/UT8656]*  *hypothetical protein* [*HMPREF1120_01471*](https://fungidb.org/fungidb/app/record/gene/HMPREF1120_01471) *[Exophiala dermatitidis NIH/UT8656]*  *serine/threonine kinase 16* [*HMPREF1120_06920*](https://fungidb.org/fungidb/app/record/gene/HMPREF1120_06920) *[Exophiala dermatitidis NIH/UT8656]*  *hypothetical protein* [*HMPREF1120_01961*](https://fungidb.org/fungidb/app/record/gene/HMPREF1120_01961) *[Exophiala dermatitidis NIH/UT8656]*  *hypothetical protein* [*HMPREF1120_02083*](https://fungidb.org/fungidb/app/record/gene/HMPREF1120_02083) *[Exophiala dermatitidis NIH/UT8656]*  *hypothetical protein* [*HMPREF1120_02190*](https://fungidb.org/fungidb/app/record/gene/HMPREF1120_02190) *[Exophiala dermatitidis NIH/UT8656]*  *MFS transporter, DHA1 family, multidrug resistance protein* [*HMPREF1120_09017*](https://fungidb.org/fungidb/app/record/gene/HMPREF1120_09017) *[Exophiala dermatitidis NIH/UT8656]*  *glutathione S-transferase* [*HMPREF1120_08143*](https://fungidb.org/fungidb/app/record/gene/HMPREF1120_08143) *[Exophiala dermatitidis NIH/UT8656]*  *nicotinate-nucleotide diphosphorylase (carboxylating)* [*HMPREF1120_02317*](https://fungidb.org/fungidb/app/record/gene/HMPREF1120_02317)  *[Exophiala dermatitidis NIH/UT8656]*  *phosphodiesterase/alkaline phosphatase D* [*HMPREF1120_02364*](https://fungidb.org/fungidb/app/record/gene/HMPREF1120_02364) *[Exophiala dermatitidis NIH/UT8656]*  *hypothetical protein* [*HMPREF1120_02386*](https://fungidb.org/fungidb/app/record/gene/HMPREF1120_02386) *[Exophiala dermatitidis NIH/UT8656]*  *Xanthine phosphoribosyltransferase 1* [*HMPREF1120_06110*](https://fungidb.org/fungidb/app/record/gene/HMPREF1120_06110) *[Exophiala dermatitidis NIH/UT8656]*  *hypothetical protein* [*HMPREF1120_02597*](https://fungidb.org/fungidb/app/record/gene/HMPREF1120_02597) *[Exophiala dermatitidis NIH/UT8656]*  *rRNA (cytosine-C5-)-methyltransferase nop2* [*HMPREF1120_02613*](https://fungidb.org/fungidb/app/record/gene/HMPREF1120_02613) *[Exophiala dermatitidis NIH/UT8656]*  *transformation/transcription domain-associated protein* [*HMPREF1120_02639*](https://fungidb.org/fungidb/app/record/gene/HMPREF1120_02639)  *[Exophiala dermatitidis NIH/UT8656]*  *Serine/threonine-protein phosphatase 2A 56 kDa regulatory subunit delta isoform* [*HMPREF1120_01344*](https://fungidb.org/fungidb/app/record/gene/HMPREF1120_01344) *[Exophiala dermatitidis NIH/UT8656]*  *hypothetical protein* [*HMPREF1120_02700*](https://fungidb.org/fungidb/app/record/gene/HMPREF1120_02700) *[Exophiala dermatitidis NIH/UT8656]*  *hypothetical protein* [*HMPREF1120_02708*](https://fungidb.org/fungidb/app/record/gene/HMPREF1120_02708) *[Exophiala dermatitidis NIH/UT8656]*  *hypothetical protein* [*HMPREF1120_02758*](https://fungidb.org/fungidb/app/record/gene/HMPREF1120_02758) *[Exophiala dermatitidis NIH/UT8656]*  *MC family mitochondrial carrier protein* [*HMPREF1120_08788*](https://fungidb.org/fungidb/app/record/gene/HMPREF1120_08788)*[Exophiala dermatitidis NIH/UT8656]*  *hypothetical protein* [*HMPREF1120_02784*](https://fungidb.org/fungidb/app/record/gene/HMPREF1120_02784) *[Exophiala dermatitidis NIH/UT8656]*  *hypothetical protein* [*HMPREF1120_02852*](https://fungidb.org/fungidb/app/record/gene/HMPREF1120_02852) *[Exophiala dermatitidis NIH/UT8656]*  *MOT1 - TATA-binding protein-associated factor* [*HMPREF1120_06808*](https://fungidb.org/fungidb/app/record/gene/HMPREF1120_06808)  *[Exophiala dermatitidis NIH/UT8656]*  *ING3 - Inhibitor of growth protein 3* [*HMPREF1120_05286*](https://fungidb.org/fungidb/app/record/gene/HMPREF1120_05286) *[Exophiala dermatitidis NIH/UT8656]*  *PDE - 3',5'-cyclic-nucleotide phosphodiesterase* [*HMPREF1120_05232*](https://fungidb.org/fungidb/app/record/gene/HMPREF1120_05232) *[Exophiala dermatitidis NIH/UT8656]*  *hypothetical protein* [*HMPREF1120_02947*](https://fungidb.org/fungidb/app/record/gene/HMPREF1120_02947) *[Exophiala dermatitidis NIH/UT8656]*  *hypothetical protein* [*HMPREF1120_03007*](https://fungidb.org/fungidb/app/record/gene/HMPREF1120_03007) *[Exophiala dermatitidis NIH/UT8656]*  *polyketide synthase* [*HMPREF1120_06570*](https://fungidb.org/fungidb/app/record/gene/HMPREF1120_06570)*[Exophiala dermatitidis NIH/UT8656]*  *VEI - velvet protein* [*HMPREF1120_06091*](https://fungidb.org/fungidb/app/record/gene/HMPREF1120_06091)*[Exophiala dermatitidis NIH/UT8656]*  *hypothetical protein* [*HMPREF1120_03359*](https://fungidb.org/fungidb/app/record/gene/HMPREF1120_03359) *[Exophiala dermatitidis NIH/UT8656]*  *salicylate hydroxylase* [*HMPREF1120_03459*](https://fungidb.org/fungidb/app/record/gene/HMPREF1120_03459)*[Exophiala dermatitidis NIH/UT8656]*  *hypothetical protein* [*HMPREF1120_03367*](https://fungidb.org/fungidb/app/record/gene/HMPREF1120_03367) *[Exophiala dermatitidis NIH/UT8656]*  *CPR6 - peptidyl-prolyl cis-trans isomerase cpr6* [*HMPREF1120_08126*](https://fungidb.org/fungidb/app/record/gene/HMPREF1120_08126)*[Exophiala dermatitidis NIH/UT8656]*  *gibberellin 2-oxidase* [*HMPREF1120_09208*](https://fungidb.org/fungidb/app/record/gene/HMPREF1120_09208)*[Exophiala dermatitidis NIH/UT8656]*  *hypothetical protein* [*HMPREF1120_03804*](https://fungidb.org/fungidb/app/record/gene/HMPREF1120_03804) *[Exophiala dermatitidis NIH/UT8656]*  *hypothetical protein* [*HMPREF1120_03909*](https://fungidb.org/fungidb/app/record/gene/HMPREF1120_03909) *[Exophiala dermatitidis NIH/UT8656]*  *hypothetical protein* [*HMPREF1120_03910*](https://fungidb.org/fungidb/app/record/gene/HMPREF1120_03910) *[Exophiala dermatitidis NIH/UT8656]*  *hypothetical protein* [*HMPREF1120_03928*](https://fungidb.org/fungidb/app/record/gene/HMPREF1120_03928) *[Exophiala dermatitidis NIH/UT8656]*  *amidohydrolase* [*HMPREF1120_03964*](https://fungidb.org/fungidb/app/record/gene/HMPREF1120_03964)*[Exophiala dermatitidis NIH/UT8656]*  *hypothetical protein* [*HMPREF1120_04101*](https://fungidb.org/fungidb/app/record/gene/HMPREF1120_04101) *[Exophiala dermatitidis NIH/UT8656]*  *MFS transporter, SP family, sugar:H+ symporter* [*HMPREF1120_06771*](https://fungidb.org/fungidb/app/record/gene/HMPREF1120_06771) *[Exophiala dermatitidis NIH/UT8656]*  *COP9 signalosome complex subunit 2* [*HMPREF1120_04182*](https://fungidb.org/fungidb/app/record/gene/HMPREF1120_04182)*[Exophiala dermatitidis NIH/UT8656]*  *hypothetical protein* [*HMPREF1120_04204*](https://fungidb.org/fungidb/app/record/gene/HMPREF1120_04204) *[Exophiala dermatitidis NIH/UT8656]*  *SIP3 - Putative sterol transfer protein* [*HMPREF1120_04207*](https://fungidb.org/fungidb/app/record/gene/HMPREF1120_04207)*[Exophiala dermatitidis NIH/UT8656]*  *hypothetical protein* [*HMPREF1120_04232*](https://fungidb.org/fungidb/app/record/gene/HMPREF1120_04232) *[Exophiala dermatitidis NIH/UT8656]*  *hypothetical protein* [*HMPREF1120_04314*](https://fungidb.org/fungidb/app/record/gene/HMPREF1120_04314) *[Exophiala dermatitidis NIH/UT8656]*  *hypothetical protein* [*HMPREF1120_04349*](https://fungidb.org/fungidb/app/record/gene/HMPREF1120_04349) *[Exophiala dermatitidis NIH/UT8656]*  *KAR3 - kinesin-like nuclear fusion protein [Exophiala dermatitidis NIH/UT8656]*  *hypothetical protein* [*HMPREF1120_04459*](https://fungidb.org/fungidb/app/record/gene/HMPREF1120_04459) *[Exophiala dermatitidis NIH/UT8656]*  *tyrosinase* [*HMPREF1120_04514*](https://fungidb.org/fungidb/app/record/gene/HMPREF1120_04514)*[Exophiala dermatitidis NIH/UT8656]*  *hypothetical protein* [*HMPREF1120_04584*](https://fungidb.org/fungidb/app/record/gene/HMPREF1120_04584) *[Exophiala dermatitidis NIH/UT8656]*  *ankyrin* [*HMPREF1120_08463*](https://fungidb.org/fungidb/app/record/gene/HMPREF1120_08463)*[Exophiala dermatitidis NIH/UT8656]*  *hypothetical protein* [*HMPREF1120_04645*](https://fungidb.org/fungidb/app/record/gene/HMPREF1120_04645) *[Exophiala dermatitidis NIH/UT8656]*  *hypothetical protein* [*HMPREF1120_04673*](https://fungidb.org/fungidb/app/record/gene/HMPREF1120_04673) *[Exophiala dermatitidis NIH/UT8656]*  *alkanesulfonate monooxygenase* [*HMPREF1120_08264*](https://fungidb.org/fungidb/app/record/gene/HMPREF1120_08264)*[Exophiala dermatitidis NIH/UT8656]*  *hypothetical protein* [*HMPREF1120_04695*](https://fungidb.org/fungidb/app/record/gene/HMPREF1120_04695) *[Exophiala dermatitidis NIH/UT8656]*  *hypothetical protein* [*HMPREF1120_04835*](https://fungidb.org/fungidb/app/record/gene/HMPREF1120_04835) *[Exophiala dermatitidis NIH/UT8656]*  *hypothetical protein* [*HMPREF1120_04991*](https://fungidb.org/fungidb/app/record/gene/HMPREF1120_04991) *[Exophiala dermatitidis NIH/UT8656]*  *4-coumarate-CoA ligase* [*HMPREF1120_09210*](https://fungidb.org/fungidb/app/record/gene/HMPREF1120_09210)*[Exophiala dermatitidis NIH/UT8656]*  *hypothetical protein* [*HMPREF1120_05054*](https://fungidb.org/fungidb/app/record/gene/HMPREF1120_05054) *[Exophiala dermatitidis NIH/UT8656]*  *KU70 - ATP-dependent DNA helicase II subunit 1* [*HMPREF1120_05117*](https://fungidb.org/fungidb/app/record/gene/HMPREF1120_05117)*[Exophiala dermatitidis NIH/UT8656]*  *hypothetical protein* [*HMPREF1120_05153*](https://fungidb.org/fungidb/app/record/gene/HMPREF1120_05153) *[Exophiala dermatitidis NIH/UT8656]*  *hypothetical protein* [*HMPREF1120_05151*](https://fungidb.org/fungidb/app/record/gene/HMPREF1120_05151) *[Exophiala dermatitidis NIH/UT8656]*  *hypothetical protein* [*HMPREF1120_05181*](https://fungidb.org/fungidb/app/record/gene/HMPREF1120_05181) *[Exophiala dermatitidis NIH/UT8656]*  *hypothetical protein* [*HMPREF1120_05217*](https://fungidb.org/fungidb/app/record/gene/HMPREF1120_05217) *[Exophiala dermatitidis NIH/UT8656]*  *dimethylaniline monooxygenase (N-oxide forming)* [*HMPREF1120_08742*](https://fungidb.org/fungidb/app/record/gene/HMPREF1120_08742)*[Exophiala dermatitidis NIH/UT8656]*  *hypothetical protein* [*HMPREF1120_05400*](https://fungidb.org/fungidb/app/record/gene/HMPREF1120_05400) *[Exophiala dermatitidis NIH/UT8656]*  *dihydrodipicolinate synthetase* [*HMPREF1120_09161*](https://fungidb.org/fungidb/app/record/gene/HMPREF1120_09161)*[Exophiala dermatitidis NIH/UT8656]*  *DNA repair protein RAD50* [*HMPREF1120_04505*](https://fungidb.org/fungidb/app/record/gene/HMPREF1120_04505) *[Exophiala dermatitidis NIH/UT8656]*  *hypothetical protein* [*HMPREF1120_05622*](https://fungidb.org/fungidb/app/record/gene/HMPREF1120_05622) *[Exophiala dermatitidis NIH/UT8656]*  *hypothetical protein* [*HMPREF1120_05658*](https://fungidb.org/fungidb/app/record/gene/HMPREF1120_05658) *[Exophiala dermatitidis NIH/UT8656]*  *PSY2 - Platinum sensitivity protein [Exophiala dermatitidis NIH/UT8656]*  *hypothetical protein* [*HMPREF1120_05728*](https://fungidb.org/fungidb/app/record/gene/HMPREF1120_05728) *[Exophiala dermatitidis NIH/UT8656]*  *SPT20 - Transcription factor spt20* [*HMPREF1120_05758*](https://fungidb.org/fungidb/app/record/gene/HMPREF1120_05758)*[Exophiala dermatitidis NIH/UT8656]*  *hypothetical protein* [*HMPREF1120_05809*](https://fungidb.org/fungidb/app/record/gene/HMPREF1120_05809) *[Exophiala dermatitidis NIH/UT8656]*  *ETF1 - elongation factor 2* [*HMPREF1120_05986*](https://fungidb.org/fungidb/app/record/gene/HMPREF1120_05986)*[Exophiala dermatitidis NIH/UT8656]*  *hypothetical protein* [*HMPREF1120_06093*](https://fungidb.org/fungidb/app/record/gene/HMPREF1120_06093) *[Exophiala dermatitidis NIH/UT8656]*  *RGT1 - Glucose-responsive transcription factor 1 [Exophiala dermatitidis NIH/UT8656]*  *hypothetical protein* [*HMPREF1120_06177*](https://fungidb.org/fungidb/app/record/gene/HMPREF1120_06177) *[Exophiala dermatitidis NIH/UT8656]*  *hypothetical protein* [*HMPREF1120_06361*](https://fungidb.org/fungidb/app/record/gene/HMPREF1120_06361) *[Exophiala dermatitidis NIH/UT8656]*  *37S ribosomal protein, mitochondrial [Exophiala dermatitidis NIH/UT8656]*  *hypothetical protein* [*HMPREF1120_06500*](https://fungidb.org/fungidb/app/record/gene/HMPREF1120_06500) *[Exophiala dermatitidis NIH/UT8656]*  *hypothetical protein* [*HMPREF1120_06642*](https://fungidb.org/fungidb/app/record/gene/HMPREF1120_06642) *[Exophiala dermatitidis NIH/UT8656]*  *MFS transporter, SP family, solute carrier family 2 (facilitated glucose transporter), member 2* [*HMPREF1120_06771*](https://fungidb.org/fungidb/app/record/gene/HMPREF1120_06771)  *[Exophiala dermatitidis NIH/UT8656]*  *hypothetical protein* [*HMPREF1120_06878*](https://fungidb.org/fungidb/app/record/gene/HMPREF1120_06878) *[Exophiala dermatitidis NIH/UT8656]*  *hypothetical protein* [*HMPREF1120_06971*](https://fungidb.org/fungidb/app/record/gene/HMPREF1120_06971) *[Exophiala dermatitidis NIH/UT8656]*  *transcription initiation factor TFIID subunit D2* [*MPREF1120_06984*](https://fungidb.org/fungidb/app/record/gene/HMPREF1120_06984)*[Exophiala dermatitidis NIH/UT8656]*  *L-galactose dehydrogenase* [*HMPREF1120_07000*](https://fungidb.org/fungidb/app/record/gene/HMPREF1120_07000)*[Exophiala dermatitidis NIH/UT8656]*  *FAS1_2 - beta subunit of fatty acid synthetase* [*HMPREF1120_07065*](https://fungidb.org/fungidb/app/record/gene/HMPREF1120_07065)*[Exophiala dermatitidis NIH/UT8656]*  *hypothetical protein* [*HMPREF1120_07085*](https://fungidb.org/fungidb/app/record/gene/HMPREF1120_07085) *[Exophiala dermatitidis NIH/UT8656]*  *3' exoribonuclease* [*HMPREF1120_08304*](https://fungidb.org/fungidb/app/record/gene/HMPREF1120_08304)*[Exophiala dermatitidis NIH/UT8656]*  *hypothetical protein* [*HMPREF1120_07255*](https://fungidb.org/fungidb/app/record/gene/HMPREF1120_07255) *[Exophiala dermatitidis NIH/UT8656]*  *hypothetical protein* [*HMPREF1120_07291*](https://fungidb.org/fungidb/app/record/gene/HMPREF1120_07291) *[Exophiala dermatitidis NIH/UT8656]*  *hypothetical protein* [*HMPREF1120_07306*](https://fungidb.org/fungidb/app/record/gene/HMPREF1120_07306) *[Exophiala dermatitidis NIH/UT8656]*  *hypothetical protein* [*HMPREF1120_07433*](https://fungidb.org/fungidb/app/record/gene/HMPREF1120_07433) *[Exophiala dermatitidis NIH/UT8656]*  *RAS2 - Ras GTPase* [*HMPREF1120_01421*](https://fungidb.org/fungidb/app/record/gene/HMPREF1120_01421)*[Exophiala dermatitidis NIH/UT8656]*  *hypothetical protein* [*HMPREF1120_07616*](https://fungidb.org/fungidb/app/record/gene/HMPREF1120_07616) *[Exophiala dermatitidis NIH/UT8656]*  *D-3-phosphoglycerate dehydrogenase* [*HMPREF1120_06805*](https://fungidb.org/fungidb/app/record/gene/HMPREF1120_06805)*[Exophiala dermatitidis NIH/UT8656]*  *ankyrin* [*HMPREF1120_08463*](https://fungidb.org/fungidb/app/record/gene/HMPREF1120_08463)*[Exophiala dermatitidis NIH/UT8656]*  *nuclear transcription factor Y, alpha* [*HMPREF1120_07714*](https://fungidb.org/fungidb/app/record/gene/HMPREF1120_07714)*[Exophiala dermatitidis NIH/UT8656]*  *CHS3 - chitin synthase class 3 [Exophiala dermatitidis NIH/UT8656]*  *salicylate hydroxylase* [*HMPREF1120_03459*](https://fungidb.org/fungidb/app/record/gene/HMPREF1120_03459)*[Exophiala dermatitidis NIH/UT8656]*  *hypothetical protein* [*HMPREF1120_06589*](https://fungidb.org/fungidb/app/record/gene/HMPREF1120_06589) *[Exophiala dermatitidis NIH/UT8656]*  *hypothetical protein* [*HMPREF1120_06584*](https://fungidb.org/fungidb/app/record/gene/HMPREF1120_06584) *[Exophiala dermatitidis NIH/UT8656]*  *queuine tRNA-ribosyltransferase* [*HMPREF1120_07977*](https://fungidb.org/fungidb/app/record/gene/HMPREF1120_07977)*[Exophiala dermatitidis NIH/UT8656]*  *hypothetical protein* [*HMPREF1120_07985*](https://fungidb.org/fungidb/app/record/gene/HMPREF1120_07985) *[Exophiala dermatitidis NIH/UT8656]*  *thiamin biosynthesis protein* [*HMPREF1120_07987*](https://fungidb.org/fungidb/app/record/gene/HMPREF1120_07987)*[Exophiala dermatitidis NIH/UT8656]*  *ABD1 - mRNA cap guanine-N7 methyltransferase* [*HMPREF1120_06541*](https://fungidb.org/fungidb/app/record/gene/HMPREF1120_06541)*[Exophiala dermatitidis NIH/UT8656]*  *hypothetical protein* [*HMPREF1120_08063*](https://fungidb.org/fungidb/app/record/gene/HMPREF1120_08063) *[Exophiala dermatitidis NIH/UT8656]*  *PAN2 - poly(A) specific ribonuclease [Exophiala dermatitidis NIH/UT8656]*  *DOA4 - ubiquitin specific protease* [*HMPREF1120_06573*](https://fungidb.org/fungidb/app/record/gene/HMPREF1120_06573)*[Exophiala dermatitidis NIH/UT8656]*  *amidase* [*HMPREF1120_09153*](https://fungidb.org/fungidb/app/record/gene/HMPREF1120_09153)*[Exophiala dermatitidis NIH/UT8656]*  *MEF2 - Ribosome-releasing factor 2, mitochondrial [Exophiala dermatitidis NIH/UT8656]*  *RGA2 - Rho-type gtpase-activating protein [Exophiala dermatitidis NIH/UT8656]*  *hypothetical protein* [*HMPREF1120_08236*](https://fungidb.org/fungidb/app/record/gene/HMPREF1120_08236) *[Exophiala dermatitidis NIH/UT8656]*  *hypothetical protein* [*HMPREF1120_08238*](https://fungidb.org/fungidb/app/record/gene/HMPREF1120_08238) *[Exophiala dermatitidis NIH/UT8656]*  *hypothetical protein* [*HMPREF1120_08408*](https://fungidb.org/fungidb/app/record/gene/HMPREF1120_08408) *[Exophiala dermatitidis NIH/UT8656]*  *cytochrome P450 oxidoreductase* [*HMPREF1120_01361*](https://fungidb.org/fungidb/app/record/gene/HMPREF1120_01361) *[Exophiala dermatitidis NIH/UT8656]*  *hypothetical protein* [*HMPREF1120_08425*](https://fungidb.org/fungidb/app/record/gene/HMPREF1120_08425) *[Exophiala dermatitidis NIH/UT8656]*  *hydrolase* [*HMPREF1120_08460*](https://fungidb.org/fungidb/app/record/gene/HMPREF1120_08460)*[Exophiala dermatitidis NIH/UT8656]*  *hypothetical protein* [*HMPREF1120_08629*](https://fungidb.org/fungidb/app/record/gene/HMPREF1120_08629) *[Exophiala dermatitidis NIH/UT8656]*  *pyruvate carboxylase* [*HMPREF1120_09185*](https://fungidb.org/fungidb/app/record/gene/HMPREF1120_08629) *[Exophiala dermatitidis NIH/UT8656]*  *branchpoint-bridging protein* [*HMPREF1120_08884*](https://fungidb.org/fungidb/app/record/gene/HMPREF1120_08884)*[Exophiala dermatitidis NIH/UT8656]*  *hypothetical protein* [*HMPREF1120_08890*](https://fungidb.org/fungidb/app/record/gene/HMPREF1120_08890) *[Exophiala dermatitidis NIH/UT8656]*  *hypothetical protein* [*HMPREF1120_09031*](https://fungidb.org/fungidb/app/record/gene/HMPREF1120_09031) *[Exophiala dermatitidis NIH/UT8656]*  *hypothetical protein* [*HMPREF1120_09084*](https://fungidb.org/fungidb/app/record/gene/HMPREF1120_09084) *[Exophiala dermatitidis NIH/UT8656]*  *chitin synthase* [*HMPREF1120_08777*](https://fungidb.org/fungidb/app/record/gene/HMPREF1120_08777)  *[Exophiala dermatitidis NIH/UT8656]*  *hypothetical protein* [*HMPREF1120_09220*](https://fungidb.org/fungidb/app/record/gene/HMPREF1120_09220) *[Exophiala dermatitidis NIH/UT8656]*  *ribonuclease H2 subunit A* [*HMPREF1120_09244*](https://fungidb.org/fungidb/app/record/gene/HMPREF1120_09244) *[Exophiala dermatitidis NIH/UT8656]* |  |
| *Ex5 & Ex20*  *111* | *DNA repair protein RAD50* [*HMPREF1120_04505*](https://fungidb.org/fungidb/app/record/gene/HMPREF1120_04505) *[Exophiala dermatitidis NIH/UT8656]*  *hypothetical protein* [*HMPREF1120_00144*](https://fungidb.org/fungidb/app/record/gene/HMPREF1120_00144) *[Exophiala dermatitidis NIH/UT8656]*  *hypothetical protein* [*HMPREF1120_00457*](https://fungidb.org/fungidb/app/record/gene/HMPREF1120_00457) *[Exophiala dermatitidis NIH/UT8656]*  *hypothetical protein* [*HMPREF1120_00646*](https://fungidb.org/fungidb/app/record/gene/HMPREF1120_00646) *[Exophiala dermatitidis NIH/UT8656]*  *eukaryotic translation initiation factor 2 subunit gamma* [*HMPREF1120_00677*](https://fungidb.org/fungidb/app/record/gene/HMPREF1120_00677)  *[Exophiala dermatitidis NIH/UT8656]*  *cytochrome P450 oxidoreductase* [*HMPREF1120_01361*](https://fungidb.org/fungidb/app/record/gene/HMPREF1120_01361) *[Exophiala dermatitidis NIH/UT8656]*  *hypothetical protein* [*HMPREF1120_00830*](https://fungidb.org/fungidb/app/record/gene/HMPREF1120_00830) *[Exophiala dermatitidis NIH/UT8656]*  *hypothetical protein* [*HMPREF1120_01089*](https://fungidb.org/fungidb/app/record/gene/HMPREF1120_01089) *[Exophiala dermatitidis NIH/UT8656]*  *hypothetical protein* [*HMPREF1120_01151*](https://fungidb.org/fungidb/app/record/gene/HMPREF1120_01151) *[Exophiala dermatitidis NIH/UT8656]*  *prolyl-tRNA synthetase* [*HMPREF1120_01354*](https://fungidb.org/fungidb/app/record/gene/HMPREF1120_01354) *[Exophiala dermatitidis NIH/UT8656]*  *hypothetical protein* [*HMPREF1120_01471*](https://fungidb.org/fungidb/app/record/gene/HMPREF1120_01471) *[Exophiala dermatitidis NIH/UT8656]*  *serine/threonine kinase 16* [*HMPREF1120_06920*](https://fungidb.org/fungidb/app/record/gene/HMPREF1120_06920) *[Exophiala dermatitidis NIH/UT8656]*  *hypothetical protein* [*HMPREF1120_02083*](https://fungidb.org/fungidb/app/record/gene/HMPREF1120_02083) *[Exophiala dermatitidis NIH/UT8656]*  *hypothetical protein* [*HMPREF1120_02190*](https://fungidb.org/fungidb/app/record/gene/HMPREF1120_02190) *[Exophiala dermatitidis NIH/UT8656]*  *glutathione S-transferase* [*HMPREF1120_08143*](https://fungidb.org/fungidb/app/record/gene/HMPREF1120_08143) *[Exophiala dermatitidis NIH/UT8656]*  *nicotinate-nucleotide diphosphorylase (carboxylating)* [*HMPREF1120_02317*](https://fungidb.org/fungidb/app/record/gene/HMPREF1120_02317)  *[Exophiala dermatitidis NIH/UT8656]*  *phosphodiesterase/alkaline phosphatase D* [*HMPREF1120_02364*](https://fungidb.org/fungidb/app/record/gene/HMPREF1120_02364) *[Exophiala dermatitidis NIH/UT8656]*  *Xanthine phosphoribosyltransferase 1* [*HMPREF1120_06110*](https://fungidb.org/fungidb/app/record/gene/HMPREF1120_06110) *[Exophiala dermatitidis NIH/UT8656]*  *hypothetical protein* [*HMPREF1120_02700*](https://fungidb.org/fungidb/app/record/gene/HMPREF1120_02700) *[Exophiala dermatitidis NIH/UT8656]*  *hypothetical protein* [*HMPREF1120_02708*](https://fungidb.org/fungidb/app/record/gene/HMPREF1120_02708) *[Exophiala dermatitidis NIH/UT8656]*  *hypothetical protein* [*HMPREF1120_02758*](https://fungidb.org/fungidb/app/record/gene/HMPREF1120_02758) *[Exophiala dermatitidis NIH/UT8656]*  *hypothetical protein* [*HMPREF1120_02784*](https://fungidb.org/fungidb/app/record/gene/HMPREF1120_02784) *[Exophiala dermatitidis NIH/UT8656]*  *MOT1 - TATA-binding protein-associated factor* [*HMPREF1120_06808*](https://fungidb.org/fungidb/app/record/gene/HMPREF1120_06808)  *[Exophiala dermatitidis NIH/UT8656]*  *ING3 - Inhibitor of growth protein 3* [*HMPREF1120_05286*](https://fungidb.org/fungidb/app/record/gene/HMPREF1120_05286) *[Exophiala dermatitidis NIH/UT8656]*  *hypothetical protein* [*HMPREF1120_02908*](https://fungidb.org/fungidb/app/record/gene/HMPREF1120_02908) *[Exophiala dermatitidis NIH/UT8656]*  *PDE2 - High-affinity cyclic AMP phosphodiesterase [Exophiala dermatitidis NIH/UT8656]*  *hypothetical protein* [*HMPREF1120_03003*](https://fungidb.org/fungidb/app/record/gene/HMPREF1120_03003) *[Exophiala dermatitidis NIH/UT8656]*  *hypothetical protein* [*HMPREF1120_03007*](https://fungidb.org/fungidb/app/record/gene/HMPREF1120_03007) *[Exophiala dermatitidis NIH/UT8656]*  *VEI - velvet protein* [*HMPREF1120_06091*](https://fungidb.org/fungidb/app/record/gene/HMPREF1120_06091)*[Exophiala dermatitidis NIH/UT8656]*  *hypothetical protein* [*HMPREF1120_03359*](https://fungidb.org/fungidb/app/record/gene/HMPREF1120_03359) *[Exophiala dermatitidis NIH/UT8656]*  *hypothetical protein* [*HMPREF1120_03513*](https://fungidb.org/fungidb/app/record/gene/HMPREF1120_03513) *[Exophiala dermatitidis NIH/UT8656]*  *gibberellin 2-oxidase* [*HMPREF1120_09208*](https://fungidb.org/fungidb/app/record/gene/HMPREF1120_09208)*[Exophiala dermatitidis NIH/UT8656]*  *hypothetical protein* [*HMPREF1120_03910*](https://fungidb.org/fungidb/app/record/gene/HMPREF1120_03910) *[Exophiala dermatitidis NIH/UT8656]*  *hypothetical protein* [*HMPREF1120_03911*](https://fungidb.org/fungidb/app/record/gene/HMPREF1120_03911) *[Exophiala dermatitidis NIH/UT8656]*  *hypothetical protein* [*HMPREF1120_03928*](https://fungidb.org/fungidb/app/record/gene/HMPREF1120_03928) *[Exophiala dermatitidis NIH/UT8656]*  *amidohydrolase* [*HMPREF1120_03964*](https://fungidb.org/fungidb/app/record/gene/HMPREF1120_03964) *[Exophiala dermatitidis NIH/UT8656]*  *hypothetical protein* [*HMPREF1120_04101*](https://fungidb.org/fungidb/app/record/gene/HMPREF1120_04101) *[Exophiala dermatitidis NIH/UT8656]*  *MFS transporter, SP family, sugar:H+ symporter* [*HMPREF1120_06771*](https://fungidb.org/fungidb/app/record/gene/HMPREF1120_06771)  *[Exophiala dermatitidis NIH/UT8656]*  *COP9 signalosome complex subunit 2* [*HMPREF1120_04182*](https://fungidb.org/fungidb/app/record/gene/HMPREF1120_04182)*[Exophiala dermatitidis NIH/UT8656]*  *hypothetical protein* [*HMPREF1120_04204*](https://fungidb.org/fungidb/app/record/gene/HMPREF1120_04204) *[Exophiala dermatitidis NIH/UT8656]*  *SIP3 - Putative sterol transfer protein* [*HMPREF1120_04207*](https://fungidb.org/fungidb/app/record/gene/HMPREF1120_04207)*[Exophiala dermatitidis NIH/UT8656]*  *hypothetical protein* [*HMPREF1120_04349*](https://fungidb.org/fungidb/app/record/gene/HMPREF1120_04349) *[Exophiala dermatitidis NIH/UT8656]*  *hypothetical protein* [*HMPREF1120_04459*](https://fungidb.org/fungidb/app/record/gene/HMPREF1120_04459) *[Exophiala dermatitidis NIH/UT8656]*  *tyrosinase* [*HMPREF1120_04514*](https://fungidb.org/fungidb/app/record/gene/HMPREF1120_04514)*[Exophiala dermatitidis NIH/UT8656]*  *hypothetical protein* [*HMPREF1120_04584*](https://fungidb.org/fungidb/app/record/gene/HMPREF1120_04584) *[Exophiala dermatitidis NIH/UT8656]*  *ankyrin* [*HMPREF1120_08463*](https://fungidb.org/fungidb/app/record/gene/HMPREF1120_08463) *[Exophiala dermatitidis NIH/UT8656]*  *hypothetical protein* [*HMPREF1120_04645*](https://fungidb.org/fungidb/app/record/gene/HMPREF1120_04645) *[Exophiala dermatitidis NIH/UT8656]*  *adenosinetriphosphatase* [*HMPREF1120_09246*](https://fungidb.org/fungidb/app/record/gene/HMPREF1120_09246) *[Exophiala dermatitidis NIH/UT8656]*  *hypothetical protein* [*HMPREF1120_04673*](https://fungidb.org/fungidb/app/record/gene/HMPREF1120_04673) *[Exophiala dermatitidis NIH/UT8656]*  *alkanesulfonate monooxygenase* [*HMPREF1120_08264*](https://fungidb.org/fungidb/app/record/gene/HMPREF1120_08264)*[Exophiala dermatitidis NIH/UT8656]*  *hypothetical protein* [*HMPREF1120_04835*](https://fungidb.org/fungidb/app/record/gene/HMPREF1120_04835) *[Exophiala dermatitidis NIH/UT8656]*  *hypothetical protein* [*HMPREF1120_04991*](https://fungidb.org/fungidb/app/record/gene/HMPREF1120_04991) *[Exophiala dermatitidis NIH/UT8656]*  *4-coumarate-CoA ligase* [*HMPREF1120_09210*](https://fungidb.org/fungidb/app/record/gene/HMPREF1120_09210)*[Exophiala dermatitidis NIH/UT8656]*  *hypothetical protein* [*HMPREF1120_05087*](https://fungidb.org/fungidb/app/record/gene/HMPREF1120_05087) *[Exophiala dermatitidis NIH/UT8656]*  *hypothetical protein* [*HMPREF1120_05151*](https://fungidb.org/fungidb/app/record/gene/HMPREF1120_05151) *[Exophiala dermatitidis NIH/UT8656]*  *hypothetical protein* [*HMPREF1120_05217*](https://fungidb.org/fungidb/app/record/gene/HMPREF1120_05217) *[Exophiala dermatitidis NIH/UT8656]*  *dimethylaniline monooxygenase (N-oxide forming)* [*HMPREF1120_08742*](https://fungidb.org/fungidb/app/record/gene/HMPREF1120_08742)*[Exophiala dermatitidis NIH/UT8656]*  *hypothetical protein* [*HMPREF1120_05393*](https://fungidb.org/fungidb/app/record/gene/HMPREF1120_05393) *[Exophiala dermatitidis NIH/UT8656]*  *hypothetical protein* [*HMPREF1120_05400*](https://fungidb.org/fungidb/app/record/gene/HMPREF1120_05400) *[Exophiala dermatitidis NIH/UT8656]*  *dihydrodipicolinate synthetase* [*HMPREF1120_09161*](https://fungidb.org/fungidb/app/record/gene/HMPREF1120_09161)*[Exophiala dermatitidis NIH/UT8656]*  *hypothetical protein* [*HMPREF1120_05622*](https://fungidb.org/fungidb/app/record/gene/HMPREF1120_05622) *[Exophiala dermatitidis NIH/UT8656]*  *hypothetical protein* [*HMPREF1120_05658*](https://fungidb.org/fungidb/app/record/gene/HMPREF1120_05658) *[Exophiala dermatitidis NIH/UT8656]*  *PSY2 - platinum sensitivity protein [Exophiala dermatitidis NIH/UT8656]*  *hypothetical protein* [*HMPREF1120_05728*](https://fungidb.org/fungidb/app/record/gene/HMPREF1120_05728) *[Exophiala dermatitidis NIH/UT8656]*  *SPT20 - Transcription factor spt20* [*HMPREF1120_05758*](https://fungidb.org/fungidb/app/record/gene/HMPREF1120_05758)*[Exophiala dermatitidis NIH/UT8656]*  *hypothetical protein* [*HMPREF1120_05809*](https://fungidb.org/fungidb/app/record/gene/HMPREF1120_05809) *[Exophiala dermatitidis NIH/UT8656]*  *ETF1 - elongation factor 2* [*HMPREF1120_05986*](https://fungidb.org/fungidb/app/record/gene/HMPREF1120_05986)*[Exophiala dermatitidis NIH/UT8656]*  *hypothetical protein* [*HMPREF1120_06093*](https://fungidb.org/fungidb/app/record/gene/HMPREF1120_06093) *[Exophiala dermatitidis NIH/UT8656]*  *hypothetical protein* [*HMPREF1120_06177*](https://fungidb.org/fungidb/app/record/gene/HMPREF1120_06177) *[Exophiala dermatitidis NIH/UT8656]*  *hypothetical protein* [*HMPREF1120_06361*](https://fungidb.org/fungidb/app/record/gene/HMPREF1120_06361) *[Exophiala dermatitidis NIH/UT8656]*  *37S ribosomal protein, mitochondrial [Exophiala dermatitidis NIH/UT8656]*  *hypothetical protein* [*HMPREF1120_06500*](https://fungidb.org/fungidb/app/record/gene/HMPREF1120_06500) *[Exophiala dermatitidis NIH/UT8656]*  *hypothetical protein* [*HMPREF1120_06642*](https://fungidb.org/fungidb/app/record/gene/HMPREF1120_06642) *[Exophiala dermatitidis NIH/UT8656]*  *hypothetical protein* [*HMPREF1120_06971*](https://fungidb.org/fungidb/app/record/gene/HMPREF1120_06971) *[Exophiala dermatitidis NIH/UT8656]*  *transcription initiation factor TFIID subunit D2* [*MPREF1120_06984*](https://fungidb.org/fungidb/app/record/gene/HMPREF1120_06984)*[Exophiala dermatitidis NIH/UT8656]*  *L-galactose dehydrogenase* [*HMPREF1120_07000*](https://fungidb.org/fungidb/app/record/gene/HMPREF1120_07000)*[Exophiala dermatitidis NIH/UT8656]*  *hypothetical protein* [*HMPREF1120_07085*](https://fungidb.org/fungidb/app/record/gene/HMPREF1120_07085) *[Exophiala dermatitidis NIH/UT8656]*  *3' exoribonuclease* [*HMPREF1120_08304*](https://fungidb.org/fungidb/app/record/gene/HMPREF1120_08304)*[Exophiala dermatitidis NIH/UT8656]*  *hypothetical protein* [*HMPREF1120_07255*](https://fungidb.org/fungidb/app/record/gene/HMPREF1120_07255) *[Exophiala dermatitidis NIH/UT8656]*  *hypothetical protein* [*HMPREF1120_07291*](https://fungidb.org/fungidb/app/record/gene/HMPREF1120_07291) *[Exophiala dermatitidis NIH/UT8656]*  *hypothetical protein* [*HMPREF1120_07306*](https://fungidb.org/fungidb/app/record/gene/HMPREF1120_07306) *[Exophiala dermatitidis NIH/UT8656]*  *hypothetical protein* [*HMPREF1120_07433*](https://fungidb.org/fungidb/app/record/gene/HMPREF1120_07433) *[Exophiala dermatitidis NIH/UT8656]*  *RAS2 - Ras GTPase* [*HMPREF1120_01421*](https://fungidb.org/fungidb/app/record/gene/HMPREF1120_01421)*[Exophiala dermatitidis NIH/UT8656]*  *hypothetical protein* [*HMPREF1120_07616*](https://fungidb.org/fungidb/app/record/gene/HMPREF1120_07616) *[Exophiala dermatitidis NIH/UT8656]*  *D-3-phosphoglycerate dehydrogenase* [*HMPREF1120_06805*](https://fungidb.org/fungidb/app/record/gene/HMPREF1120_06805)*[Exophiala dermatitidis NIH/UT8656]*  *ankyrin* [*HMPREF1120_08463*](https://fungidb.org/fungidb/app/record/gene/HMPREF1120_08463)*[Exophiala dermatitidis NIH/UT8656]*  *nuclear transcription factor Y, alpha* [*HMPREF1120_07714*](https://fungidb.org/fungidb/app/record/gene/HMPREF1120_07714)*[Exophiala dermatitidis NIH/UT8656]*  *salicylate hydroxylase* [*HMPREF1120_03459*](https://fungidb.org/fungidb/app/record/gene/HMPREF1120_03459)*[Exophiala dermatitidis NIH/UT8656]*  *hypothetical protein* [*HMPREF1120_06584*](https://fungidb.org/fungidb/app/record/gene/HMPREF1120_06584) *[Exophiala dermatitidis NIH/UT8656]*  *queuine tRNA-ribosyltransferase* [*HMPREF1120_07977*](https://fungidb.org/fungidb/app/record/gene/HMPREF1120_07977)*[Exophiala dermatitidis NIH/UT8656]*  *hypothetical protein* [*HMPREF1120_07985*](https://fungidb.org/fungidb/app/record/gene/HMPREF1120_07985) *[Exophiala dermatitidis NIH/UT8656]*  *thiamin biosynthesis protein* [*HMPREF1120_07987*](https://fungidb.org/fungidb/app/record/gene/HMPREF1120_07987)*[Exophiala dermatitidis NIH/UT8656]*  *ABD1 - mRNA cap guanine-N7 methyltransferase* [*HMPREF1120_06541*](https://fungidb.org/fungidb/app/record/gene/HMPREF1120_06541)*[Exophiala dermatitidis NIH/UT8656]*  *PAN2 - poly(A) specific ribonuclease [Exophiala dermatitidis NIH/UT8656]*  *DOA4 - ubiquitin specific protease* [*HMPREF1120_06573*](https://fungidb.org/fungidb/app/record/gene/HMPREF1120_06573)*[Exophiala dermatitidis NIH/UT8656]*  *VPS27 - vacuolar protein sorting associated protein 27 [Exophiala dermatitidis NIH/UT8656]*  *MEF2 - Ribosome-releasing factor 2, mitochondrial [Exophiala dermatitidis NIH/UT8656]*  *RGA2 - Rho-type gtpase-activating protein [Exophiala dermatitidis NIH/UT8656]*  *hypothetical protein* [*HMPREF1120_08236*](https://fungidb.org/fungidb/app/record/gene/HMPREF1120_08236) *[Exophiala dermatitidis NIH/UT8656]*  *cytochrome P450 oxidoreductase* [*HMPREF1120_01361*](https://fungidb.org/fungidb/app/record/gene/HMPREF1120_01361) *[Exophiala dermatitidis NIH/UT8656]*  *hypothetical protein* [*HMPREF1120_08425*](https://fungidb.org/fungidb/app/record/gene/HMPREF1120_08425) *[Exophiala dermatitidis NIH/UT8656]*  *hydrolase* [*HMPREF1120_08460*](https://fungidb.org/fungidb/app/record/gene/HMPREF1120_08460)*[Exophiala dermatitidis NIH/UT8656]*  *hypothetical protein* [*HMPREF1120_08629*](https://fungidb.org/fungidb/app/record/gene/HMPREF1120_08629) *[Exophiala dermatitidis NIH/UT8656]*  *pyruvate carboxylase* [*HMPREF1120_09185*](https://fungidb.org/fungidb/app/record/gene/HMPREF1120_09185) *[Exophiala dermatitidis NIH/UT8656]*  *hypothetical protein* [*HMPREF1120_08890*](https://fungidb.org/fungidb/app/record/gene/HMPREF1120_08890) *[Exophiala dermatitidis NIH/UT8656]*  *hypothetical protein* [*HMPREF1120_09084*](https://fungidb.org/fungidb/app/record/gene/HMPREF1120_09084) *[Exophiala dermatitidis NIH/UT8656]*  *chitin synthase* [*HMPREF1120_08777*](https://fungidb.org/fungidb/app/record/gene/HMPREF1120_08777) *[Exophiala dermatitidis NIH/UT8656]*  *hypothetical protein* [*HMPREF1120_09220*](https://fungidb.org/fungidb/app/record/gene/HMPREF1120_09220) *[Exophiala dermatitidis NIH/UT8656]*  *ribonuclease H2 subunit A* [*HMPREF1120_09244*](https://fungidb.org/fungidb/app/record/gene/HMPREF1120_09244)*[Exophiala dermatitidis NIH/UT8656]* |  |
| *Ex5 & Ex21*  *72* | *hypothetical protein* [*HMPREF1120_00144*](https://fungidb.org/fungidb/app/record/gene/HMPREF1120_00144) *[Exophiala dermatitidis NIH/UT8656]*  *hypothetical protein* [*HMPREF1120_00457*](https://fungidb.org/fungidb/app/record/gene/HMPREF1120_00457) *[Exophiala dermatitidis NIH/UT8656]*  *hypothetical protein* [*HMPREF1120_00646*](https://fungidb.org/fungidb/app/record/gene/HMPREF1120_00646) *[Exophiala dermatitidis NIH/UT8656]*  *eukaryotic translation initiation factor 2 subunit gamma* [*HMPREF1120_00677*](https://fungidb.org/fungidb/app/record/gene/HMPREF1120_00677)  *[Exophiala dermatitidis NIH/UT8656]*  *cytochrome P450 oxidoreductase* [*HMPREF1120_01361*](https://fungidb.org/fungidb/app/record/gene/HMPREF1120_01361) *[Exophiala dermatitidis NIH/UT8656]*  *H(+)-transporting V1 sector ATPase subunit H* [*HMPREF1120_00992*](https://fungidb.org/fungidb/app/record/gene/HMPREF1120_00992)*[Exophiala dermatitidis NIH/UT8656]*  *hypothetical protein* [*HMPREF1120_01089*](https://fungidb.org/fungidb/app/record/gene/HMPREF1120_01089) *[Exophiala dermatitidis NIH/UT8656]*  *sulfite oxidase* [*HMPREF1120_05227*](https://fungidb.org/fungidb/app/record/gene/HMPREF1120_05227) *[Exophiala dermatitidis NIH/UT8656]*  *prolyl-tRNA synthetase* [*HMPREF1120_01354*](https://fungidb.org/fungidb/app/record/gene/HMPREF1120_01354) *[Exophiala dermatitidis NIH/UT8656]*  *cytochrome P450 oxidoreductase* [*HMPREF1120_01361*](https://fungidb.org/fungidb/app/record/gene/HMPREF1120_01361) *[Exophiala dermatitidis NIH/UT8656]*  *hypothetical protein* [*HMPREF1120_01814*](https://fungidb.org/fungidb/app/record/gene/HMPREF1120_01814) *[Exophiala dermatitidis NIH/UT8656]*  *serine/threonine kinase 16* [*HMPREF1120_06920*](https://fungidb.org/fungidb/app/record/gene/HMPREF1120_06920) *[Exophiala dermatitidis NIH/UT8656]*  *G4 quadruplex nucleic acid binding protein [Exophiala dermatitidis NIH/UT8656]*  *hypothetical protein* [*HMPREF1120_02190*](https://fungidb.org/fungidb/app/record/gene/HMPREF1120_02190) *[Exophiala dermatitidis NIH/UT8656]*  *nicotinate-nucleotide diphosphorylase (carboxylating)* [*HMPREF1120_02317*](https://fungidb.org/fungidb/app/record/gene/HMPREF1120_02317)  *[Exophiala dermatitidis NIH/UT8656]*  *phosphodiesterase/alkaline phosphatase D* [*HMPREF1120_02364*](https://fungidb.org/fungidb/app/record/gene/HMPREF1120_02364) *[Exophiala dermatitidis NIH/UT8656]*  *Xanthine phosphoribosyltransferase 1* [*HMPREF1120_06110*](https://fungidb.org/fungidb/app/record/gene/HMPREF1120_06110) *[Exophiala dermatitidis NIH/UT8656]*  *Serine/threonine-protein phosphatase 2A 56 kDa regulatory subunit delta isoform* [*HMPREF1120_01344*](https://fungidb.org/fungidb/app/record/gene/HMPREF1120_01344) *[Exophiala dermatitidis NIH/UT8656]*  *hypothetical protein* [*HMPREF1120_02784*](https://fungidb.org/fungidb/app/record/gene/HMPREF1120_02784) *[Exophiala dermatitidis NIH/UT8656]*  *ING3 - Inhibitor of growth protein 3* [*HMPREF1120_05286*](https://fungidb.org/fungidb/app/record/gene/HMPREF1120_05286) *[Exophiala dermatitidis NIH/UT8656]*  *hypothetical protein* [*HMPREF1120_02908*](https://fungidb.org/fungidb/app/record/gene/HMPREF1120_02908) *[Exophiala dermatitidis NIH/UT8656]*  *PDE2 - High-affinity cyclic AMP phosphodiesterase [Exophiala dermatitidis NIH/UT8656]*  *gibberellin 2-oxidase* [*HMPREF1120_09208*](https://fungidb.org/fungidb/app/record/gene/HMPREF1120_09208)*[Exophiala dermatitidis NIH/UT8656]*  *hypothetical protein* [*HMPREF1120_04101*](https://fungidb.org/fungidb/app/record/gene/HMPREF1120_04101) *[Exophiala dermatitidis NIH/UT8656]*  *MFS transporter, SP family, sugar:H+ symporter* [*HMPREF1120_06771*](https://fungidb.org/fungidb/app/record/gene/HMPREF1120_06771) *[Exophiala dermatitidis NIH/UT8656]*  *SIP3 - Putative sterol transfer protein* [*HMPREF1120_04207*](https://fungidb.org/fungidb/app/record/gene/HMPREF1120_04207)*[Exophiala dermatitidis NIH/UT8656]*  *hypothetical protein* [*HMPREF1120_04349*](https://fungidb.org/fungidb/app/record/gene/HMPREF1120_04349) *[Exophiala dermatitidis NIH/UT8656]*  *hypothetical protein* [*HMPREF1120_04459*](https://fungidb.org/fungidb/app/record/gene/HMPREF1120_04459) *[Exophiala dermatitidis NIH/UT8656]*  *ankyrin* [*HMPREF1120_08463*](https://fungidb.org/fungidb/app/record/gene/HMPREF1120_08463)*[Exophiala dermatitidis NIH/UT8656]*  *hypothetical protein* [*HMPREF1120_04991*](https://fungidb.org/fungidb/app/record/gene/HMPREF1120_04991) *[Exophiala dermatitidis NIH/UT8656]*  *arginyl-tRNA synthetase* [*HMPREF1120_08595*](https://fungidb.org/fungidb/app/record/gene/HMPREF1120_08595)*[Exophiala dermatitidis NIH/UT8656]*  *hypothetical protein* [*HMPREF1120_05087*](https://fungidb.org/fungidb/app/record/gene/HMPREF1120_05087) *[Exophiala dermatitidis NIH/UT8656]*  *hypothetical protein* [*HMPREF1120_05151*](https://fungidb.org/fungidb/app/record/gene/HMPREF1120_05151) *[Exophiala dermatitidis NIH/UT8656]*  *hypothetical protein* [*HMPREF1120_05217*](https://fungidb.org/fungidb/app/record/gene/HMPREF1120_05217) *[Exophiala dermatitidis NIH/UT8656]*  *dihydrodipicolinate synthetase* [*HMPREF1120_09161*](https://fungidb.org/fungidb/app/record/gene/HMPREF1120_09161)*[Exophiala dermatitidis NIH/UT8656]*  *hypothetical protein* [*HMPREF1120_05658*](https://fungidb.org/fungidb/app/record/gene/HMPREF1120_05658) *[Exophiala dermatitidis NIH/UT8656]*  *ETF1 - elongation factor 2* [*HMPREF1120_05986*](https://fungidb.org/fungidb/app/record/gene/HMPREF1120_05986)*[Exophiala dermatitidis NIH/UT8656]*  *hypothetical protein* [*HMPREF1120_06093*](https://fungidb.org/fungidb/app/record/gene/HMPREF1120_06093) *[Exophiala dermatitidis NIH/UT8656]*  *hypothetical protein* [*HMPREF1120_06177*](https://fungidb.org/fungidb/app/record/gene/HMPREF1120_06177) *[Exophiala dermatitidis NIH/UT8656]*  *hypothetical protein* [*HMPREF1120_06361*](https://fungidb.org/fungidb/app/record/gene/HMPREF1120_06361) *[Exophiala dermatitidis NIH/UT8656]*  *hypothetical protein* [*HMPREF1120_06500*](https://fungidb.org/fungidb/app/record/gene/HMPREF1120_06500) *[Exophiala dermatitidis NIH/UT8656]*  *hypothetical protein* [*HMPREF1120_06642*](https://fungidb.org/fungidb/app/record/gene/HMPREF1120_06642) *[Exophiala dermatitidis NIH/UT8656]*  *hypothetical protein* [*HMPREF1120_06852*](https://fungidb.org/fungidb/app/record/gene/HMPREF1120_06852) *[Exophiala dermatitidis NIH/UT8656]*  *transcription initiation factor TFIID subunit D2* [*MPREF1120_06984*](https://fungidb.org/fungidb/app/record/gene/HMPREF1120_06984)*[Exophiala dermatitidis NIH/UT8656]*  *L-galactose dehydrogenase* [*HMPREF1120_07000*](https://fungidb.org/fungidb/app/record/gene/HMPREF1120_07000)*[Exophiala dermatitidis NIH/UT8656]*  *hypothetical protein* [*HMPREF1120_07085*](https://fungidb.org/fungidb/app/record/gene/HMPREF1120_07085) *[Exophiala dermatitidis NIH/UT8656]*  *3' exoribonuclease* [*HMPREF1120_08304*](https://fungidb.org/fungidb/app/record/gene/HMPREF1120_08304)*[Exophiala dermatitidis NIH/UT8656]*  *hypothetical protein* [*HMPREF1120_07255*](https://fungidb.org/fungidb/app/record/gene/HMPREF1120_07255) *[Exophiala dermatitidis NIH/UT8656]*  *hypothetical protein* [*HMPREF1120_07291*](https://fungidb.org/fungidb/app/record/gene/HMPREF1120_07291) *[Exophiala dermatitidis NIH/UT8656]*  *hypothetical protein* [*HMPREF1120_07306*](https://fungidb.org/fungidb/app/record/gene/HMPREF1120_07306) *[Exophiala dermatitidis NIH/UT8656]*  *hypothetical protein* [*HMPREF1120_07433*](https://fungidb.org/fungidb/app/record/gene/HMPREF1120_07433) *[Exophiala dermatitidis NIH/UT8656]*  *hypothetical protein* [*HMPREF1120_07571*](https://fungidb.org/fungidb/app/record/gene/HMPREF1120_07571) *[Exophiala dermatitidis NIH/UT8656]*  *hypothetical protein* [*HMPREF1120_07589*](https://fungidb.org/fungidb/app/record/gene/HMPREF1120_07589) *[Exophiala dermatitidis NIH/UT8656]*  *D-3-phosphoglycerate dehydrogenase* [*HMPREF1120_06805*](https://fungidb.org/fungidb/app/record/gene/HMPREF1120_06805)*[Exophiala dermatitidis NIH/UT8656]*  *ABD1 - mRNA cap guanine-N7 methyltransferase* [*HMPREF1120_06541*](https://fungidb.org/fungidb/app/record/gene/HMPREF1120_06541)*[Exophiala dermatitidis NIH/UT8656]*  *PAN2 - poly(A) specific ribonuclease [Exophiala dermatitidis NIH/UT8656]*  *DOA4 - ubiquitin specific protease* [*HMPREF1120_06573*](https://fungidb.org/fungidb/app/record/gene/HMPREF1120_06573)*[Exophiala dermatitidis NIH/UT8656]*  *RGA2 - Rho-type gtpase-activating protein [Exophiala dermatitidis NIH/UT8656]*  *hypothetical protein* [*HMPREF1120_08236*](https://fungidb.org/fungidb/app/record/gene/HMPREF1120_08236) *[Exophiala dermatitidis NIH/UT8656]*  *cytochrome P450 oxidoreductase* [*HMPREF1120_01361*](https://fungidb.org/fungidb/app/record/gene/HMPREF1120_01361) *[Exophiala dermatitidis NIH/UT8656]*  *hypothetical protein* [*HMPREF1120_08425*](https://fungidb.org/fungidb/app/record/gene/HMPREF1120_08425) *[Exophiala dermatitidis NIH/UT8656]*  *hydrolase* [*HMPREF1120_08460*](https://fungidb.org/fungidb/app/record/gene/HMPREF1120_08460)*[Exophiala dermatitidis NIH/UT8656]*  *hypothetical protein* [*HMPREF1120_08629*](https://fungidb.org/fungidb/app/record/gene/HMPREF1120_08629) *[Exophiala dermatitidis NIH/UT8656]*  *VMA2 - Vacuolar ATP synthase subunit B* [*HMPREF1120_08721*](https://fungidb.org/fungidb/app/record/gene/HMPREF1120_08721) *[Exophiala dermatitidis NIH/UT8656]*  *pyruvate carboxylase* [*HMPREF1120_09185*](https://fungidb.org/fungidb/app/record/gene/HMPREF1120_09185)*[Exophiala dermatitidis NIH/UT8656]*  *hypothetical protein* [*HMPREF1120_08890*](https://fungidb.org/fungidb/app/record/gene/HMPREF1120_08890) *[Exophiala dermatitidis NIH/UT8656]*  *hypothetical protein* [*HMPREF1120_09050*](https://fungidb.org/fungidb/app/record/gene/HMPREF1120_09050) *[Exophiala dermatitidis NIH/UT8656]*  *hypothetical protein* [*HMPREF1120_09084*](https://fungidb.org/fungidb/app/record/gene/HMPREF1120_09084) *[Exophiala dermatitidis NIH/UT8656]*  *chitin synthase* [*HMPREF1120_08777*](https://fungidb.org/fungidb/app/record/gene/HMPREF1120_08777) *[Exophiala dermatitidis NIH/UT8656]*  *hypothetical protein* [*HMPREF1120_09220*](https://fungidb.org/fungidb/app/record/gene/HMPREF1120_09220) *[Exophiala dermatitidis NIH/UT8656]* |  |
| *Ex9 & Ex13*  *27* | *APL5 - AP-3 complex subunit delta [Exophiala dermatitidis NIH/UT8656]*  *G2/mitotic-specific cyclin 3/4 [Exophiala dermatitidis NIH/UT8656]*  *cytochrome P450 oxidoreductase* [*HMPREF1120_01361*](https://fungidb.org/fungidb/app/record/gene/HMPREF1120_01361) *[Exophiala dermatitidis NIH/UT8656]*  *DEAD box RNA helicase HelA* [*HMPREF1120_02010*](https://fungidb.org/fungidb/app/record/gene/HMPREF1120_02010) *[Exophiala dermatitidis NIH/UT8656]*  *hypothetical protein* [*HMPREF1120_02190*](https://fungidb.org/fungidb/app/record/gene/HMPREF1120_02190) *[Exophiala dermatitidis NIH/UT8656]*  *MFS transporter, DHA1 family, multidrug resistance protein* [*HMPREF1120_09017*](https://fungidb.org/fungidb/app/record/gene/HMPREF1120_09017)  *[Exophiala dermatitidis NIH/UT8656]*  *hypothetical protein* [*HMPREF1120_02556*](https://fungidb.org/fungidb/app/record/gene/HMPREF1120_02556) *[Exophiala dermatitidis NIH/UT8656]*  *hypothetical protein* [*HMPREF1120_02949*](https://fungidb.org/fungidb/app/record/gene/HMPREF1120_02949) *[Exophiala dermatitidis NIH/UT8656]*  *hypothetical protein* [*HMPREF1120_03367*](https://fungidb.org/fungidb/app/record/gene/HMPREF1120_03367) *[Exophiala dermatitidis NIH/UT8656]*  *VPS41 - Vacuolar protein sorting-associated protein 41[Exophiala dermatitidis NIH/UT8656]*  *MFS transporter, SP family, sugar:H+ symporter* [*HMPREF1120_06771*](https://fungidb.org/fungidb/app/record/gene/HMPREF1120_06771) *[Exophiala dermatitidis NIH/UT8656]*  *hypothetical protein* [*HMPREF1120_04659*](https://fungidb.org/fungidb/app/record/gene/HMPREF1120_04659) *[Exophiala dermatitidis NIH/UT8656]*  *DNA repair protein RAD50* [*HMPREF1120_04505*](https://fungidb.org/fungidb/app/record/gene/HMPREF1120_04505) *[Exophiala dermatitidis NIH/UT8656]*  *biphenyl-2,3-diol 1,2-dioxygenase, variant* [*HMPREF1120_05880*](https://fungidb.org/fungidb/app/record/gene/HMPREF1120_05880) *[Exophiala dermatitidis NIH/UT8656]*  *chloride channel 3 [Exophiala dermatitidis NIH/UT8656]*  *hypothetical protein* [*HMPREF1120_06002*](https://fungidb.org/fungidb/app/record/gene/HMPREF1120_06002) *[Exophiala dermatitidis NIH/UT8656]*  *hypothetical protein* [*HMPREF1120_06122*](https://fungidb.org/fungidb/app/record/gene/HMPREF1120_06122) *[Exophiala dermatitidis NIH/UT8656]*  *hypothetical protein* [*HMPREF1120_06336*](https://fungidb.org/fungidb/app/record/gene/HMPREF1120_06336) *[Exophiala dermatitidis NIH/UT8656]*  *ISU1 - iron-binding protein* [*HMPREF1120_06751*](https://fungidb.org/fungidb/app/record/gene/HMPREF1120_06751) *[Exophiala dermatitidis NIH/UT8656]*  *LBA1 - Regulator of nonsense transcripts 1-like protein [Exophiala dermatitidis NIH/UT8656]*  *hypothetical protein* [*HMPREF1120_07292*](https://fungidb.org/fungidb/app/record/gene/HMPREF1120_07292) *[Exophiala dermatitidis NIH/UT8656]*  *adenosinetriphosphatase* [*HMPREF1120_09246*](https://fungidb.org/fungidb/app/record/gene/HMPREF1120_09246) *[Exophiala dermatitidis NIH/UT8656]*  *TPC1 - mitochondrial thiamine pyrophosphate transporter* [*HMPREF1120_07619*](https://fungidb.org/fungidb/app/record/gene/HMPREF1120_07619) *[Exophiala dermatitidis NIH/UT8656]*  *MFS transporter, SIT family, siderophore-iron:H+ symporter* [*HMPREF1120_07838*](https://fungidb.org/fungidb/app/record/gene/HMPREF1120_07838) *[Exophiala dermatitidis NIH/UT8656]*  *hypothetical protein* [*HMPREF1120_08665*](https://fungidb.org/fungidb/app/record/gene/HMPREF1120_08665) *[Exophiala dermatitidis NIH/UT8656]* |  |
| *Ex11 & Ex13*  *27* | *APL5 - AP-3 complex subunit delta* [*HMPREF1120_04507*](https://fungidb.org/fungidb/app/record/gene/HMPREF1120_04507) *[Exophiala dermatitidis NIH/UT8656]*  *G2/mitotic-specific cyclin 3/4* [*HMPREF1120_00797*](https://fungidb.org/fungidb/app/record/gene/HMPREF1120_00797) *[Exophiala dermatitidis NIH/UT8656]*  *cytochrome P450 oxidoreductase* [*HMPREF1120_01361*](https://fungidb.org/fungidb/app/record/gene/HMPREF1120_01361) *[Exophiala dermatitidis NIH/UT8656]*  *DEAD box RNA helicase HelA* [*HMPREF1120_02010*](https://fungidb.org/fungidb/app/record/gene/HMPREF1120_02010) *[Exophiala dermatitidis NIH/UT8656]*  *hypothetical protein* [*HMPREF1120_02190*](https://fungidb.org/fungidb/app/record/gene/HMPREF1120_02190) *[Exophiala dermatitidis NIH/UT8656]*  *MFS transporter, DHA1 family, multidrug resistance protein* [*HMPREF1120_09017*](https://fungidb.org/fungidb/app/record/gene/HMPREF1120_09017) *[Exophiala dermatitidis NIH/UT8656]*  *hypothetical protein* [*HMPREF1120_02556*](https://fungidb.org/fungidb/app/record/gene/HMPREF1120_02556) *[Exophiala dermatitidis NIH/UT8656]*  *hypothetical protein* [*HMPREF1120_02949*](https://fungidb.org/fungidb/app/record/gene/HMPREF1120_02949) *[Exophiala dermatitidis NIH/UT8656]*  *hypothetical protein* [*HMPREF1120_03367*](https://fungidb.org/fungidb/app/record/gene/HMPREF1120_03367) *[Exophiala dermatitidis NIH/UT8656]*  *VPS41 - Vacuolar protein sorting-associated protein 41* [*HMPREF1120_06737*](https://fungidb.org/fungidb/app/record/gene/HMPREF1120_06737) *[Exophiala dermatitidis NIH/UT8656]*  *MFS transporter, SP family, sugar:H+ symporter* [*HMPREF1120_09186*](https://fungidb.org/fungidb/app/record/gene/HMPREF1120_09186)  *[Exophiala dermatitidis NIH/UT8656]*  *hypothetical protein* [*HMPREF1120_04659*](https://fungidb.org/fungidb/app/record/gene/HMPREF1120_04659) *[Exophiala dermatitidis NIH/UT8656]*  *DNA repair protein RAD50* [*HMPREF1120_04505*](https://fungidb.org/fungidb/app/record/gene/HMPREF1120_04505) *[Exophiala dermatitidis NIH/UT8656]*  *biphenyl-2,3-diol 1,2-dioxygenase, variant* [*HMPREF1120_05880*](https://fungidb.org/fungidb/app/record/gene/HMPREF1120_05880)  *[Exophiala dermatitidis NIH/UT8656]*  *chloride channel 3* [*HMPREF1120_05920*](https://fungidb.org/fungidb/app/record/gene/HMPREF1120_05920) *[Exophiala dermatitidis NIH/UT8656]*  *hypothetical protein* [*HMPREF1120_06002*](https://fungidb.org/fungidb/app/record/gene/HMPREF1120_06002) *[Exophiala dermatitidis NIH/UT8656]*  *hypothetical protein* [*HMPREF1120_06122*](https://fungidb.org/fungidb/app/record/gene/HMPREF1120_06122) *[Exophiala dermatitidis NIH/UT8656]*  *hypothetical protein* [*HMPREF1120_06336*](https://fungidb.org/fungidb/app/record/gene/HMPREF1120_06336) *[Exophiala dermatitidis NIH/UT8656]*  *ISU1 - iron-binding protein* [*HMPREF1120_06751*](https://fungidb.org/fungidb/app/record/gene/HMPREF1120_06751) *[Exophiala dermatitidis NIH/UT8656]*  *LBA1 - Regulator of nonsense transcripts 1-like protein [Exophiala dermatitidis NIH/UT8656]*  *hypothetical protein* [*HMPREF1120_07292*](https://fungidb.org/fungidb/app/record/gene/HMPREF1120_07292) *[Exophiala dermatitidis NIH/UT8656]*  *adenosinetriphosphatase* [*HMPREF1120_09246*](https://fungidb.org/fungidb/app/record/gene/HMPREF1120_09246) *[Exophiala dermatitidis NIH/UT8656]*  *hypothetical protein A1O3_09068 [Capronia epimyces CBS 606.96]*  *TPC1 - mitochondrial thiamine pyrophosphate transporter* [*HMPREF1120_07619*](https://fungidb.org/fungidb/app/record/gene/HMPREF1120_07619) *[Exophiala dermatitidis NIH/UT8656]*  *MFS transporter, SIT family, siderophore-iron:H+ symporter* [*HMPREF1120_07838*](https://fungidb.org/fungidb/app/record/gene/HMPREF1120_07838) *[Exophiala dermatitidis NIH/UT8656]*  *hypothetical protein* [*HMPREF1120_08665*](https://fungidb.org/fungidb/app/record/gene/HMPREF1120_08665) *[Exophiala dermatitidis NIH/UT8656]* |  |
