# Supplementary figures and images for: In host evolution of Exophiala dermatitidis in cystic fibrosis lung micro-environment
Source: G3 (Bethesda). 2023 Jun 9;13(8):jkad126. doi: 10.1093/g3journal/jkad126 (PMC10484061; doi:10.1093/g3journal/jkad126)

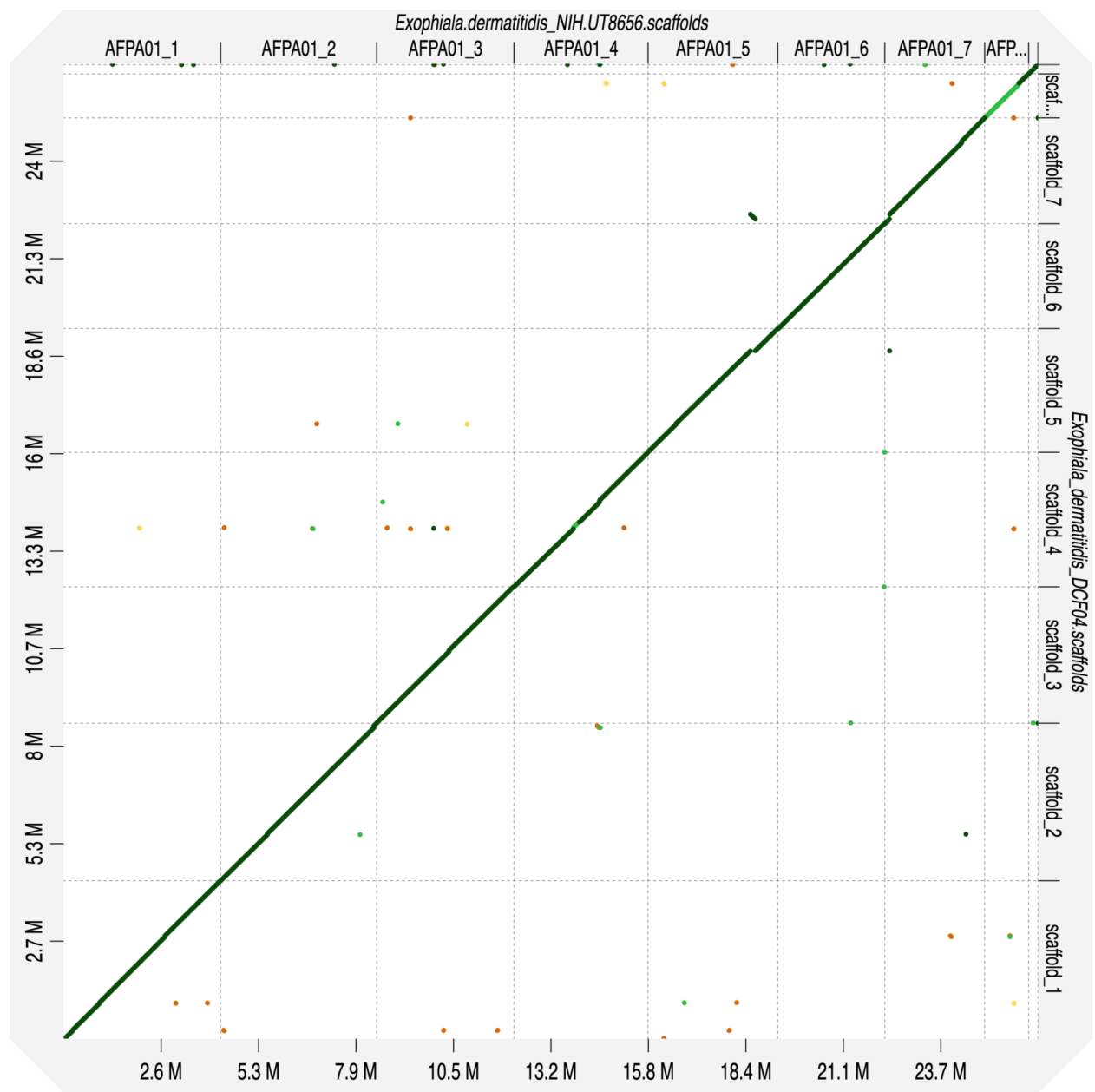

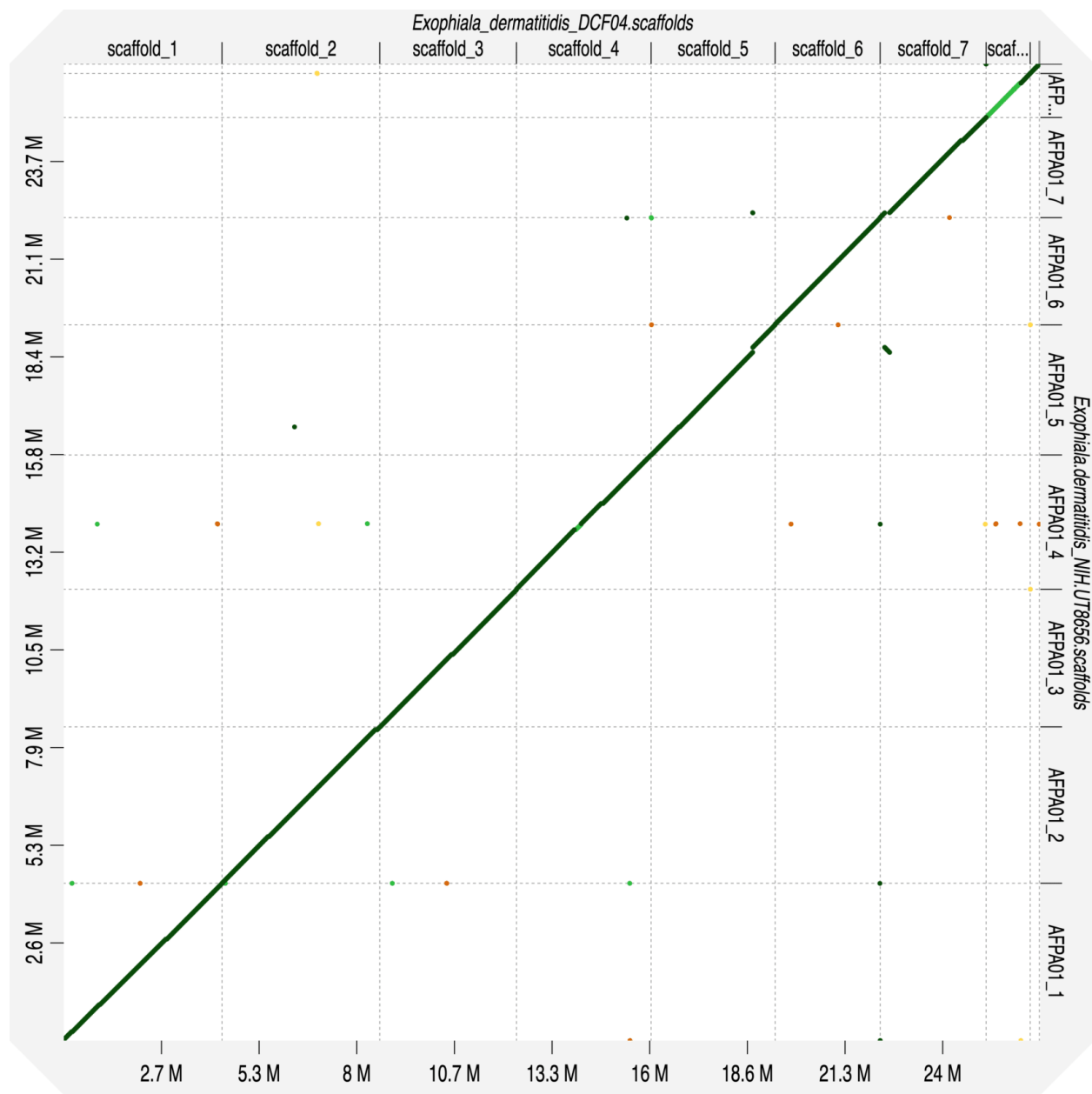

Supplement: jkad126_Supplementary_Data [file jkad126_supplementary_data.zip › Supplemental_Figure_1_G3-2023-404223.pdf]

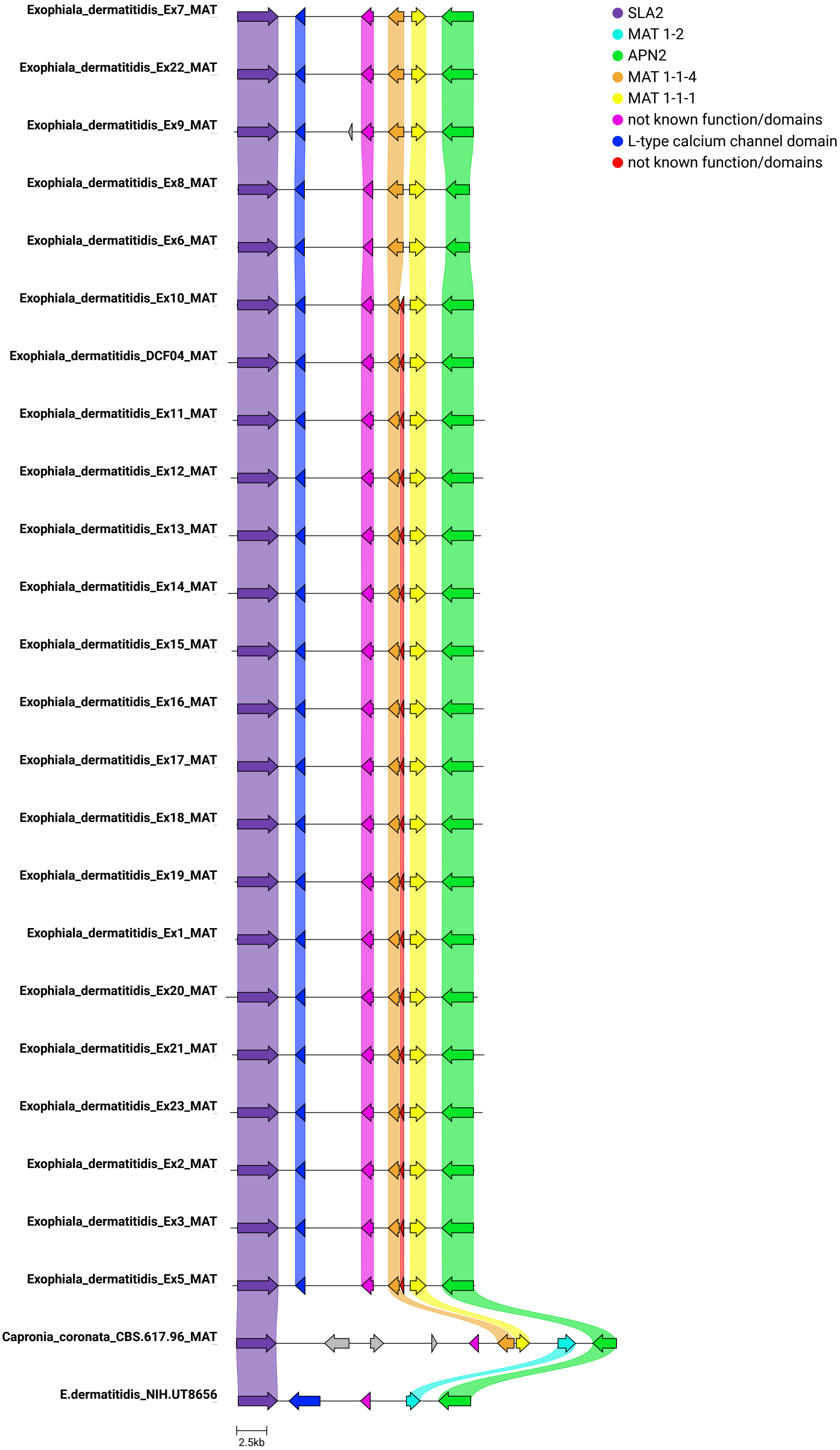

Supplement: jkad126_Supplementary_Data [file jkad126_supplementary_data.zip › Supplemental_Figure_2_G3-2023-404223.pdf]

Tree scale: 0.1

**Colored ranges**

Clade I

Clade II

Clade III

Root

**Legend**

Early

Late

Root

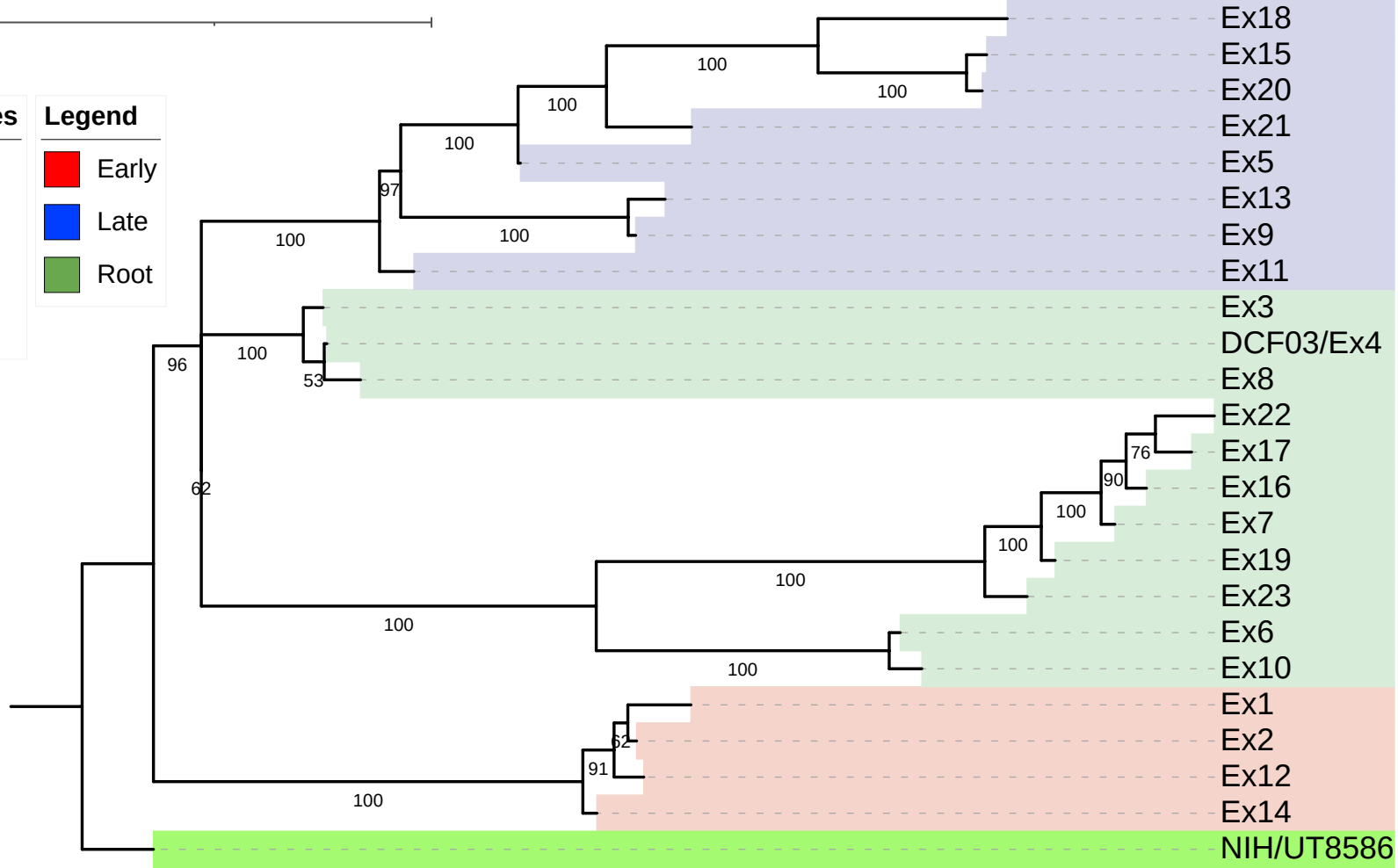

Supplement: jkad126_Supplementary_Data [file jkad126_supplementary_data.zip › Supplemental_Figure_3_G3-2023-404223.pdf]
